# Supplementary material for: The 3D enhancer network of the developing T cell genome is shaped by SATB1
Source: Nat Commun. 2022 Nov 14;13:6954. doi: 10.1038/s41467-022-34345-y (PMC9663569; doi:10.1038/s41467-022-34345-y)
Supplement: Supplementary file 1 — Supplementary Information [file 41467_2022_34345_MOESM1_ESM.pdf]

SUPPLEMENTARY INFORMATION

The 3D enhancer network of the developing T cell genome is shaped by SATB1

Tomas Zelenka[1,2,3], Antonios Klonizakis[1], Despina Tsoukatou[2], Dionysios-Alexandros Papamatheakis[1,2], Sören Franzenburg[4], Petros Tzerpos[1,5], Ioannis-Rafail Tzonevrakis[1], George Papadogkonas[1,2], Manuela Kapsetaki[2], Christoforos Nikolaou[1,2,6], Dariusz Plewczynski[7,8], Charalampos Spilianakis[1,2*]

[1] Department of Biology, University of Crete, Heraklion, Crete, Greece

[2] Institute of Molecular Biology and Biotechnology—Foundation for Research and Technology Hellas, Heraklion, Crete, Greece

[3] Department of Immunology, H. Lee Moffitt Cancer Center and Research Institute, Tampa, FL, USA (current address)

[4] University Hospital Schleswig Holstein, Kiel, Germany

[5] Department of Biochemistry and Molecular Biology, Faculty of Medicine, University of Debrecen, Debrecen, HU-4032 Hungary (current address)

[6] Institute for Bioinnovation, Biomedical Sciences Research Centre "Alexander Fleming," 16672 Vari, Greece (current address)

[7] Laboratory of Bioinformatics and Computational Genomics, Faculty of Mathematics and Information Science, Warsaw University of Technology, Warsaw, Poland

[8] Laboratory of Functional and Structural Genomics, Centre of New Technologies, University of Warsaw, Warsaw, Poland

[*] Corresponding author, email: spiliana@imbb.forth.gr

Supplementary Figures

Supplementary Figure 1

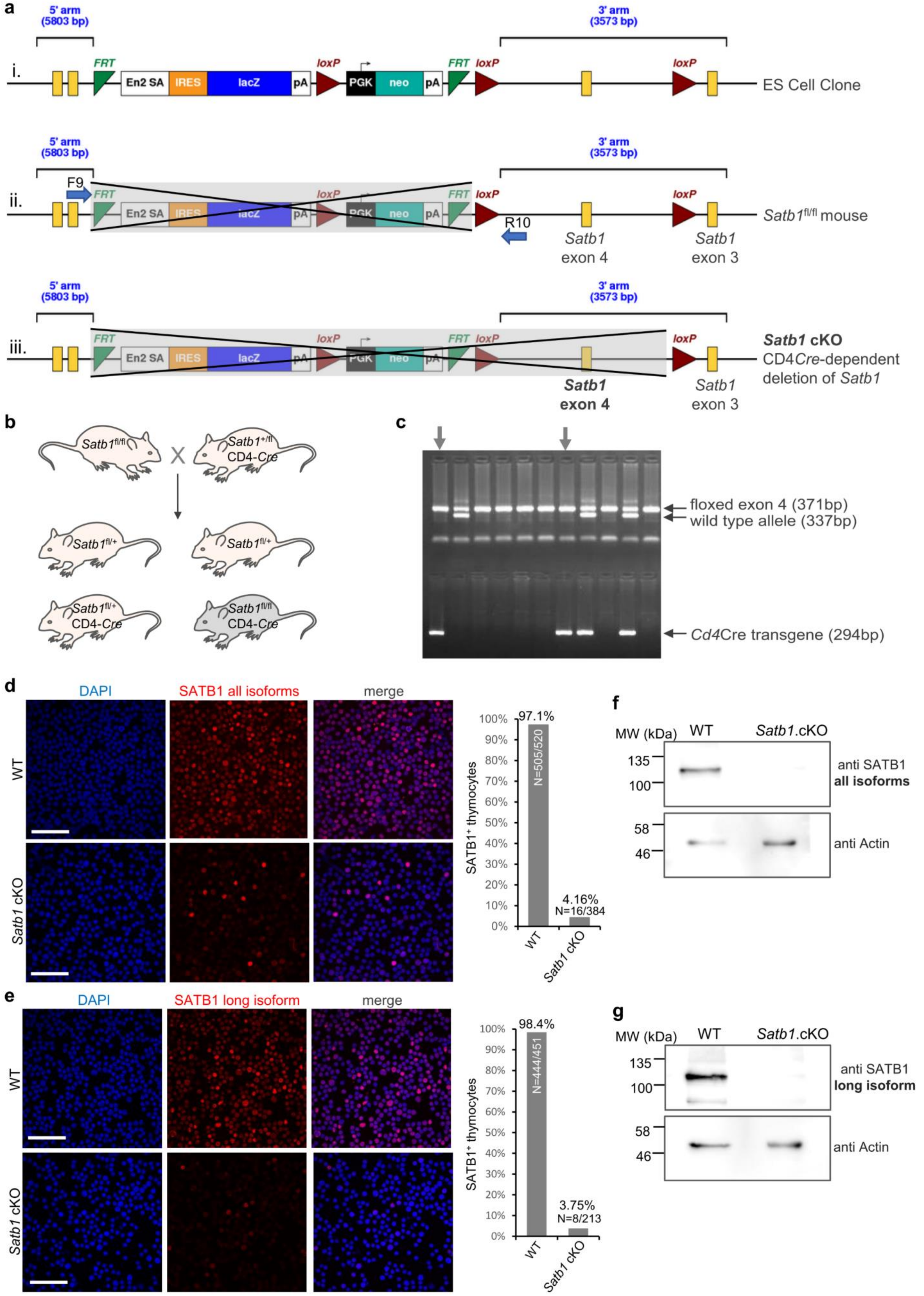

Supplementary Fig. 1. **Generation of a tissue specific *Satb1* knockout mouse. a** i. ES cell clone HEPD0534_5_E11 obtained from EUCOMM, with knockout first mutation, ii. mouse with floxed exon 4 allele obtained from Vassilis Pachnis' laboratory[1] where the reporter cassete has been removed *in vitro* (the primer set utilized for genotyping is indicated, F9/R10), iii. *Satb1* allele upon deletion of floxed exon 4 *Satb1* allele in transgenic mice expressing the Cre recombinase driven by the *Cd4* gene promoter. **b** Breading scheme for the generation of tissue-specific *Satb1* knockout mice (grey). *Satb1*$^{fl/fl}$ mice were bred to *Satb1*$^{+/fl}$ mice expressing the CD4-Cre transgene[2]. Figure created with Motifolio Toolkit (Motifolio Inc, Ellicott City, Md). **c** Genotyping of the offspring indicated in **b**. This is a genotyping experiment performed for each offspring of the breeding scheme indicated in b. Therefore the genotyping PCR experiment has been performed for hundreds of times. **d** Immunofluorescence experiments indicating that SATB1 is expressed in 97.11% of total wild type thymocytes and 4.16% of total *Satb1* cKO thymocytes, utilizing an antibody detecting all SATB1 isoforms. The relative SATB1 fluorescence signal was calculated with Volocity (PerkinElmer). Blue DAPI, scale bar 50 μm. **e** The same experiment as in **d**, but utilizing an antibody against the long SATB1 isoform. **f** Western blot experiment utilizing an antibody against all SATB1 isoforms performed in WT and *Satb1* cKO thymocytes. The Western blot analysis was performed four times for anti-SATB1 long and four times for anti-SATB1 all isoforms, with similar results. **g** The same experiment as in **f** but utilizing an antibody against the long SATB1 isoform.

Supplementary Figure 2

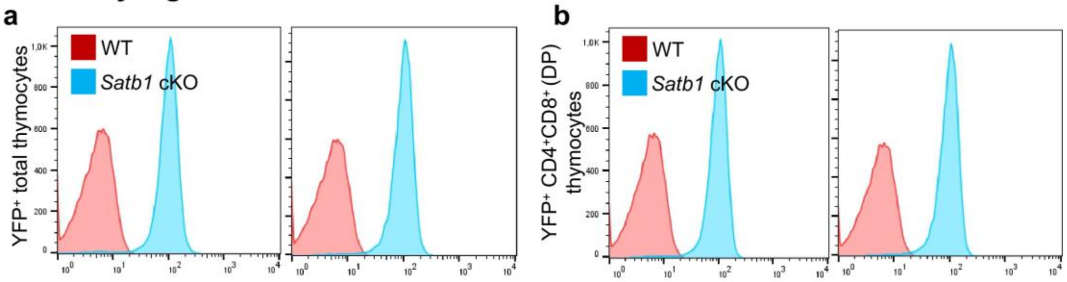

Total thymocytes	YFP MFI	YFP⁺ cells (%)
WT	5.29	0.0
Satb1 cKO1	99.6	96.3
Satb1 cKO2	98.4	97.0

$CD4^+CD8^+$ (DP)	YFP MFI	YFP⁺ cells (%)
WT	5.5	0.0
Satb1 cKO1	98.0	97.0
Satb1 cKO2	97.1	97.4

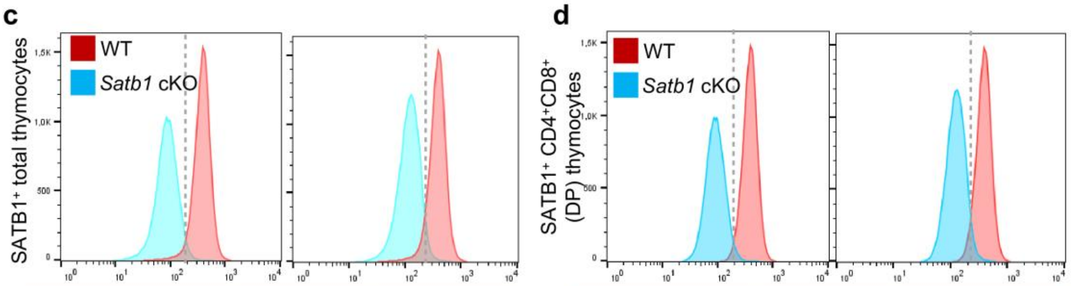

Total thymocytes	SATB1 MFI	SATB1⁺ (%)
WT	373	94.2
Satb1 cKO1	184	2.83
Satb1 cKO2	212	5.4

$CD4^+CD8^+$ (DP)	SATB1 MFI	SATB1⁺ (%)
WT	377	97.15
Satb1 cKO1	191	2.7
Satb1 cKO2	213	3.6

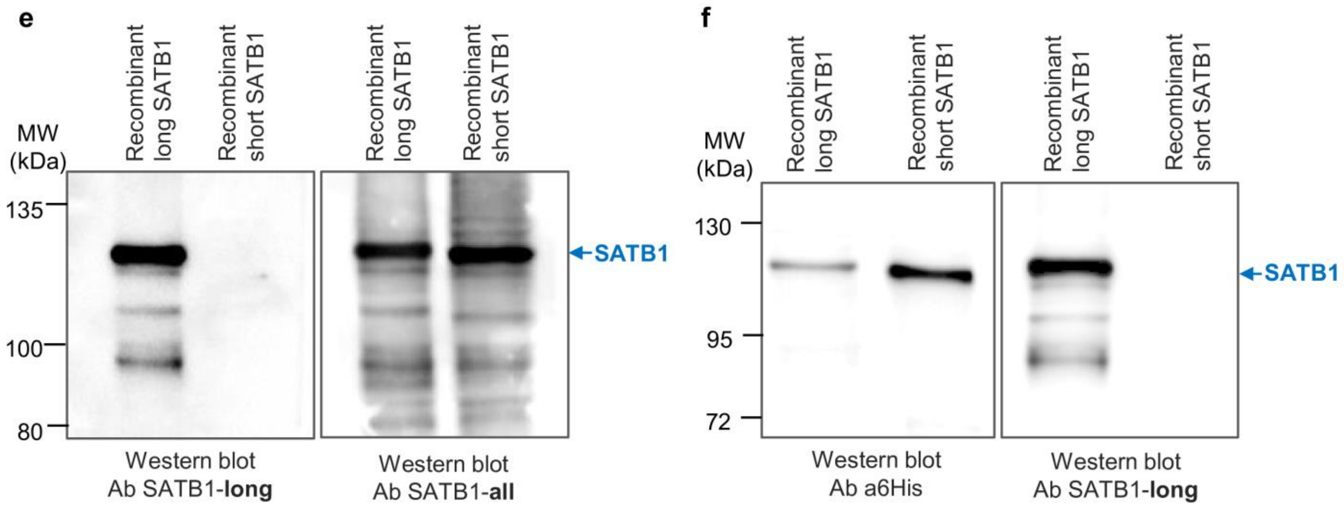

Supplementary Fig. 2. **Efficiency of SATB1 depletion in *Satb1* cKO thymocytes. a** More than 96% of the total *Satb1* cKO thymocytes express the YFP reporter, indicating the efficient expression of the Cre recombinase expressed under the *Cd4* promoter. The generation of mice expressing the YFP reporter gene has been previously described[3]. The primers used to confirm the presence of the YFP reporter were Rosa26-LC 5'-GCT CTG AGT TGT TAT CAG TAA GG-3', Rosa26-R3 5'-GGA GCG GGA GAA ATG GAT ATG-3' and Rosa26-R2 5'-GCG AAG AGT TTG TCC TCA ACC-3'. **b** More than 97% of the double positive ($CD4^+CD8^+$) *Satb1* cKO thymocytes express the YFP reporter, indicating the efficient expression of the Cre recombinase expressed under the *Cd4* promoter. **c** Intracellular staining for SATB1 protein in total thymocytes and FACS analysis. **d** Intracellular staining for SATB1 protein in double positive ($CD4^+CD8^+$) thymocytes and FACS analysis. For figures **a**, **b**, **c**, **d**; two biological

replicates are shown. **e** The SATB1 short and long protein isoforms were expressed in bacteria and detected by Western blot analysis using an antibody against the long SATB1 isoform (left panel) and an antibody non-specifically targeting all SATB1 isoforms (right panel). The Western blot analysis was performed seven times with similar results. **f** Recombinant short and long SATB1 protein isoforms with a 6xHis histidine tag were expressed in bacteria and detected by Western blot using anti-6xHis antibody (left panel) or an anti-long SATB1 isoform antibody (right panel). The Western blot analysis was performed three times with similar results.

Supplementary Figure 3

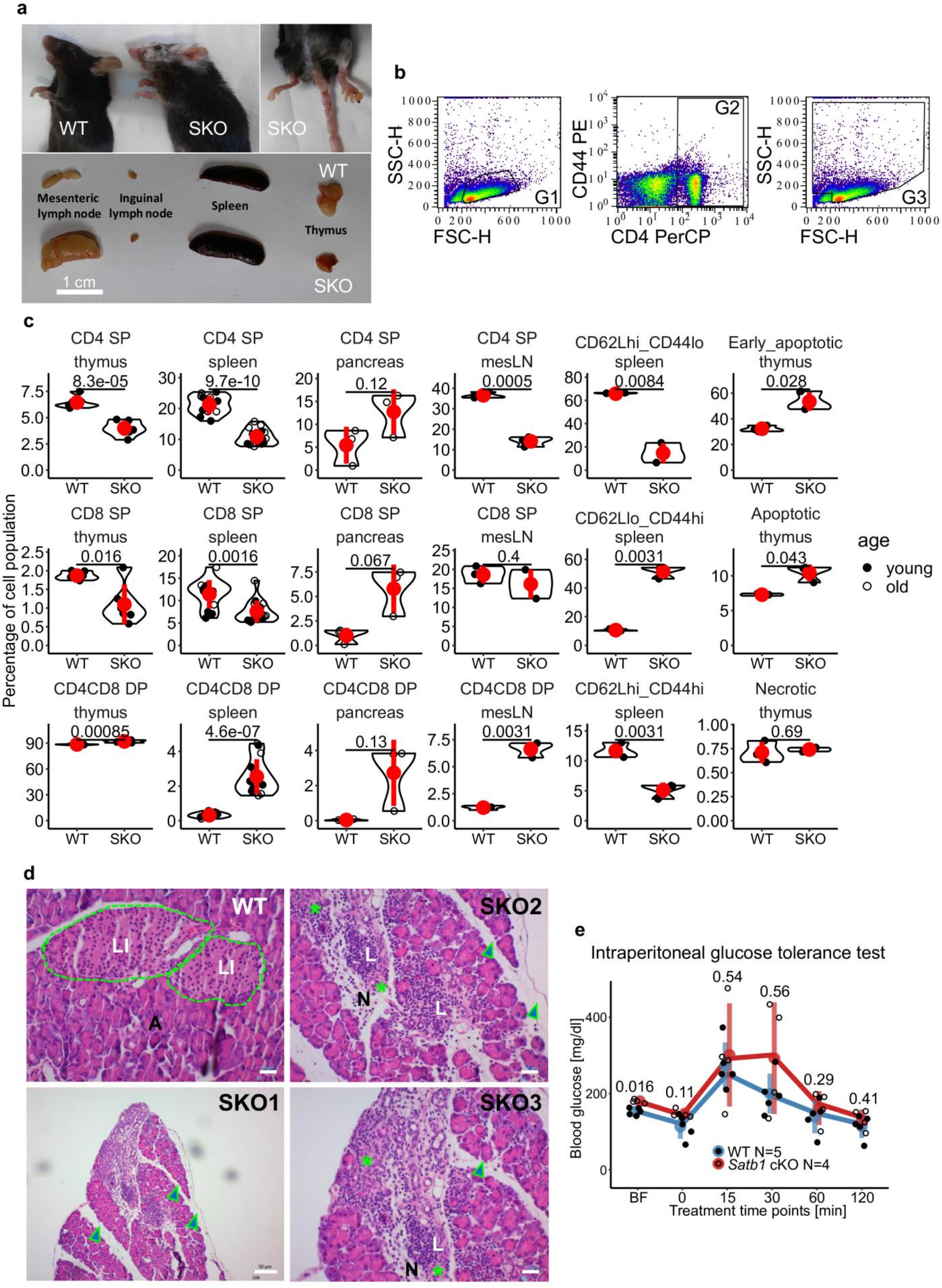

Supplementary Fig. 3. **Deregulation of the immune system in *Satb1* cKO animals. a** Phenotypical comparison of WT and *Satb1* cKO mice (SKO). **b** Gating strategies for flow cytometry experiments. **c** Characterization of cell populations in WT and *Satb1* cKO by flow cytometry. Young animals (45±11 days; 6 WT, 6 *Satb1* cKO) were used for the analysis of the thymus and spleen and old animals (179±35 days; 7 WT, 9 *Satb1* cKO) for the analysis of spleen. Three young animals for each genotype were used for CD62 / CD44 analysis of the spleen and three old animals for CD4 / CD8 analysis of the pancreas. The red circle represents the mean ± s.d. *P* values by two-sided Student's t-test. **d** T cells in *Satb1* cKO animals infiltrated in the pancreas (see **c**) and damaged the islets of Langerhans. Pancreas tissue section experiments stained with Hematoxylin-Eosin to visualize the islets of Langerhans (LI, marked with a dotted line) surrounded by acinar cells. In *Satb1* cKO (SKO) sections, the islets of Langerhans and acinar regions were infiltrated by lymphocytes (L; quantified in **c**), contained regions of fibrosis (asterisks), necrosis (N) and sub-capsular and interlobular edema, manifested with dilated ducts (arrowheads). Scale bar for WT, SKO2, SKO3 is 20 μm, and for SKO1 50 μm. **e** Intraperitoneal glucose tolerance test to indicate impaired glucose metabolism in *Satb1* cKO animals. BF indicates steady-state glucose levels, before a 6 hour fasting period. Red and blue circles represent the mean ± s.d. The lines connecting circles represent the median values.

Supplementary Figure 4

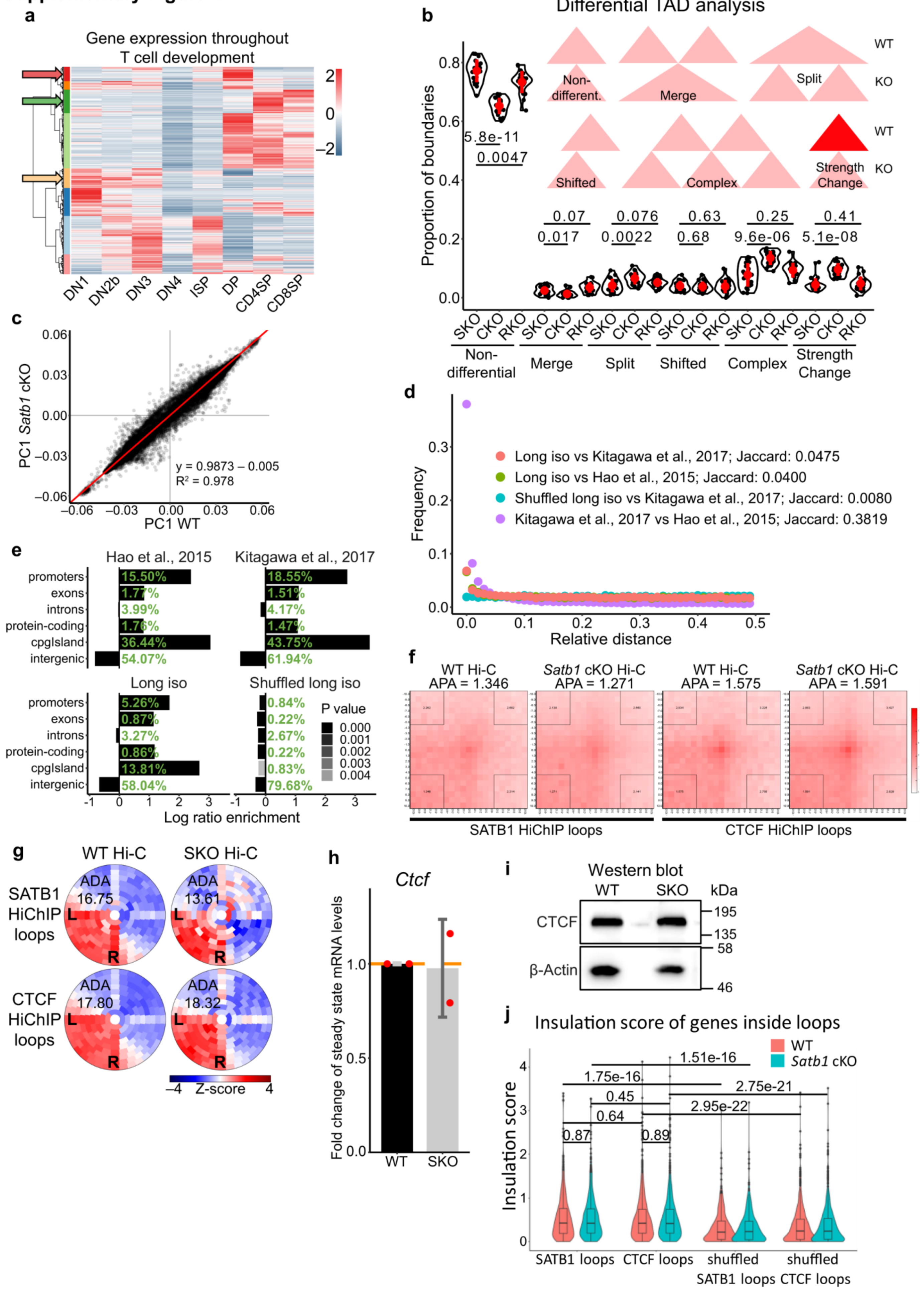

Supplementary Fig. 4. **Characterization of chromatin architecture changes in *Satb1* cKO animals. a** Heatmap of normalized RNA-seq counts of sorted DN1, DN2b, DN3, DN4, ISP, DP, CD4SP and CD8SP T cell subset populations (GSE109125[4]). Three clusters were selected as DN, DP and SP signature genes (orange, red and green arrows, respectively; Supplementary Data 3). **b** Differential analysis of topologically associating domains using TADCompare[5] at 100 kbp resolution between WT and factor-depleted cells (combined biological replicates). TAD categories are explained by a graphical legend within the figure. SKO represents *Satb1*$^{fl/fl}$*Cd4*-Cre^{+} murine thymocytes, RKO –*Rad21*$^{fl/fl}$*Cd4*-Cre^{+} DP murine thymocytes[6] and CKO – CTCF-depleted (AID degron system) mESCs[7]. Black dots represent different TAD categories for individual chromosomes (n=20). The red circles represent the mean ± s.d. P values by two-sided Wilcoxon rank sum test, non-adjusted for multiple comparisons. **c** Analysis of A/B compartments performed according to the original protocol[8] at 100,000 resolution of Hi-C datasets using HOMER[9]. Principal components of WT and *Satb1* cKO annotated regions were plotted against each other to show the proportion of compartment switch. **d** Long SATB1 isoform binding sites were spatially correlated with two publicly available SATB1 ChIP-seq datasets[10,11] (both of which utilized antibodies targeting all SATB1 isoforms). Data are based on the Relative Distance metric of Bedtools[12,13], therefore the relative distance represents a ratio between the smaller distance between a feature from dataset A and an upstream or downstream feature from dataset B, and the overall distance between the downstream and upstream features from dataset B. Jaccard statistic is also provided. **e** Genomic feature association analysis (Genome Ontology) for the three datasets of SATB1 binding sites, analyzed by the AnnotatePeak function of Homer[9]. Numbers represent the absolute % overlap of features with SATB1 binding sites. In figures **d**, **e**; the "Long iso" dataset represents the HiChIP-derived binding sites of the long SATB1 isoform used in this study. The "Shuffled long iso" dataset represents a negative control, i.e. randomly shuffled binding sites of the long SATB1 isoform. **f** Aggregate peak analysis[14] was calculated and visualized by Juicer Tools[15] to show that SATB1 HiChIP loops had stronger signal in WT Hi-C datasets than in *Satb1* cKO Hi-C. CTCF HiChIP loops retained the same APA score indicating that CTCF-based high order chromatin organization remained unchanged in *Satb1* cKO. **g** Intra-domain interaction frequencies of Hi-C matrices derived from WT and *Satb1* cKO (SKO) thymocytes, restricted to the SATB1/CTCF-dependent HiChIP loops. Provided bullseye visualization by SIPMeta[16] represents a transformed rectangular heatmap such that each bin's Euclidean distance to the center directly corresponds to its Manhattan distance in the original map. Each ring in the bullseye plot has segments corresponding to the $4 \times N$ bins with a Manhattan distance of N from the central bin. Each bin in a ring takes up exactly the same angular area and they are evenly distributed around the circle. Z-score transformation is done for each ring separately and the aggregate domain analysis (ADA) score was obtained by percentage of Z-scores > 1 in the bottom left quarter vs the total plot. L and R denotes left and right loop anchors, respectively. Note the disturbed interaction pattern within SATB1-dependent and not within CTCF-dependent loops in *Satb1* cKO cells. **h** Relative steady state *Ctcf* mRNA levels in WT and *Satb1* cKO thymocytes detected by RT-qPCR. Results are from 2 biological replicates and the qPCR was performed in triplicates. The relative mRNA expression level was corrected based on *Hprt1* expression in WT and *Satb1* cKO thymocytes samples. Values were calculated based on the ΔΔCt method and they are presented as fold change in *Satb1* cKO (SKO) over WT. Data are presented as mean values ± s.d. **i** Western blot analysis probing CTCF protein levels in WT and *Satb1* cKO (SKO) thymocytes. The Western blot analysis was performed two times with similar results. **j** Transcriptional insulation scores calculated as described in the methods section showed that expression of genes inside SATB1 and CTCF loops was different from genes outside the loops. Both SATB1 and CTCF displayed a similar insulation effect which was significantly different between the loops and randomly shuffled loops in both WT and *Satb1* cKO. *P* values by two-sided Wilcoxon rank sum test, non-adjusted for multiple comparisons. The boxplots show median with the top and bottom edges of the box representing the 75th and 25th percentiles, respectively. The whiskers represent the most extreme values that are within 1.5 times the interquartile range of the 25th and 75th percentiles. Outliers outside the whiskers are shown as dots. The exact number of features analyzed can be found in the source data.

Supplementary Figure 5

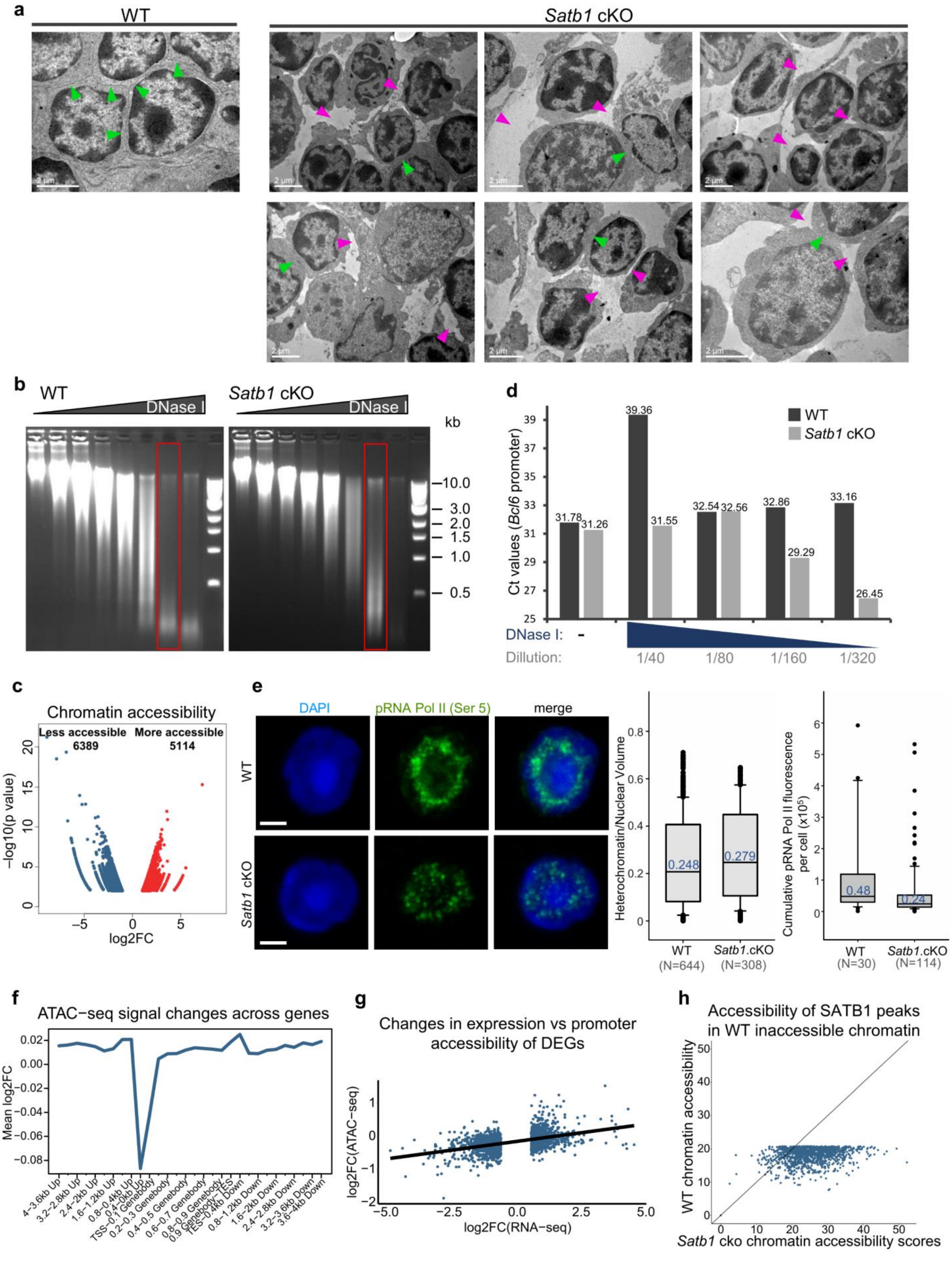

Supplementary Fig. 5. **Dysregulated chromatin accessibility in *Satb1* cKO animals. a** Representative transmission electron microscopy images of WT and *Satb1* cKO thymus sections indicating dysregulation of heterochromatin/euchromatin arrangement in *Satb1* cKO thymocytes and also supporting the impairment in intercellular contacts. Green and magenta arrows show examples of intact and disrupted intercellular contacts, respectively. Scale bar 2 μm. Data are representative of two biological replicates with similar results. **b** Gel electrophoresis of genomic DNA from WT and *Satb1* cKO thymocytes treated with increasing concentration of DNase I. The red rectangle indicates that for the same DNase I concentration the WT chromatin is digested more and therefore is considered more accessible compared to the *Satb1* cKO thymocyte chromatin. One replicate was performed to support the results from the ATAC-seq analysis. **c** Volcano plot of differentially accessible regions in WT and *Satb1* cKO as determined by ATAC-seq. **d** *Satb1* cKO thymocyte chromatin is less accessible to DNase I. Nuclei from WT and *Satb1* cKO thymocytes were either not treated or treated with increasing concentration of DNase I. Genomic DNA was prepared and qPCR was performed with a pair of primers designed for a genomic region of the *Bcl6* promoter encompassing a SATB1 binding site which displayed differential chromatin accessibility based on ATAC-seq experiments. Ct values for the pair of primers hybridizing on the *Bcl6* promoter were corrected based on the Ct values acquired for a control non-SATB1 bound region. **e** LEFT: Immunofluorescence experiments for the detection of the phosphorylated form of RNA polymerase II (phosphorylated Ser 5 of CTD, CTD4H8-Alexa Fluor labeled; Covance, A488-128L) in WT and *Satb1* cKO thymocytes. Blue DAPI, scale bar 2 μm. MIDDLE: The cumulative DAPI signal detected per cell (y-axis) measured in three independent experiments with confocal microscopy. RIGHT: The cumulative fluorescence of phosphorylated RNA polymerase II per cell (y-axis) indicated in each box plot is characterized by box plots. For MIDDLE and RIGHT, the boundary of the box closest to zero indicates the 25th percentile, the line within the box marks the median, and the boundary of the box farthest from zero indicates the 75th percentile. Whiskers (error bars) above and below the box indicate the 90th and 10th percentiles. Single cell outliers (5% of total signals) are indicated in the vicinity outside the whiskers. Box plots were created in Sigma Plot 12. **f** Log2 fold change of ATAC-seq differential chromatin accessibility between WT and *Satb1* cKO plotted along genes ± 4 kbp. Graph depicts the drop in chromatin accessibility in *Satb1* cKO at the TSS of genes, supporting a regulatory role for SATB1. **g** Correlation between RNA levels and promoter chromatin accessibility changes (–2 kbp – TSS) of *Satb1* cKO differentially expressed genes (DEGs; Spearman's $\rho = 0.438$, $P = 4.07e\text{-}88$). **h** Regions with low chromatin accessibility in WT had mostly increased chromatin accessibility in *Satb1* cKO. The average of log10 transformed read-normalized accessibility scores of ten randomizations depicted in Fig. 4a was used as a cutoff to determine SATB1 peaks with low accessibility levels.

Supplementary Figure 6

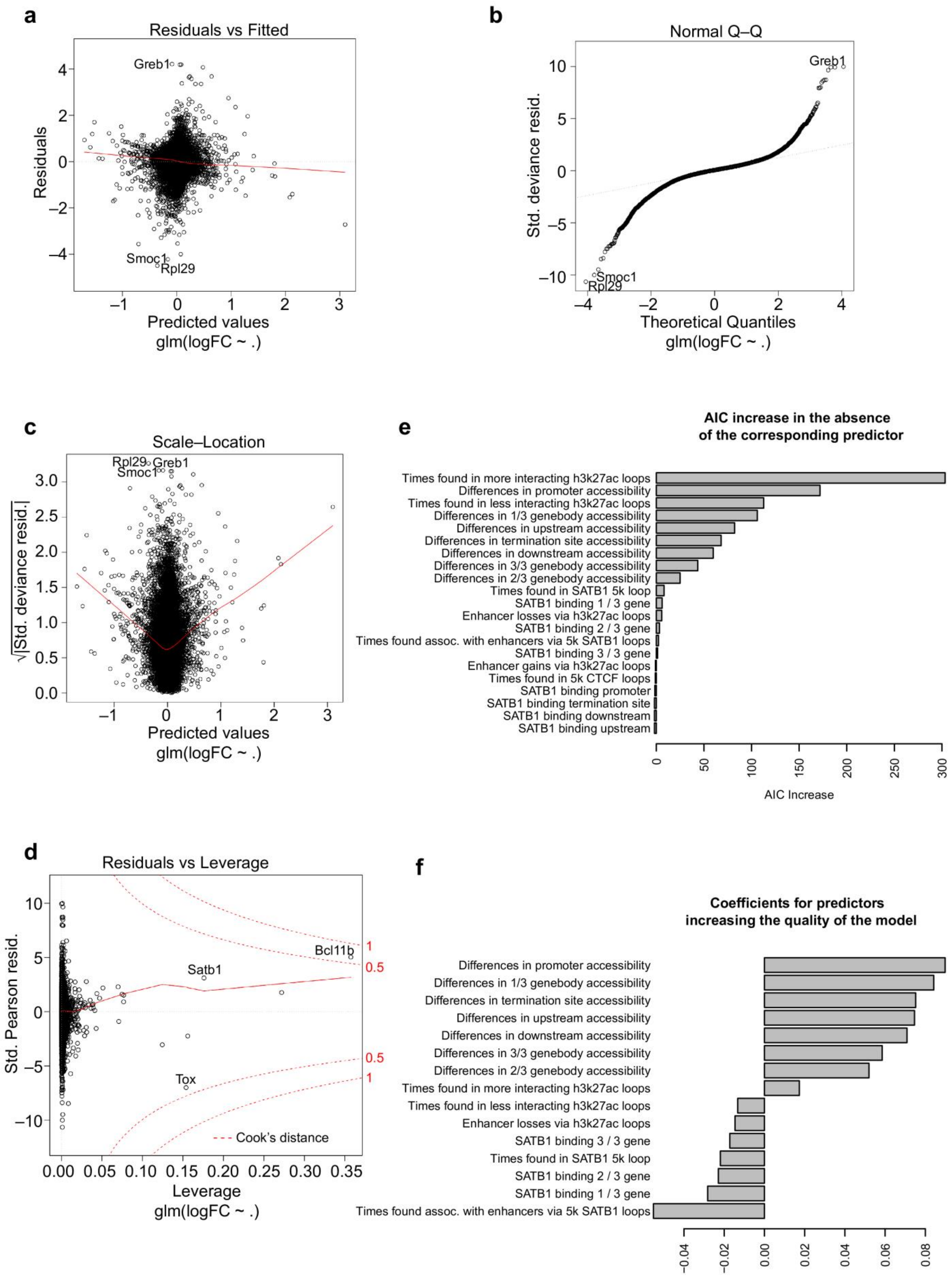

Supplementary Fig. 6. **Linear regression model. a** Diagnostic plot for residuals vs fitted values. Non-linear relationships between predictor variables and logFC changes were evaluated. Values were distributed randomly across a horizontal line with no obvious pattern arising, indicating that assumptions of the regression model were fulfilled well. **b** Q-Q plot of residuals. The plot evaluates whether the residuals were normally distributed. Deviations from the normal distribution were observed for a set of residuals. **c** Diagnostic plot for homoscedasticity. Model assumptions are not violated if data points are randomly distributed. No clear patterns were observed, indicating a good fit to the model assumptions. **d** Diagnostic plot for influential outliers based on the Cook's distance. No gene was positioned outside the dashed line corresponding to the Cook's distance of 1. As such no gene was excluded when constructing the model. **e** Evaluation of the quality of each predictor. A regression model was first constructed using all the predictors. For each predictor, a new model was constructed utilizing all predictors beside the one studied. The Akaike Information Criterion (AIC) of the old model was subtracted from the AIC of the new model. An increase in AIC after the removal of a predictor indicated that the model lacking that predictor performed worse than the original one. The final model included only the predictors that were associated with an increase in AIC after their removal. **f** Coefficients of final predictors. Positive values indicate that a variable was linked with an increase in RNA levels in the *Satb1* cKO cells, while negative values indicate the opposite.

Supplementary Figure 7

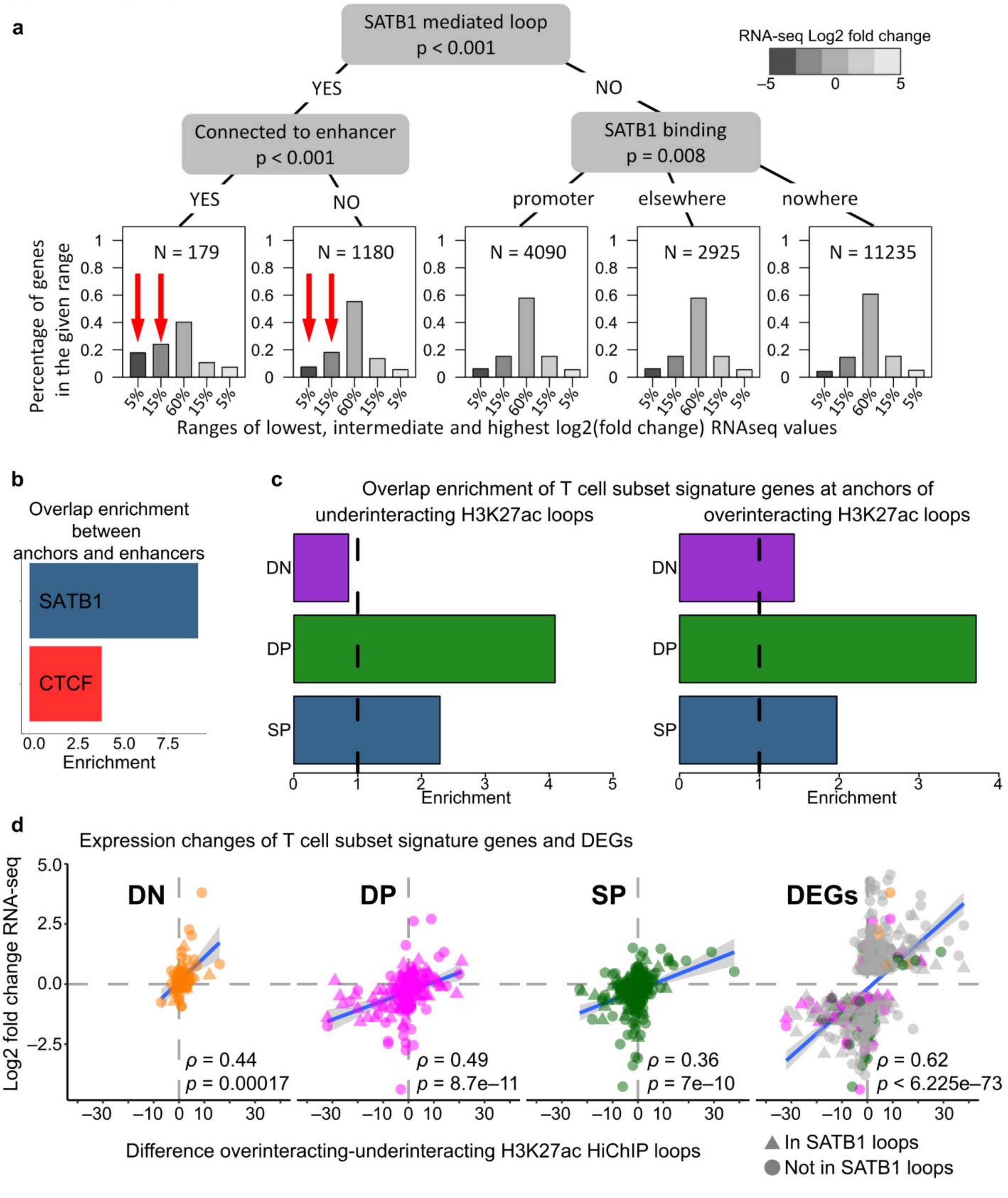

Supplementary Fig. 7. **SATB1-dependent promoter-enhancer chromatin loops in developing T cells. a** Conditional inference tree systematically probing all options of SATB1 binding and looping and their impact on gene expression. The x-axis indicates ranges of log2 fold change RNA-seq values. The y-axis represents a proportion of genes meeting a condition specified in the tree belonging to the particular range of deregulated genes specified on the x-axis. For example, ~20% of genes (y-axis) found in anchors of SATB1-dependent loops connected to an enhancer (first red arrow) belong to the range of 5% of the most underexpressed genes in *Satb1* cKO. The red arrows

highlight the disruption of the normal distribution of differentially expressed genes in the respective ranges that is present in SATB1-dependent loops. Differentially expressed genes that do not overlap with anchors of SATB1 loops display a normal distribution – reflecting the respective log2 fold change ranges (the three rightmost graphs). **b** Overlap enrichment between loop anchors and enhancers, presented separately for SATB1- and CTCF-dependent loops. **c** Overlap enrichment of T cell subset signature genes at anchors of H3K27ac under- and over-interacting loop anchors. Note the high enrichment of DP signature genes for both categories. Overlapping genes are depicted in the Source Data File. **d** Scatter plots indicating positive correlation (Spearman's ρ) between changes in RNA levels based on RNA-seq and differential H3K27ac looping between WT and *Satb1* cKO. Three leftmost graphs show correlation for DN, DP and SP T cell subset signature genes (described in Supplementary Fig. 4a). The rightmost graph shows that the correlation is highest when only significantly differentially expressed genes (DEGs) are selected. Note that in DP subset, the majority of genes is located in the bottom left quadrant and present in SATB1-dependent loops (triangles). P values and correlation coefficients were calculated using a function stat_cor from the R package ggpubr with default settings. The grey zones indicate 95% confidence level interval for predictions from a linear model (blue line).

Supplementary Figure 8

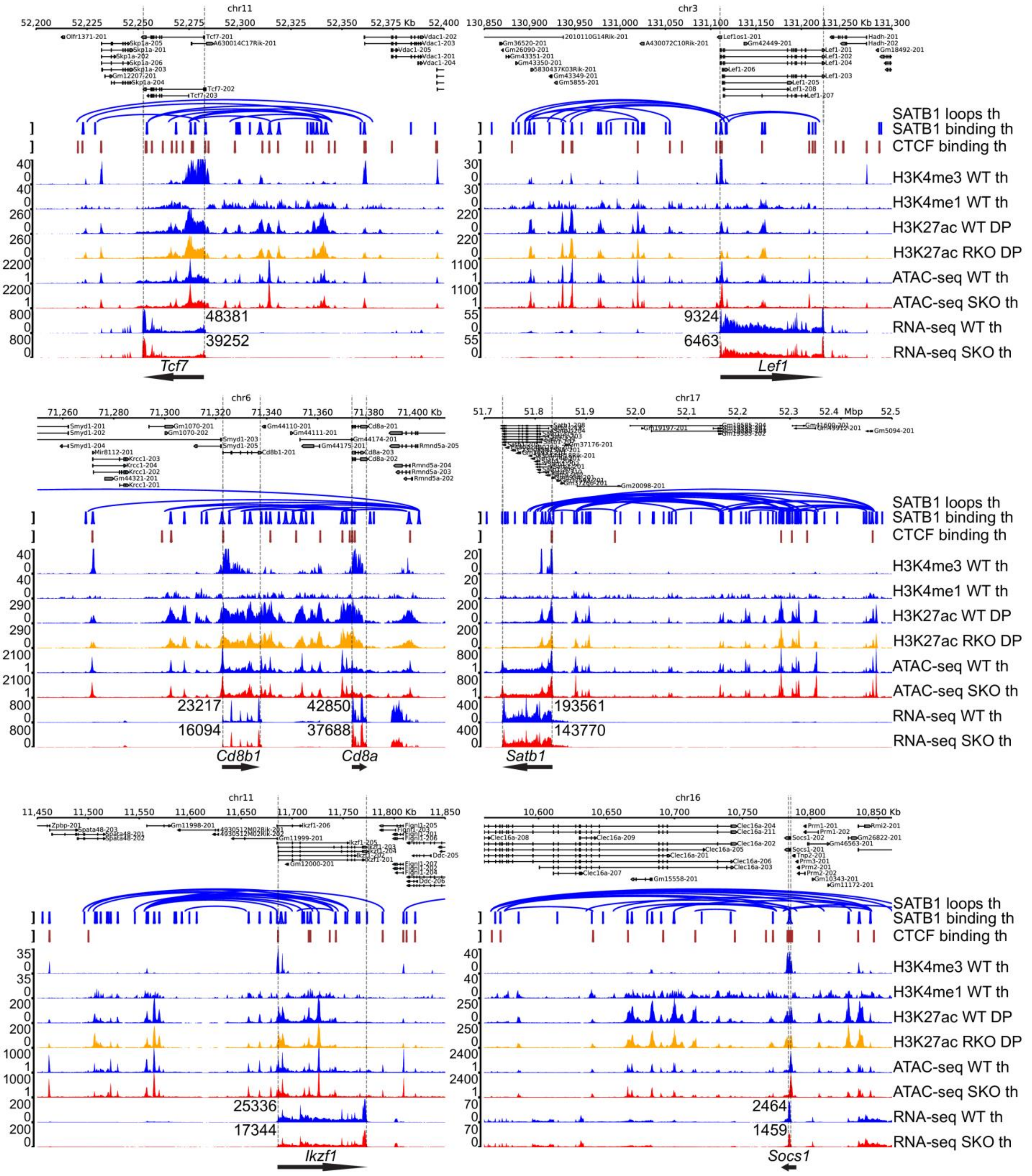

Supplementary Fig. 8. **Genomic tracks and SATB1 HiChIP loops for important immune-related genes.** Depicted genes displayed strong SATB1 looping connecting them with enhancers. Numbers next to gene regions within the RNA-seq tracks represent DESeq2 normalized RNA-seq counts. Genes related to cellular adhesion and communication *Cd28*, *Ccr7* and *Tnf* locus (including *Lta*, *Ltb*) are presented in Supplementary Fig. 12. Legend: th – thymocytes, DP – $CD4^{+}CD8^{+}$ T cells, RKO – *Rad21*$^{fl/fl}$*Cd4*-Cre^{+} and SKO – *Satb1*$^{fl/fl}$*Cd4*-Cre^{+}.

Supplementary Figure 9

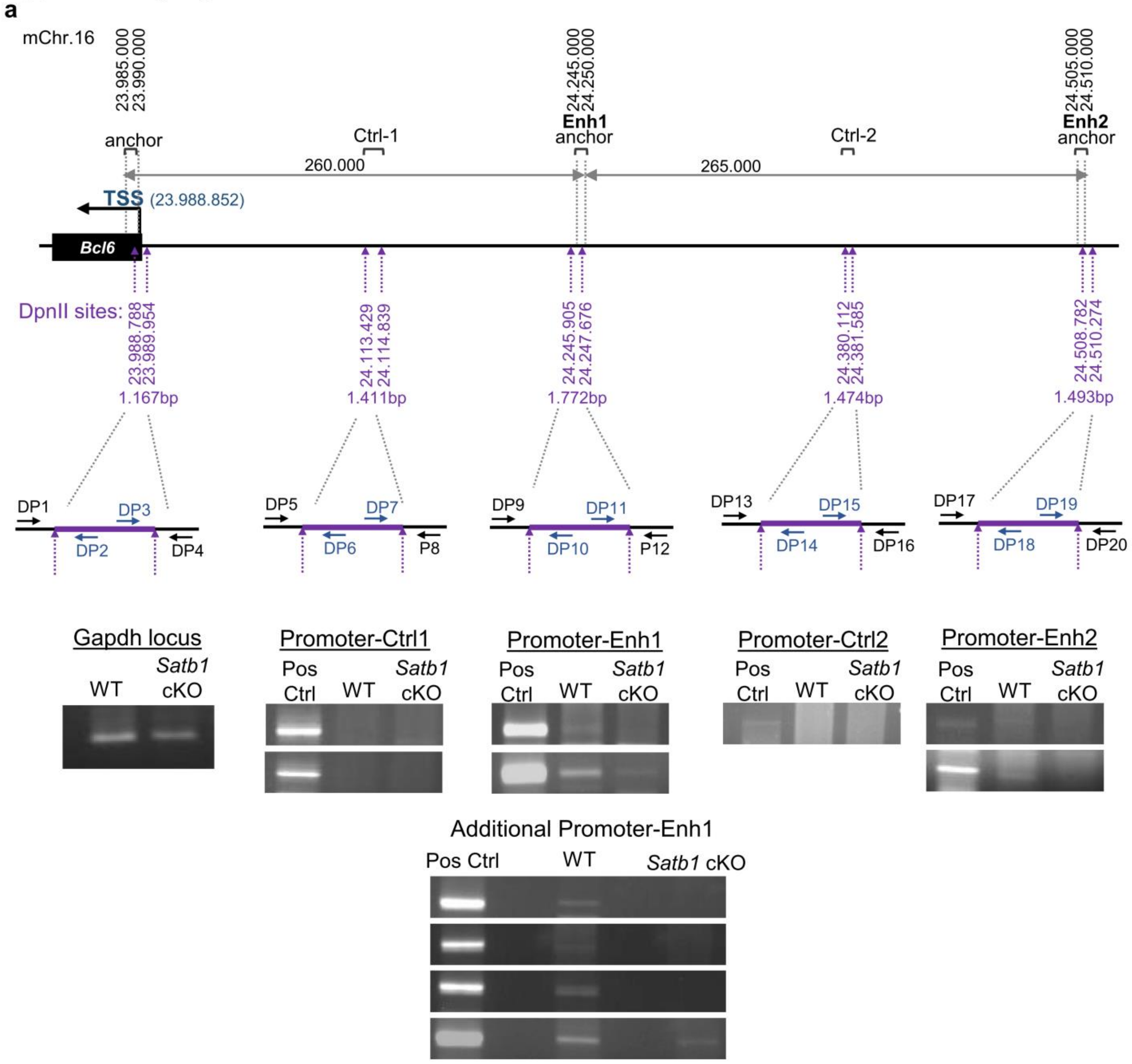

Primer pair	Interaction	PCR product size (bp)
**DP3 + DP7**	Bcl6 prom – Ctrl1	414
**DP3 + DP11**	Bcl6 prom – Enh1	190
**DP3 + DP15**	Bcl6 prom – Ctrl2	746
**DP3 + DP19**	Bcl6 prom – Enh2	241
**GAPDH**	*Gapdh* gene fragments	158

Supplementary Fig. 9. **Chromosome conformation capture at *Bcl6* locus. a** A scheme of the Chromosome Conformation Capture (3C) for the mouse *Bcl6* gene locus (525 kb). Coordinates (mm10) of the *DpnII* restriction fragments used in the 3C analysis assay are indicated for the *Bcl6* promoter region, its two enhancers and the genomic regions utilised as controls. The arrows indicate the *DpnII* recognition sites. For each fragment of interest, a pair of primers was designed and used for PCR reactions in pairwise combinations. *Gapd* products obtained with primer pairs for two genomic fragments of the *Gapd* locus, being 559 bp apart, were used as control. The 3C analysis has

been performed two times with three technical replicates for each biological replicate and similar results have been obtained.

Supplementary Figure 10

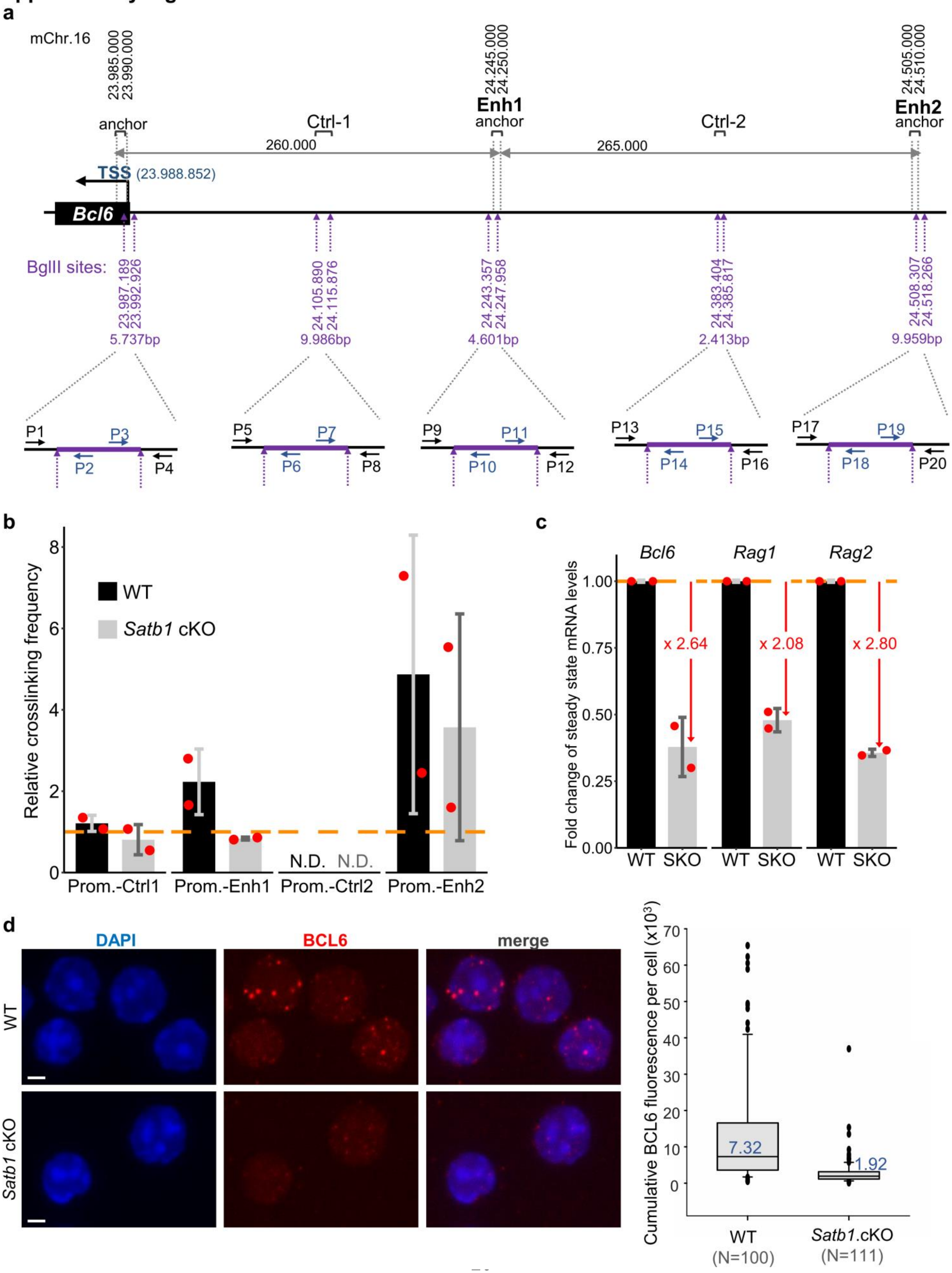

Supplementary Fig. 10. **SATB1 regulates *Bcl6* expression via promoter-enhancer loops. a** A scheme of the Chromosome Conformation Capture (3C) for the mouse *Bcl6* gene locus (525 kb). Coordinates (mm10) of the *BglII* restriction fragments used in the 3C analysis assay are indicated for the *Bcl6* promoter region, its two enhancers and the genomic regions utilised as controls. The arrows indicate the *BglII* recognition sites. For each fragment of interest, a pair of primers was designed and used for PCR reactions in paired combinations. The signal of each reaction was measured to calculate the relative crosslinking frequency. *Gapd* products obtained with primer pairs for two *BglII* genomic fragments of the *Gapd* locus, being 559 bp apart, were used for normalization. **b** Results of the 3C experiments depicted in **a**. The relative crosslinking frequency (y axis) was calculated for the *Bcl6* promoter *BglII* fragment with the two control (Ctrl-1 and Ctrl-2) fragments as well as the two upstream enhancers (Enh1 and Enh2). Results are from 2 technical replicates from 1 biological replicate to support data in Supplementary Fig. 9 and data from Hi-C and HiChIP experiments. Data represent the mean ± s.d. **c** Relative steady state mRNA levels in WT and *Satb1* cKO (SKO) thymocytes. RT-qPCR results for the mouse *Bcl6*, *Rag1* and *Rag2* genes. Results are from 2 biological replicates and the qPCR was performed in triplicates. The relative mRNA expression level for each gene was corrected based on *Hprt1* expression in WT and *Satb1* cKO thymocytes samples. Values were calculated based on the ΔΔCt method and they are presented as fold change in *Satb1* cKO (SKO) over WT. Data represent the mean ± s.d. **d** Immunofluorescence experiments on WT and *Satb1* cKO thymocytes for BCL6 expression. Blue DAPI, scale bar 2 μm. RIGHT: The cumulative fluorescence per cell values (y-axis) for each genotype (wild type and *Satb1* cKO) are characterized by box plots. The boundary of the box closest to zero indicates the 25th percentile, the line within the box marks the median, and the boundary of the box farthest from zero indicates the 75th percentile. Whiskers (error bars) above and below the box indicate the 90th and 10th percentiles. Single cell outliers (5% of total signals) are indicated in the vicinity outside the whiskers. Box plots were created in Sigma Plot 12.

Supplementary Figure 11

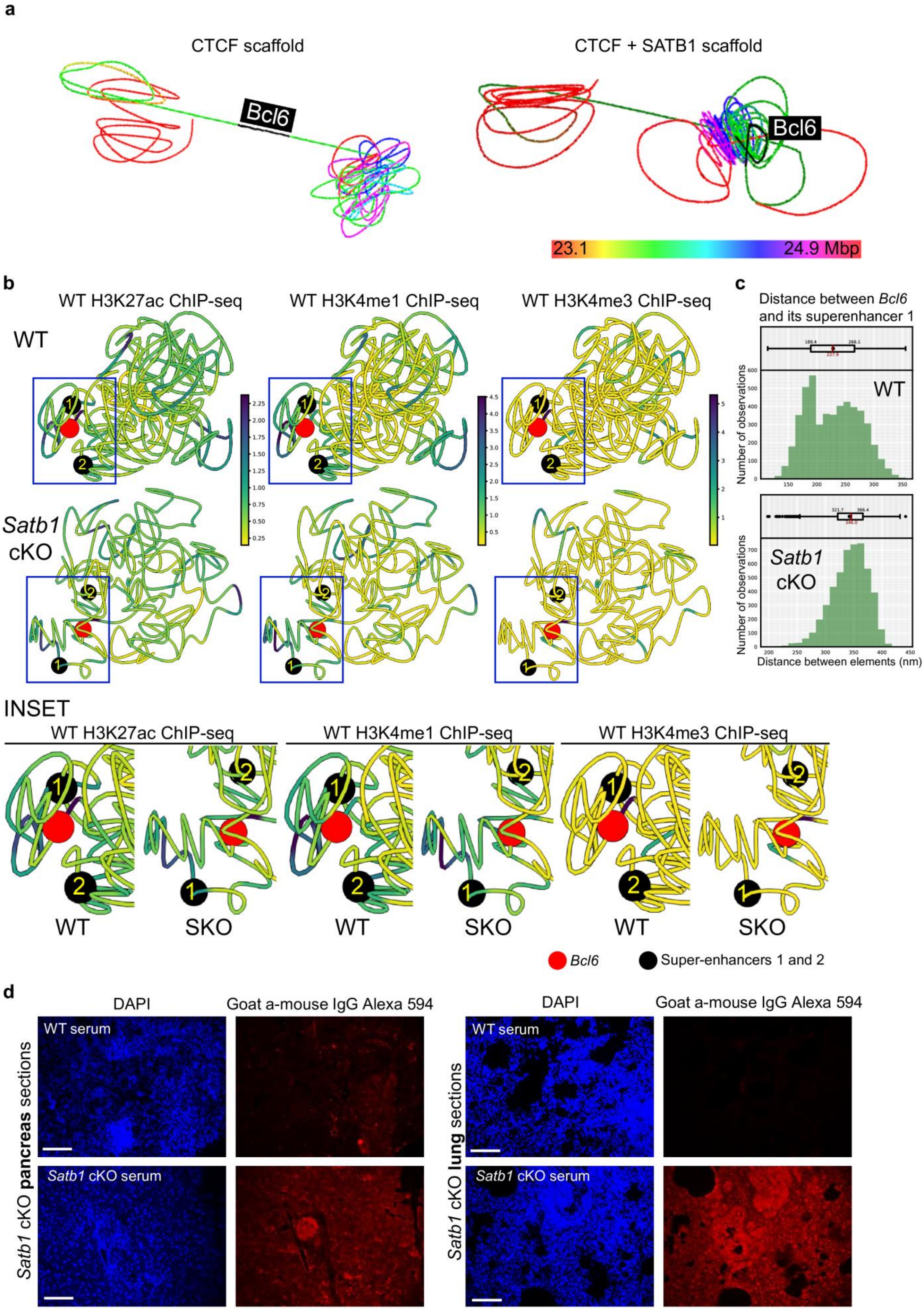

Supplementary Fig. 11. **Deregulation of Bcl6 promoter-enhancer contacts results in autoantibody production. a** 3D computational modeling of the *Bcl6* locus utilizing chromatin contact domains (CCD) from HiChIP data. The model indicated that CTCF is responsible for maintaining the high order structure which is not sufficient to mediate the contact between the *Bcl6* gene and its enhancers. The combination of CTCF and SATB1-based models emphasized the importance of SATB1 in mediating the promoter-enhancer contacts. **b** Hi-C-derived 3D models for WT and *Satb1* cKO thymocytes with overlaid WT ChIP-seq data for histone modifications H3K27ac, H3K4me1 and H3K4me3 (visualized as a color-gradient). Models indicate how active enhancers decorated by H3K27ac and H3K4me1 are located in a close spatial proximity to *Bcl6* gene via SATB1-dependent chromatin interactions in WT and not in *Satb1* cKO cells. WT ChIP-seq data were used to emphasize the importance of the 3D organization. 1D H3K27ac ChIP-seq peaks derived from HiChIP experiments available for WT and *Satb1* cKO did not reveal any major differences between the genotypes, further reinforcing the importance of SATB1-dependent 3D chromatin organization regulating *Bcl6* expression. Position of beads corresponding to SATB1 loop anchors and demarcating the super-enhancer regions were: chr16:23985000-23990000 (*Bcl6*), chr16:24245000-24250000 (SE1) and chr16:24505000-24510000 (SE2). **c** Distances between *Bcl6* and its super-enhancer 1 were quantified based on n=5,000 models derived from n=2 biological replicates of WT and *Satb1* cKO Hi-C datasets. The boxplots in the upper panel show median (red line) and mean (black dot) with the left and right edges of the box representing the 25th (Q1) and 75th (Q3) percentiles, respectively. The whiskers represent positions of the lowest data point above Q1-1.5*(Q3-Q1) and the highest data point below Q3+1.5*(Q3-Q1). Outliers outside the whiskers are shown as dots. All the distances for each model analyzed can be found in the source data file. Mean rank WT: 2616.3162. Mean rank *Satb1* cKO: 7384.6838, $P = 0.0$ (two-sided Mann-Whitney U test, non-adjusted for multiple comparisons), Dip test results: WT: [171.205, 200.087], [200.248, 298.606] and *Satb1* cKO: [198.063, 441.868]. **d** *Satb1* cKO pancreas and lung sections were incubated with serum from either WT or *Satb1* cKO animals to detect the presence of autoantibodies. Scale bar 100 μm. Two biological replicates have been performed for the pancreas sections and two biological replicates for the lung sections and similar results have been obtained.

Supplementary Figure 12

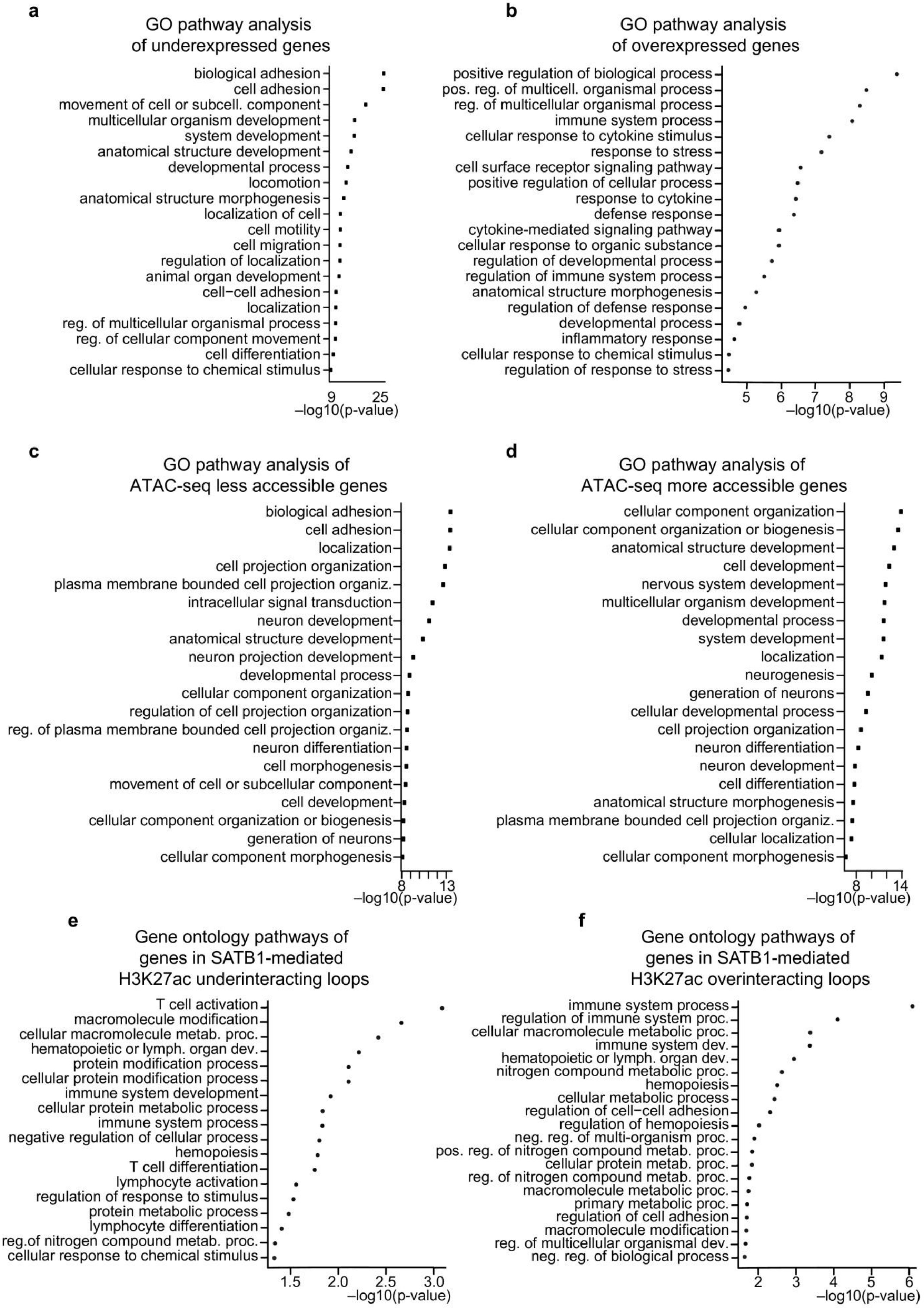

Supplementary Fig. 12. **Gene ontology pathways for the omics datasets used in this study.** **a** Gene ontology (GO) pathways of *Satb1* cKO underexpressed genes. **b** GO pathways of *Satb1* cKO overexpressed genes. **c** GO pathways of genes located in regions with less accessible chromatin in *Satb1* cKO. **d** GO pathways of genes located in regions with more accessible chromatin in *Satb1* cKO. **e** GO pathways of genes located in anchors of SATB1-dependent loops which were also H3K27ac underinteracting in *Satb1* cKO. **f** GO pathways of genes located in anchors of SATB1-dependent loops which are also H3K27ac overinteracting in *Satb1* cKO. In **a-f**, cumulative hypergeometric P values calculated by g:Profiler[17] are displayed.

Supplementary Figure 13

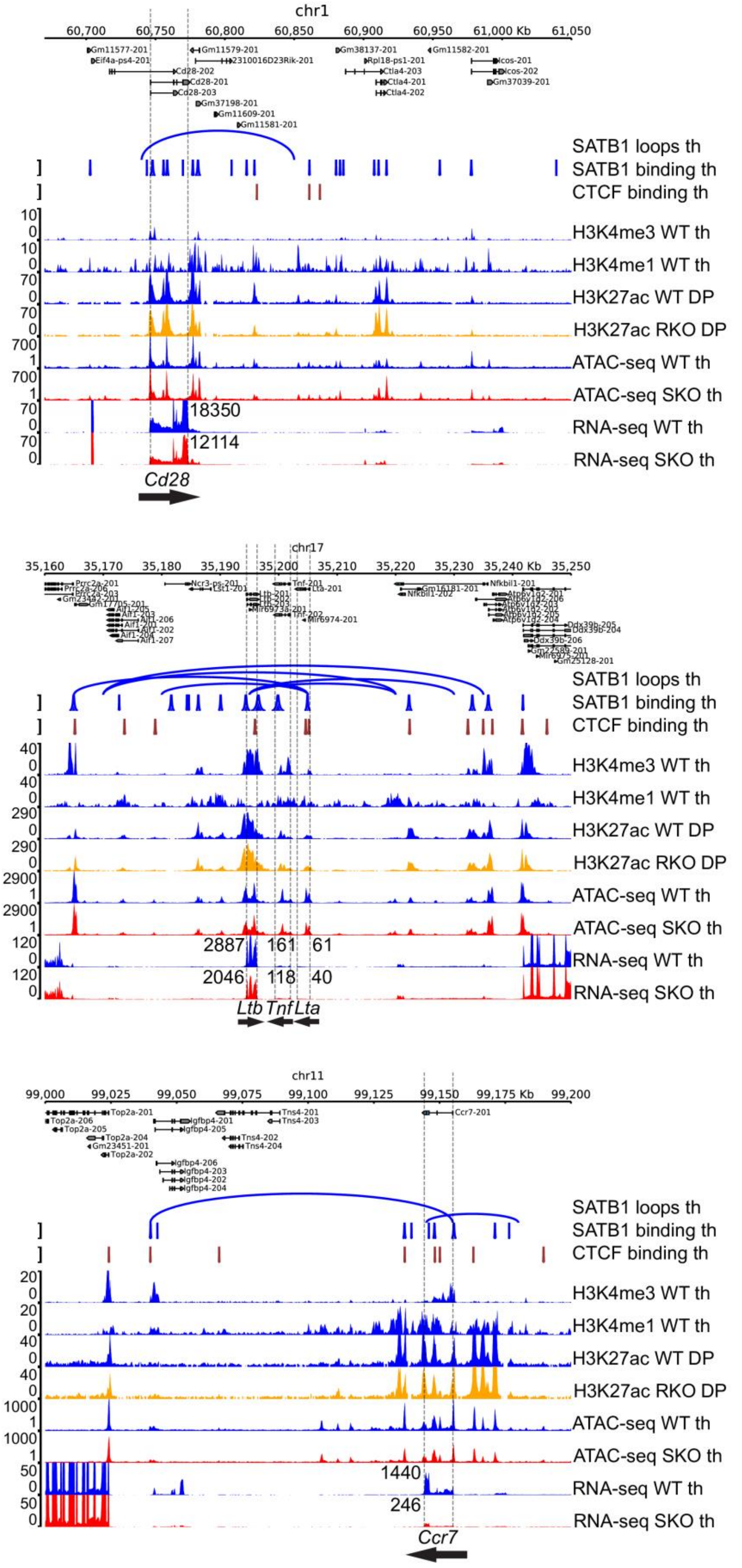

Supplementary Fig. 13. **Genomic tracks and SATB1 HiChIP loops for genes related to cellular adhesion and communication.** SATB1-dependent regulatory looping affects genes related to cellular adhesion and communication such as *Cd28*, *Ccr7* and *Tnf* locus (including *Lta*, *Ltb*). Numbers next to gene regions within the RNA-seq tracks represent DESeq2 normalized RNA-seq counts. Legend: th – thymocytes, DP – $CD4^{+}CD8^{+}$ T cells, RKO – *Rad21*$^{fl/fl}$*Cd4*-Cre^{+} and SKO – *Satb1*$^{fl/fl}$*Cd4*-Cre^{+}.

Supplementary Notes

Validation of long SATB1 isoform binding sites

To validate the long SATB1 isoform-specific binding sites that we have characterized, we compared them to two publicly available SATB1 ChIP-seq datasets that were prepared using standard SATB1 antibodies targeting non-selectively, both the short and long SATB1 isoforms[10,18]. Our dataset for the long SATB1 isoform binding sites had closer relative distance to both public SATB1 ChIP-seq datasets than expected by chance (Supplementary Fig. 4d). Irrespective of the differential peak size among the datasets, this method allowed us to validate a non-random overlap between the long and all SATB1 isoform datasets. Similarly, a pixel-based colocalization analysis based on super-resolution microscopy confirmed the significant correlation and overlap between the immunofluorescence staining utilizing antibodies targeting the long and all SATB1 isoforms[19]. Moreover, all three SATB1 binding site datasets showed qualitatively comparable association with genomic features (Supplementary Fig. 4e), collectively supporting the quality of our dataset. Ultimately, the validity of our dataset was also confirmed by the correlation between the peaks and peak-based chromatin loops and their functional deregulation in SATB1-depleted cells as discussed in the main text.

Results of the linear regression model

The quality plots of the model are depicted in Supplementary Fig. 6a-d and the adjusted R-square of the model was 0.1128. The change of the Akaike Information Criterion (AIC, Supplementary Fig. 6e) estimated how the quality of the model was affected when all the predictors were kept intact except one. The y-axis corresponds to the removed predictor, while the x-axis indicates how the AIC for the new model was altered. An increase indicated that the predictor was “useful” for the model. Based on the AIC plot, neither SATB1 binding upstream and downstream of genes nor CTCF-dependent loops contributed to the accuracy of the model. On the other hand, differences in chromatin accessibility and connectivity via H3K27ac loops along with SATB1-dependent loops performed very well as predictors. Non-useful

predictors were not used in the final model. The final model coefficients for the important predictors are displayed in Supplementary Fig. 6f. The sign of each coefficient indicates whether a predictor is associated with decreased (negative) or increased (positive) RNA levels in *Satb1* cKO. Genes present at anchors of overinteracting H3K27ac chromatin loops and/or with increased chromatin accessibility were associated with increased RNA levels in *Satb1* cKO. In contrast, the genes present at anchors of underinteracting H3K27ac chromatin loops or SATB1-dependent loops and/or genes bound by SATB1 were associated with reduced RNA levels.

Supplementary References

1. Denaxa, M. *et al.* Maturation-promoting activity of SATB1 in MGE-derived cortical interneurons. *Cell Rep.* **2**, 1351–1362 (2012).
2. Wolfer, A. *et al.* Inactivation of Notch1 in immature thymocytes does not perturb CD4 or CD8 T cell development. *Nat. Immunol.* **2**, 235–241 (2001).
3. Srinivas, S. *et al.* Cre reporter strains produced by targeted insertion of EYFP and ECFP into the ROSA26 locus. *BMC Dev. Biol.* **1**, 4 (2001).
4. Yoshida, H. *et al.* The *cis*-regulatory atlas of the mouse immune system. *Cell* **176**, 897-912.e20 (2019).
5. Cresswell, K. G. & Dozmorov, M. G. TADCompare: An R package for differential and temporal analysis of topologically associated domains. *Front. Genet.* **11**, (2020).
6. Seitan, V. C. *et al.* Cohesin-based chromatin interactions enable regulated gene expression within preexisting architectural compartments. *Genome Res.* **23**, 2066–2077 (2013).
7. Nora, E. P. *et al.* Targeted degradation of CTCF decouples local insulation of chromosome domains from genomic compartmentalization. *Cell* **169**, 930-944.e22 (2017).
8. Lieberman-Aiden, E. *et al.* Comprehensive mapping of long-range interactions reveals folding principles of the human genome. *Science* **326**, 289–293 (2009).
9. Heinz, S. *et al.* Simple combinations of lineage-determining transcription factors prime *cis*-regulatory elements required for macrophage and B cell identities. *Mol. Cell* **38**, 576–589 (2010).
10. Kitagawa, Y. *et al.* Guidance of regulatory T cell development by Satb1-dependent super-enhancer establishment. *Nat. Immunol.* **18**, 173–183 (2017).
11. Hao, B. *et al.* An anti-silencer– and SATB1-dependent chromatin hub regulates Rag1 and Rag2 gene expression during thymocyte development. *J. Exp. Med.* **212**, 809–824 (2015).
12. Favorov, A. *et al.* Exploring massive, genome scale datasets with the GenometriCorr package. *PLOS Comput. Biol.* **8**, e1002529 (2012).
13. Quinlan, A. R. & Hall, I. M. BEDTools: a flexible suite of utilities for comparing genomic features. *Bioinformatics* **26**, 841–842 (2010).
14. Rao, S. S. P. *et al.* A 3D map of the human genome at kilobase resolution reveals principles of chromatin looping. *Cell* **159**, 1665–1680 (2014).
15. Durand, N. C. *et al.* Juicer provides a one-click system for analyzing loop-resolution Hi-C experiments. *Cell Syst.* **3**, 95–98 (2016).
16. Rowley, M. J. *et al.* Analysis of Hi-C data using SIP effectively identifies loops in organisms from *C. elegans* to mammals. *Genome Res.* **30**, 447–458 (2020).
17. Reimand, J., Kull, M., Peterson, H., Hansen, J. & Vilo, J. g:Profiler–a web-based toolset for functional profiling of gene lists from large-scale experiments. *Nucleic Acids Res.* **35**, W193-200 (2007).
18. Hao, B. *et al.* An anti-silencer- and SATB1-dependent chromatin hub regulates *Rag1* and *Rag2* gene expression during thymocyte development. *J. Exp. Med.* **212**, 809–824 (2015).
19. Zelenka, T. *et al.* SATB1 undergoes isoform-specific phase transitions in T cells. *bioRxiv* 2021.08.11.455932 at https://doi.org/10.1101/2021.08.11.455932 (2022).
