# Supplementary material for: The 3D enhancer network of the developing T cell genome is shaped by SATB1
Source: Nat Commun. 2022 Nov 14;13:6954. doi: 10.1038/s41467-022-34345-y (PMC9663569; doi:10.1038/s41467-022-34345-y)
Supplement: Supplementary file 3 — Description of Additional Supplementary Files [file 41467_2022_34345_MOESM3_ESM.pdf]

<u>Descriptions of Additional Supplementary Files</u>

Supplementary Data 1: ChIP Atlas enrichment analysis. Output of the enrichment analysis of ChIP-Atlas, based on WT H3K27ac loop anchors from murine thymocytes and all available murine ChIP-seq datasets from blood cells for transcription factors.

Supplementary Data 2: RNA-seq results. Summarized data from DESeq2 differential analysis of stranded total RNA-seq experiment. Both gene and transcript levels are provided including the annotation dataset. Complete annotation and normalized RNA-seq values for individual replicates are provided in GEO repository under accession number GSE173470.

Supplementary Data 3: T cell subset signature genes. List of all selected DN, DP and SP T cell subset signature genes based on the Supplementary Fig. 4a. Output of the overlap enrichment analysis is also provided.

Supplementary Data 4: Hi-C and HiChIP statistics. Statistics summarizing the number of reads at different processing steps of Hi-C and HiChIP experiments.

Supplementary Data 5: Summary of HiChIP loops. Peak-to-all HiChIP loops for SATB1, CTCF and H3K27ac factors and their classification according to the presence of a thymus-specific enhancer and/or gene promoter (–5 kbp - TSS) in their loop anchors.

Supplementary Data 6 : Summary of SATB1 binding sites. SATB1 binding sites specific for the long protein isoform derived from HiChIP experiment with >60% Dangling End Pairs (~280 million reads).

Supplementary Data 7: Summary of ATAC-seq peaks. Merged ATAC-seq peaks identified in WT and Satb1 cKO thymocytes.

Supplementary Data 8: Differential H3K27ac HiChIP loops. Differential H3K27ac chromatin loops that are under- and over-interacting in Satb1 cKO, together with the list of genes found at anchors of the differential loops. Additional information such as RNA-seq differential expression values and information on SATB1 binding is provided.
