# Supplementary material for: The 3D enhancer network of the developing T cell genome is shaped by SATB1
Source: Nat Commun. 2022 Nov 14;13:6954. doi: 10.1038/s41467-022-34345-y (PMC9663569; doi:10.1038/s41467-022-34345-y)
Supplement: Supplementary file 12 — Supplementary Data 9 [file 41467_2022_34345_MOESM12_ESM.pdf]

FITC Annexin V

**Catalog# / Size**	640905 / 25 tests<br>640906 / 100 tests<br>640945 / 300 tests
**Regulatory Status**	RUO
**Other Names**	Annexin A5
**Description**	Annexin V (or Annexin A5) is a member of the annexin family of intracellular proteins that binds to phosphatidylserine (PS) in a calcium-dependent manner. PS is normally only found on the intracellular leaflet of the plasma membrane in healthy cells, but during early apoptosis, membrane asymmetry is lost and PS translocates to the external leaflet. Fluorochrome-labeled Annexin V can then be used to specifically target and identify apoptotic cells. Annexin V Binding Buffer (cat. no. 422201) is recommended for use with Annexin V staining.Annexin V binding alone cannot differentiate between apoptotic cells and necrotic. Therefore, we recommend using our Helix NP™ Blue (Cat. No. 425305), Helix NP™ Green (Cat. No. 425303) or Helix NP™ NIR (Cat. No. 425301). Early apoptotic cells will exclude 7-AAD and PI, while late stage apoptotic cells and necrotic cells will stain positively, due to the passage of these dyes into the nucleus where they bind to DNA.

Product Details

**Verified Reactivity**	All mammalian species
**Formulation**	Phosphate-buffered solution, pH 7.2, containing 0.09% sodium azide and BSA (origin USA)
**Preparation**	The purified protein was conjugated with FITC under optimal conditions.
**Concentration**	Lot-specific (to obtain lot-specific concentration, please enter the lot number in our Concentration and Expiration Lookup or Certificate of Analysis online tools.)
**Storage & Handling**	The Annexin V solution should be stored undiluted between 2°C and 8°C, and protected from prolonged exposure to light. Do not freeze.
**Application**	*FC - Quality tested*
**Recommended Usage**	Each lot of this product is quality control tested by immunofluorescent staining with flow cytometric analysis. For flow cytometric staining, the suggested use of this reagent is 5 µl per 100,000 - million cells in a 100 µl volume of Annexin V Binding Buffer (Cat No. 422201). It is recommended that the reagent be titrated for optimal performance for each application.
**Excitation Laser**	Blue Laser (488 nm)

Application Notes

Annexin V Staining

1. Wash cells twice with cold BioLegend cell staining buffer (Cat. No. 420201) and then resuspend cells in Annexin V Binding Buffer (Cat. No. 422201) at a concentration of $1x10^6$ cells/mL.
2. Transfer 100 µL of cell suspension in 5 ml test tube.
3. Add 5 µL of FITC Annexin V.
4. Add 10 µL of PI solution (Cat. No. 421301) or 7-AAD (Cat. No. 420403/420404).
5. Gently vortex the cells and incubate for 15 min at RT (25°C) in the dark.
6. Add 400 µL of Annexin V Binding Buffer (Cat. No. 422201) to each tube. Analyze by flow cytometry.

For a better experience detecting apoptosis, we now recommend Apotracker™. Cell staining with Apotracker™ is Calcium independent. Thus, no special buffers are required, and the protocol can be shortened for single-step co-staining with other reagents.

Application References

1. Koopman G, et al. 1994. *Blood* 84:1415.
2. Vermes I, et al. 1995. *J. Immunol. Methods* 184:39.
3. Dachary-Prigent J, et al. 1993. *Blood* 81:2554.
4. Sekine C, *et al.* 2009. *Int Immunol.* PubMed
5. Grujic M, *et al.* 2010. *J. Immunol.* 185:1730. PubMed
6. Mulik RS, *et al.* 2010. *Int J Pharm.* 398:190. PubMed
7. Gupta A, *et al.* 2011. *Leuk Res.* 35:1498. PubMed

8. Speth C,*et al.* 2013. *J Infect Dis.* 207:823. PubMed
9. Wang Q, *et al.* 2013. *Genes Dev.* 27:615. PubMed
10. Gill K, *et al.* 2013. *Biocheim Biophys Acta.* 830:2763. PubMed
11. Juel HB, *et al.* 2013. *PLoS One.* 8:64619. PubMed
12. Hellmann J, *et al.* 2013. *J. Immunol.* 191:1383. PubMed
13. Sequier S, *et al.* 2013. *PLoS One.* 8:70937. PubMed
14. Galski H, *et al.* 2013. *Biochem Pharmacol.* 86:584. PubMed
15. Chen YL, *et al.* 2013. *J Exp Med.* PubMed
16. Lin WH, *et al.* 2014. *PLoS One.* 9:83160. PubMed
17. Salge-Bartles U, 2014. *Thromb res.* 133:244. PubMed
18. Zhang X, *et al.* 2014. *Acta Biochim Biophys Sin.* 46:261. PubMed
19. Intermann J, *et al.* 2014. *Cell Signal.* 26:1567. PubMed
20. de Vires M, 2014. *Am J Physiol Lung Cell Mol Physiol.* 307:240. PubMed
21. M Parrula MC, *et al.* 2014. *Virus Res.* 189:206. PubMed
22. Passaro C, *et al.* 2014. *Mol Oncol.* 9:78. PubMed
23. Tsou WI, *et al.* 2014. *J Biol Chem.* 289:25750. PubMed
24. Awojoodu AO, *et al.* 2014. *Blood.* 124:1941. PubMed
25. Xiong R, *et al.* 2014. *Toxicol Appl Pharmacol.* 280:285. PubMed
26. Liu J, *et al.* 2015. *J Mol Cell Cardiol.* 80:10. PubMed

Product Citations

1. Siddhartha V, *et al.* 2017. Artif Cells Nanomed Biotechnol . 10.1080/21691401.2017.1313267. PubMed
2. Beeravolu N, McKee C, Chaudhry G 2017. J App Toxicol. 10.1002/jat.3469. PubMed
3. Zheng Q, *et al.* 2017. Mol Oncol. 10.1002/1878-0261.12056. PubMed
4. Karavasili C, *et al.* 2017. International Journal of Pharmaceutics. 10.1016/j.ijpharm.2017.05.061. PubMed
5. Wang M, *et al.* 2017. Biochemical and Biophysical Research Communications. 10.1016/j.bbrc.2017.05.097. PubMed
6. Chiani M, *et al.* 2017. Artif Cells Nanomed Biotechnol. 10.1080/21691401.2017.1337029. PubMed
7. Hafez A, *et al.* 2017. Oncogenesis. 10.1038/oncsis.2017.46. PubMed
8. Ishidome T *et al.* 2017. EBioMedicine. 22:89-99 . PubMed
9. Liu Y, *et al.* 2017. Acta Biomater. 10.1016/j.actbio.2017.08.021. PubMed
10. Kamiyama N, *et al.* 2017. Antiviral Res. 10.1016/j.antiviral.2017.08.007. PubMed
11. Thomas A, *et al.* 2017. PLoS One.. 10.1371/journal.pone.0183542. PubMed
12. Espinosa-Cueto P, *et al.* 2017. PLoS One.. 10.1371/journal.pone.0182126. PubMed
13. Zervou S, *et al.* 2017. PLoS One.. 10.1371/journal.pone.0182994. PubMed
14. Naqvi N,*et al.* 2017. Sci Rep.. 10.1038/s41598-017-12753-1. PubMed
15. Kuntz E, *et al.* 2017. Nat Med. 10.1038/nm.4399. PubMed
16. Ki H, *et al.* 2017. Biomed Pharmacother. 10.1016/j.biopha.2017.09.118. PubMed
17. Schuler F, *et al.* 2017. Nat Commun. . 10.1038/s41467-017-01850-4. PubMed
18. Thanuthanakhun N, *et al.* 2017. PLoS One. . 10.1371/journal.pone.0187610. PubMed
19. Lu Y, *et al.* 2018. Cancer Cell. 33:1048. PubMed
20. Yamauchi T *et al.* 2018. Cancer cell. 33(3):386-400 . PubMed
21. Greenblatt SM, *et al.* 2018. Cancer Cell. 33:1111. PubMed
22. Thompson PJ *et al.* 2019. Cell metabolism. 29(5):1045-1060 . PubMed
23. Ning X, *et al.* 2019. Mol Cell. 74:19. PubMed
24. Ni F, *et al.* 2019. Cell Stem Cell. 24:608. PubMed
25. Litzenburger UM, *et al.* 2017. Genome Biol. 18:15. PubMed
26. Noguchi F, *et al.* 2017. Mol Cancer Ther. 1.316666667. PubMed
27. Rauzan M, *et al.* 2017. PLoS One. 12:e0174107. PubMed
28. He J, *et al.* 2017. FASEB J. 31:2893. PubMed
29. Rajavel T, *et al.* 2017. Sci Rep. 2.665277778. PubMed
30. Pirali T, *et al.* 2017. ChemMedChem. 1.570833333. PubMed
31. Feng W, *et al.* 2017. Nat Commun. 0.697916667. PubMed
32. Venter C, *et al.* 2017. Cell Physiol Biochem. 43:1288. PubMed
33. Montero–Herradón S, *et al.* 2017. Organogenesis. 0.675. PubMed
34. Lee HW, *et al.* 2018. Am J Physiol Renal Physiol. 314:F280. PubMed
35. Lopes–Coelho F, *et al.* 2017. Oncotarget. 8:82803. PubMed
36. Lim JA, *et al.* 2018. BMC Cancer. 0.860416667. PubMed
37. Bouhaddou M, *et al.* 2018. PLoS Comput Biol. 14:e1005985. PubMed
38. Jones PD, *et al.* 2018. Arterioscler Thromb Vasc Biol. 38:1711. PubMed
39. Nunes SC, *et al.* 2018. Sci Rep. 6.939583333. PubMed
40. Schleicher EM, *et al.* 2018. Nucleic Acids Res. 46:8908. PubMed
41. Clements KE, *et al.* 2018. Nucleic Acids Res. 46:8898. PubMed
42. Zhao CC, *et al.* 2018. Oncol Rep. 40:3392. PubMed
43. Yao Z, *et al.* 2018. J Virol. 92:00:00. PubMed
44. Kumar S, *et al.* 2018. Cancers (Basel). 0.724305556. PubMed
45. Sharif T, *et al.* 2018. Autophagy. 1.101388889. PubMed
46. Eichelmann AK, *et al.* 2018. Sci Rep. 8:17553. PubMed
47. Santos LC, *et al.* 2019. Nat Commun. 1.328472222. PubMed
48. Sales–Dias J, *et al.* 2019. PLoS One. 14:e0217002. PubMed
49. Zheng M, *et al.* 2019. Proc Natl Acad Sci U S A. 116:12422. PubMed
50. Kumar S, *et al.* 2019. Int J Cancer. 146:1652. PubMed
51. Vancsik T, *et al.* 2019. Cancer Med. 3.313888889. PubMed
52. Lyu X, *et al.* 2019. BMC Genomics. 1.235416667. PubMed
53. Esteghamat F, *et al.* 2019. Nat Genet. 51:1233. PubMed
54. Fang P, *et al.* 2019. Commun Biol. 0.315972222. PubMed

55. Mendès C, *et al.* 2019. Sci Rep. 9:14107. PubMed
56. Santos I, *et al.* 2019. Nutrients. 2.210416667. PubMed
57. Blümel E, *et al.* 2019. Oncoimmunology. 8:e1641387. PubMed
58. Young MM, *et al.* 2019. Cell Death Dis. 1.004861111. PubMed
59. Di Somma S, *et al.* 2019. Cancers (Basel). 1.70625. PubMed
60. Matsumoto A, *et al.* 2019. J Extracell Vesicles. 9:1696517. PubMed
61. Jing Y, *et al.* 2019. J Allergy Clin Immunol. 144:1377. PubMed
62. Gladow N, *et al.* 2020. PLoS One. 15:e0227734. PubMed
63. Quan M, *et al.* 2019. Oncol Rep. 41:3051. PubMed
64. Fortin J, *et al.* 2020. Cancer Cell. 37:308. PubMed
65. Chen MS, *et al.* 2019. Mol Cancer Res. 17:697. PubMed
66. Blümel E, *et al.* 2020. Oncoimmunology. 9:1751561. PubMed
67. Labi V, *et al.* 2019. Genes Dev. 33:1673. PubMed
68. Bowen ME, *et al.* 2019. Dev Cell. 50:212. PubMed
69. Uriostegui–Arcos M, *et al.* 2020. Open Biol. 10:200050. PubMed
70. Liu H, *et al.* 2020. Cell Death Dis. 0.767361111. PubMed
71. Sekine C, *et al.* 2009. Int Immunol. 1.079861111. PubMed
72. Grujic M, *et al.* 2010. J Immunol. 185:1730. PubMed
73. Putheti P, *et al.* 2010. PLoS One. 5:e8706. PubMed
74. Speth C, *et al.* 2013. J Infect Dis. 207:823. PubMed
75. Wang Q, *et al.* 2013. Genes Dev. 27:615. PubMed
76. Gill K, *et al.* 2013. Biochim Biophys Acta. 1830:2763. PubMed
77. Juel H, *et al.* 2013. PLoS One. 8:64619. PubMed
78. Gupta A, *et al.* 2011. Leuk Res. 35:1498. PubMed
79. Mulik R, *et al.* 2010. Int J Pharm. 398:190. PubMed
80. Hellmann J, *et al.* 2013. J Immunol. 191:1383. PubMed
81. Séguier S, *et al.* 2013. PLoS One. 8:70937. PubMed
82. Passaro C, *et al.* 2013. Endocr Relat Cancer. 20:633. PubMed
83. Galski H, *et al.* 2013. Biochem Pharmacol. 86:584. PubMed
84. Chen Y, *et al.* 2013. J Exp Med. 210:2515. PubMed
85. Lin W, *et al.* 2014. PLoS One. 9:83160. PubMed
86. Salge-Bartels U, *et al.* 2014. Thromb Res. 133:244. PubMed
87. Zhang X, *et al.* 2014. Acta Biochim Biophys Sin. 46:261. PubMed
88. Intemann J, *et al.* 2014. Cell Signal. 26:1567. PubMed
89. Vries M, *et al.* 2014. Am J Physiol Lung Cell Mol Physiol. 307:240. PubMed
90. Parrula M, *et al.* 2014. Virus Res. 189:206. PubMed
91. Passaro C, *et al.* 2014. Mol Oncol. 9:78. PubMed
92. Tsou W, *et al.* 2014. J Biol Chem. 289:25750. PubMed
93. Awojoodu A, *et al.* 2014. Blood. 124:1941. PubMed
94. Xiong R, *et al.* 2014. Toxicol Appl Pharmacol. 280:285. PubMed
95. Liu J, *et al.* 2015. J Mol Cell Cardiol. 80:10. PubMed
96. Lhuillier C, *et al.* 2015. J Biol Chem. 290: 16797 - 16811. PubMed
97. CO L, *et al.* 2015. Nat Commun. 6: 7739. PubMed
98. Brigger D, *et al.* 2015. Cell Death Dis. 6: e1861. PubMed
99. Fujii T, *et al.* 2015. Proc Natl Acad Sci U S A. 112: 12800 - 12805. PubMed
100. Xiong R, *et al.* 2015. Mol Pharmacol. 88: 1045 - 1054. PubMed
101. Polak R, *et al.* 2015. Blood. 126: 2404 - 2414. PubMed
102. Slone E, *et al.* 2015. J Leukoc Biol. 98: 791 - 804. PubMed
103. Kumai T, *et al.* 2015. Sci Rep. 5:16280. PubMed
104. Carlsson J, *et al.* 2015. PLoS One. 10: 0143741. PubMed
105. Cardoso B, *et al.* 2015. PLoS One. 10: 0143897. PubMed
106. Ligtenberg M, *et al.* 2016. J Immunol. 196: 759 - 766. PubMed
107. Akane K, *et al.* 2016. Proc Natl Acad Sci U S A. 113: 2460 - 2465. PubMed
108. Derudder E, *et al.* 2016. Proc Natl Acad Sci U S A. 113: 5065-5070. PubMed
109. Gedye C, *et al.* 2016. Sci Rep. 6: 25220. PubMed
110. O'Neill K, *et al.* 2016. Genes Dev. 30: 973-988. PubMed
111. Kitayama J, *et al.* 2016. PLoS One. 11: 0154542. PubMed
112. Zhang J, *et al.* 2016. Cell Death Dis. 7:e2266. PubMed
113. Soprano M, *et al.* 2016. PLoS One. 11: 0155970. PubMed
114. Wang D, *et al.* 2016. J Cell Sci. 129: 2261 - 2272. PubMed
115. Langsch S, *et al.* 2016. Cancer Res . 76: 4160 - 4169. PubMed
116. Jones P, *et al.* 2016. J Biol Chem. 291: 16318 - 16327. PubMed
117. Pfalzgraff A, *et al.* 2016. Sci Rep. 6:31577. PubMed
118. Uchtenhagen H, *et al.* 2016. Nat Commun. 7:12614. PubMed
119. Chheda Z, *et al.* 2016. J Immunol. 197: 2016 - 2026. PubMed
120. Becker A, *et al.* 2016. Sci Rep. 6: 33847. PubMed
121. Hrdinka M, *et al.* 2016. PLoS One. 11: 0162863. PubMed
122. Geiger R, *et al.* 2016. Cell. 167:829-842. PubMed
123. Liu B, *et al.* 2016. J Biol Chem. 291: 23869 - 23881. PubMed
124. Yoon Y, *et al.* 2016. Stem Cell Reports. 7:840-853. PubMed
125. L Chang, Y Li, D Kaplan 2016. Sci Rep. 6:36862. PubMed
126. Douguet L, *et al.* 2016. PLoS One. 11:e0166164. PubMed
127. Toutounchian J, *et al.* 2016. Mol Pharmacol. 91(1):1-13. PubMed
128. Coutaz M, *et al.* 2016. Sci Rep. 6:39117. PubMed
129. Herold N, *et al.* 2017. Nat Med. 23:256-263. PubMed
130. Faria M, *et al.* 2017. PLoS One. 12(2):e0172689. PubMed
131. Clements KE, *et al.* 2020. Nat Commun. 4.706944444. PubMed
132. Schleicher EM, *et al.* 2020. PLoS Genet. 16:e1009176. PubMed
133. Simoni L, *et al.* 2020. Cell Rep. 33:108330. PubMed
134. Huang YL, *et al.* 2020. Elife. 9:00. PubMed

135. Dewdney B, *et al.* 2020. Sci Rep. 10:16769. PubMed
136. Alcon C, *et al.* 2020. Cell Death Dis. 0.898611111. PubMed
137. Cai Z, *et al.* 2020. Cell Rep. 31:107816. PubMed
138. Kang JH, *et al.* 2020. Cell Death Dis. 0.803472222. PubMed
139. Dhoonmoon A, *et al.* 2020. Nucleic Acids Res. 48:7252. PubMed
140. Ramalingam P, *et al.* 2020. J Exp Med. 217:00:00. PubMed
141. Kim EH, *et al.* 2020. Elife. 9:00. PubMed
142. Pajarillo E, *et al.* 2020. J Biol Chem. 295:3040. PubMed
143. Pahari S, *et al.* 2020. Autophagy. 1.375694444. PubMed
144. Zhang Y, *et al.* 2021. Cell Death Dis. 0.563194444. PubMed
145. Xie S, *et al.* 2021. Sci Rep. 0.660416667. PubMed

RRID

AB_2561291 (BioLegend Cat. No. 640905)
AB_2561292 (BioLegend Cat. No. 640906)
AB_2629519 (BioLegend Cat. No. 640945)

Antigen Details

Biology Area Apoptosis/Tumor Suppressors/Cell Death, Cell Biology, Neuroscience

Gene ID 308

Product Data

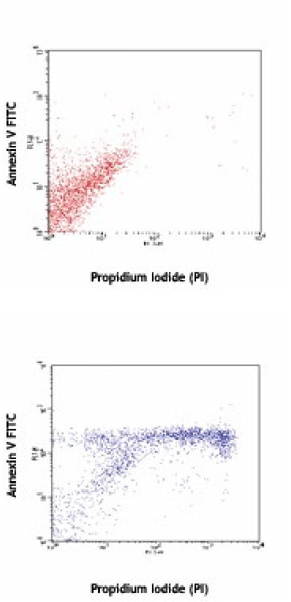

Human T leukemia cell line, Jurkat, non-treated (top) or treated (bottom) with BioLegend's anti-human CD95 (EOS9.1) mAb (cat. 305704) for 6 hours, then stained with Annexin V-FITC and Propidium Iodide (PI) (cat. 421301)

For research use only. Not for diagnostic use. Not for resale. BioLegend will not be held responsible for patent infringement or other violations that may occur with the use of our products.

*These products may be covered by one or more Limited Use Label Licenses (see the BioLegend Catalog or our website, www.biolegend.com/ordering#license). BioLegend products may not be transferred to third parties, resold, modified for resale, or used to manufacture commercial products, reverse engineer functionally similar materials, or to provide a service to third parties without written approval of BioLegend. By use of these products you accept the terms and conditions of all applicable Limited Use Label Licenses. Unless otherwise indicated, these products are for research use only and are not intended for human or animal diagnostic, therapeutic or commercial use.

BioLegend Inc., 8999 BioLegend Way, San Diego, CA 92121 www.biolegend.com
Toll-Free Phone: 1-877-Bio-Legend (246-5343) Phone: (858) 768-5800 Fax: (877) 455-9587

Technical Data Sheet

PE Rat Anti-Mouse CD4

Product Information

**Material Number:**	553730
**Alternate Name:**	Cd4; CD4 antigen; L3T4; Ly-4; T-cell surface antigen T4/Leu-3
**Size:**	0.2 mg
**Concentration:**	0.2 mg/ml
**Clone:**	GK1.5
**Immunogen:**	Mouse CTL clone V4
**Isotype:**	Rat (LEW) IgG2b, κ
**Reactivity:**	QC Testing: Mouse
**Storage Buffer:**	Aqueous buffered solution containing ≤0.09% sodium azide.

Description

The GK1.5 monoclonal antibody specifically binds to the mouse CD4 (L3T4) differentiation antigen. CD4 is expressed on most thymocytes, a subpopulation of mature T lymphocytes (i.e., MHC class II-restricted T cells, including most T helper cells), and a subset of NK-T cells. In addition, CD4 has also been reported to be detectable on pluripotent hematopoietic stem cells, bone marrow myeliod and B-lymphocyte precursors, intrathymic lymphoid precursors, and a subset of splenic dendritic cells. CD4 has also been reported to be expressed on the plasma membrane of mouse egg cells and is involved in adhesion of the egg to MHC class II-bearing sperm. CD4 is an antigen coreceptor on the T-cell surface which interacts with MHC class II molecules on antigen-presenting cells. It participates in T-cell activation through its association with the T-cell receptor complex and protein tyrosine kinase lck. GK1.5 mAb reportedly blocks binding of the RM4-5 (Cat. No. 553046/553047) and H129.19 (Cat. No. 553650/553651), but not RM4-4 (Cat. No. 553055) antibodies.

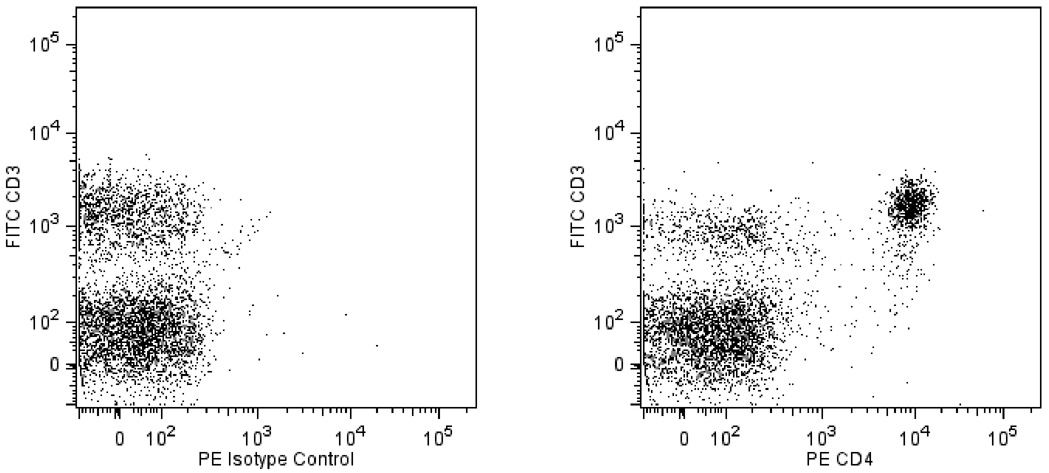

Multicolor flow cytometric analysis of CD4 expression on mouse splenocytes. *Splenic leukocytes were stained simultaneously with FITC Hamster Anti-Mouse CD3e antibody (Cat. No. 561829/553730/557308) and with either BD Pharmingen™ PE Rat IgG2a, κ Isotype Control (Cat. No. 553989; Left Panel) or BD Pharmingen™ PE Rat Anti-Mouse CD4 antibody (Cat. No. 553730; Right Panel). Two-color flow cytometric dot plots show the correlated expression patterns of CD4 (or Ig Isotype control staining) versus CD3 for gated events with the forward and side light-scatter characteristics of viable splenic leukocytes. Flow cytometry was performed using a BD™ LSR II Flow Cytometer System.*

Preparation and Storage

The monoclonal antibody was purified from tissue culture supernatant or ascites by affinity chromatography.
The antibody was conjugated with R-PE under optimum conditions, and unconjugated antibody and free PE were removed.
Store undiluted at 4°C and protected from prolonged exposure to light. Do not freeze.

Application Notes

Application

Flow cytometry	Routinely Tested

BD Biosciences
bdbiosciences.com

United States	Canada	Europe	Japan	Asia Pacific	Latin America/Caribbean
877.232.8995	800.979.9408	32.53.720.550	0120.8555.90	65.6861.0633	55.11.5185.9995

For country contact information, visit **bdbiosciences.com/contact**

Conditions: The information disclosed herein is not to be constructed as a recommendation to use the above product in violation of any patents. BD Biosciences will not be help responsible for patent infringement or other violations that may occur with the use of our products. Purchase does not include or carry any right to resell or transfer this product either as a stand-alone product or as a component of another product. Any use of this product other than the permitted use without the express written authorization of Becton, Dickinson and Company is sticly prohibited.
For Research Use Only. Not for use in diagnostic or therapeutic procedures. Not for resale.
Unless otherwise noted, BD, BD Logo and all other trademarks are property of Becton, Dickinson and Company. © 2011 BD

Suggested Companion Products

Catalog Number	Name	Size	Clone
553989	PE Rat IgG2b, κ Isotype Control	0.1 mg	A95-1
553062	FITC Hamster Anti-Mouse CD3e	0.5 mg	145-2C11
554656	Stain Buffer (FBS)	500 ml	(none)

Product Notices

1. Since applications vary, each investigator should titrate the reagent to obtain optimal results.
2. Please refer to www.bdbiosciences.com/pharmingen/protocols for technical protocols.
3. For fluorochrome spectra and suitable instrument settings, please refer to our Multicolor Flow Cytometry web page at www.bdbiosciences.com/colors.
4. Caution: Sodium azide yields highly toxic hydrazoic acid under acidic conditions. Dilute azide compounds in running water before discarding to avoid accumulation of potentially explosive deposits in plumbing.
5. An isotype control should be used at the same concentration as the antibody of interest.

References

Dialynas DP, Quan ZS, Wall KA, et al. Characterization of the murine T cell surface molecule, designated L3T4, identified by monoclonal antibody GK1.5: similarity of L3T4 to the human Leu-3/T4 molecule. *J Immunol.* 1983; 131(5):2445-2451. (Immunogen: Blocking, Depletion, Immunoprecipitation)

Dialynas DP, Wilde DB, Marrack P, et al. Characterization of the murine antigenic determinant, designated L3T4a, recognized by monoclonal antibody GK1.5: expression of L3T4a by functional T cell clones appears to correlate primarily with class II MHC antigen-reactivity. *Immunol Rev.* 1983; 74:29-56. (Clone-specific: Blocking, Depletion, Immunoprecipitation)

Frederickson GG, Basch RS. L3T4 antigen expression by hemopoietic precursor cells. *J Exp Med.* 1989; 169(4):1473-1478. (Biology)

Ghobrial RR, Boublik M, Winn HJ, Auchincloss H Jr. In vivo use of monoclonal antibodies against murine T cell antigens. *Clin Immunol Immunopathol.* 1989; 52(3):486-506. (Biology)

Guo MW, Watanabe T, Mori E, Mori T. Molecular structure and function of CD4 on murine egg plasma membrane. *Zygote.* 1995; 3(1):65-73. (Biology: Blocking)

Janeway CA Jr. The T cell receptor as a multicomponent signalling machine: CD4/CD8 coreceptors and CD45 in T cell activation. *Annu Rev Immunol.* 1992; 10:645-674. (Biology)

Wineman JP, Gilmore GL, Gritzmacher C, Torbett BE, Muller-Sieburg CE. CD4 is expressed on murine pluripotent hematopoietic stem cells. *Blood.* 1992; 180(7):1717-1724. (Biology)

BD Biosciences

bdbiosciences.com

United States	Canada	Europe	Japan	Asia Pacific	Latin America/Caribbean
877.232.8995	800.979.9408	32.53.720.550	0120.8555.90	65.6861.0633	55.11.5185.9995

For country contact information, visit **bdbiosciences.com/contact**

Conditions: The information disclosed herein is not to be constructed as a recommendation to use the above product in violation of any patents. BD Biosciences will not be help responsible for patent infringement or other violations that may occur with the use of our products. Purchase does not include or carry any right to resell or transfer this product either as a stand-alone product or as a component of another product. Any use of this product other than the permitted use without the express written authorization of Becton, Dickinson and Company is stictly prohibited.

For Research Use Only. Not for use in diagnostic or therapeutic procedures. Not for resale.

Unless otherwise noted, BD, BD Logo and all other trademarks are property of Becton, Dickinson and Company. © 2011 BD

Version: 1   Revision Date: 11/30/2012

FITC anti-mouse CD4 Antibody

**Catalog# / Size**	100405 / 50 µg<br>100406 / 500 µg
**Clone**	GK1.5
**Regulatory Status**	RUO
**Other Names**	L3T4, T4
**Isotype**	Rat IgG2b, κ
**Description**	CD4 is a 55 kD protein also known as L3T4 or T4. It is a member of the Ig superfamily, primarily expressed on most thymocytes, a subset of T cells, and weakly on macrophages and dendritic cells. It acts as a coreceptor with the TCR during T cell activation and thymic differentiation by binding MHC class II and associating with the protein tyrosin kinase, lck.

Product Details

**Verified Reactivity**	Mouse
**Antibody Type**	Monoclonal
**Host Species**	Rat
**Immunogen**	Mouse CTL clone V4
**Formulation**	Phosphate-buffered solution, pH 7.2, containing 0.09% sodium azide.
**Preparation**	The antibody was purified by affinity chromatography, and conjugated with FITC under optimal conditions.
**Concentration**	0.5 mg/ml
**Storage & Handling**	The CD4 antibody solution should be stored undiluted between 2°C and 8°C, and protected from prolonged exposure to light. **Do not freeze.**
**Application**	FC - Quality tested
**Recommended Usage**	Each lot of this antibody is quality control tested by immunofluorescent staining with flow cytometric analysis. For flow cytometric staining, the suggested use of this reagent is ≤0.25 µg per million cells in 100 µl volume. It is recommended that the reagent be titrated for optimal performance for each application.
**Excitation Laser**	Blue Laser (488 nm)
**Application Notes**	Additional reported applications (for the relevant formats) include: blocking of CD4^{+} T cell activation[1,4,11], thymocyte costimulation[3], *in vitro* and *in vivo* depletion[2,5-8], blocking of egg-sperm cell adhesion[1,4], immunohistochemical staining of acetone-fixed frozen sections[9,10], immunoprecipitation[1,2], and spatial biology (IBEX)[12,13]. The GK1.5 antibody is able to block CD4 mediated cell adhesion and T cell activation. Binding of GK1.5 antibody to CD4 T cells can be blocked by RM4-5 antibody, but not RM4-4 antibody. For *in vivo* studies or highly sensitive assays, we recommend Ultra-LEAF™ purified antibody (Cat. No. 100442) with a lower endotoxin limit than standard LEAF™ purified antibodies (Endotoxin < 0.01 EU/µg).

Application References

1. Dialynas DP, *et al.* 1983. *J. Immunol.* 131:2445. (Block, IP)
2. Dialynas DP, *et al.* 1983. *Immunol. Rev.* 74:29. (IP, Deplete)
3. Wu L, *et al.* 1991. *J. Exp. Med.* 174:1617. (Costim)
4. Godfrey DI, *et al.* 1994. *J. Immunol.* 152:4783. (Block)
5. Gavett SH, *et al.* 1994. *Am. J. Respir. Cell. Mol. Biol.* 10:587. (Deplete)
6. Schuyler M, *et al.* 1994. *Am. J. Respir. Crit. Care Med.* 149:1286. (Deplete)
7. Ghobrial RR, *et al.* 1989. *Clin. Immunol. Immunopathol.* 52:486. (Deplete)
8. Israelski DM, *et al.* 1989. *J. Immunol.* 142:954. (Deplete)
9. Zheng B, *et al.* 1996. *J. Exp. Med.* 184:1083. (IHC)
10. Frei K, *et al.* 1997. *J. Exp. Med.* 185:2177. (IHC)
11. Felix NJ, *et al.* 2007. *Nat. Immunol.* 8:388. (Block)

12. Radtke AJ, *et al.* 2020. *Proc Natl Acad Sci U S A.* 117:33455-65. (SB) PubMed
13. Radtke AJ, *et al.* 2022. *Nat Protoc.* 17:378-401. (SB) PubMed

Product Citations

1. Zongyi Y, *et al.* 2017. PLoS One. 10.1371/journal.pone.0189617. PubMed
2. Reynolds CJ, *et al.* 2018. Sci Rep. 8:672. PubMed
3. Nechama M, *et al.* 2018. Nat Commun. 9:1603. PubMed
4. Habib S, *et al.* 2018. Infect Immun. 86:e00019. PubMed
5. Zhou T, *et al.* 2018. Mol Med Rep. 17:4883. PubMed
6. Coley WD *et al.* 2018. F1000Research. 2018 Mar 14 [revised 2018 Jan 1] 0.5125. PubMed
7. Xia Y, *et al.* 2018. Cell. 175:1059. PubMed
8. Habtetsion T *et al.* 2018. Cell metabolism. 28(2):228-242 . PubMed
9. Golan K *et al.* 2018. Cell stem cell. 23(4):572-585 . PubMed
10. Djokić V, *et al.* 2019. Front Immunol. 9:2891. PubMed
11. Alissafi T, *et al.* 2018. J Clin Invest. 128:3840. PubMed
12. Zhang W, *et al.* 2019. Oncol Lett. 17:815. PubMed
13. Tritama E, *et al.* 2018. Hum Vaccin Immunother. 14:1524. PubMed
14. Liang Z, *et al.* 2017. Autophagy. 14:505. PubMed
15. Bartley A, *et al.* 2018. Front Physiol. 9:1593. PubMed
16. Pedraza–Zamora CP, *et al.* 2017. Parasite Immunol. 39:4. PubMed
17. Niknam S, *et al.* 2018. Clin Cancer Res. 24:5735. PubMed
18. Katsumura KR, *et al.* 2018. Proc Natl Acad Sci U S A. 115:E10109. PubMed
19. Subramanian K, *et al.* 2019. Nat Microbiol. 4:62. PubMed
20. Choi H *et al.* 2019. Cell Rep. 27(3):806-819 . PubMed
21. Qi X, *et al.* 2019. Nat Commun. 10:2141. PubMed
22. Pobezinskaya EL *et al.* 2019. Front Immunol. 1.079861111 . PubMed
23. Kim JH, *et al.* 2019. Mol Med. 25:33. PubMed
24. Timilshina M, *et al.* 2020. Cell Reports. 27(10):2948-2961.e7.. PubMed
25. Zhou J, *et al.* 2019. Nat Commun. 10:2427. PubMed
26. Yang BH, *et al.* 2020. Cell Reports. 27(12):3629-3645.e6.. PubMed
27. Thomas S, *et al.* 2019. J Immunother Cancer. 7:214. PubMed
28. Blaszczak AM, *et al.* 2019. J Immunol. 202:2451. PubMed
29. Saleh MM, *et al.* 2019. Cell Host Microbe. 25:756. PubMed
30. Dmitrieva-Posocco O *et al.* 2019. Immunity. 50(1):166-180 . PubMed
31. Ji L *et al.* 2019. Immunity. 51(2):272-284 . PubMed
32. Periasamy S, *et al.* 2017. Nat Commun. 8:15564. PubMed
33. Valanparambil RM, *et al.* 2017. PLoS Pathog. 13:e1006647. PubMed
34. Zhang H, *et al.* 2019. Mol Cell. 76:110. PubMed
35. Wang X, *et al.* 2019. Cell Res. 29:787. PubMed
36. Sivapatham S, *et al.* 2019. Front Immunol. 1.865277778. PubMed
37. Hong Y, *et al.* 2019. J Extracell Vesicles. 8:1670893. PubMed
38. Hidalgo San Jose L, *et al.* 2020. Cell Rep. 30:69. PubMed
39. Zhao Y, *et al.* 2020. Front Immunol. 2.572222222. PubMed
40. Sarapulov AV, *et al.* 2020. Front Immunol. 11:599. PubMed
41. Wu N, *et al.* 2020. Cell Rep. 30:1129. PubMed
42. Tao Z, *et al.* 2019. Clin Cancer Res. 25:1113. PubMed
43. Kos S, *et al.* 2019. PLoS One. 14:e0217762. PubMed
44. Ouyang S, *et al.* 2019. J Immunol. 202:1441. PubMed
45. Liu YG, *et al.* 2019. FASEB J. 33:5018. PubMed
46. Yao C, *et al.* 2018. Autophagy. 1.854861111. PubMed
47. Khosravi N, *et al.* 2018. Cancer Immunol Res. 0.797222222. PubMed
48. Ramanan D, *et al.* 2020. Cell. 181(6):1276-1290. PubMed
49. Kamata T, *et al.* 2020. Cell Reports. 31(12):107802. PubMed
50. Cervera–Carrascon V, *et al.* 2020. Oncoimmunology. 9:1761229. PubMed
51. Wu J, *et al.* 2020. Sci Adv. 6:eaba3458. PubMed
52. Paul S, *et al.* 2018. Appl Microbiol Biotechnol. 102:8895. PubMed
53. Guan H, *et al.* 2012. PLoS One. 7:e35650. PubMed
54. Sega E, *et al.* 2008. Int J Radiat Oncol Biol Phys. 71:559. PubMed
55. Lua Y, *et al.* 2012. Mol Immunol. 52:229. PubMed
56. Chuang H, *et al.* 2014. Nat Commun. 5:4602. PubMed
57. Mencl S, *et al.* 2014. J Neuroimmunol. 274:125. PubMed
58. Zasłona Z, *et al.* 2014. J Immunol. 193:4245. PubMed
59. White C, *et al.* 2014. J Immunol. 193:5933. PubMed
60. Kmieciak M, *et al.* 2011. J Vis Exp. 47: 2381. PubMed
61. Hart B, *et al.* 2015. PLoS Negl Trop Dis. 9: 0004046. PubMed
62. Ito C, *et al.* 2015. PLoS One. 10: e0140808. PubMed
63. Uddback I, *et al.* 2016. Sci Rep. 6:20137. PubMed
64. Li Y, Kaneda T 2016. Sci Rep. 6: 25077. PubMed
65. Toomer K, *et al.* 2016. J Immunol. 196: 3665 - 3676. PubMed
66. Ahmari N, *et al.* 2016. Physiol Genomics. 48: 526 - 536. PubMed
67. Vigeland C, *et al.* 2016. PLoS One. 11: 0163288. PubMed
68. Lu X, *et al.* 2016. Nat Commun. 7: 12719. PubMed
69. Angela M, *et al.* 2016. Nat Commun. 7:13683. PubMed
70. D Kim, S Lee, H Jun 2016. Immunol Cell Biol. 10.1038/icb.2016.98. PubMed
71. Knocke S, *et al.* 2016. Cell Rep. 17:2234-2246. PubMed
72. Bryan Hart, Sunhee Lee 2016. PLoS Negl Trop Dis. 10:e0005229. PubMed
73. Aguilera T, *et al.* 2016. Nat Commun. 7:13898. PubMed
74. Timilshina M, *et al.* 2017. PLoS One. 10.1371/journal.pone.0168942. PubMed
75. Furuta Y, *et al.* 2017. PLoS One. 12(2):e0172509. PubMed

76. Zan H, *et al.* 2017. Nat Commun. 8:14244. PubMed
77. Gardenier J, *et al.* 2017. Nat Commun. 8:14345. PubMed
78. Tang Y, *et al.* 2020. Front Immunol. 11:585918. PubMed
79. Wang X, *et al.* 2020. Nat Commun. 4.505555556. PubMed
80. Martomo SA, *et al.* 2021. Mol Cancer Ther. 1.074305556. PubMed
81. Simoni L, *et al.* 2020. Cell Rep. 33:108330. PubMed
82. Jaeger N, *et al.* 2020. Cell Rep. 33:108331. PubMed
83. Faust HJ, *et al.* 2020. J Clin Invest. 130:5493. PubMed
84. Blaszczak AM, *et al.* 2020. Immunometabolism. 2:00. PubMed
85. Cortez MA, *et al.* 2020. Nat Commun. 3.819444444. PubMed
86. He C, *et al.* 2020. Clin Transl Med. 10:e39. PubMed
87. Ligon MM, *et al.* 2020. Mucosal Immunol. 1.172222222. PubMed
88. Wang Z, *et al.* 2020. J Clin Invest. 130:3717. PubMed
89. Aguilera TA, *et al.* 2020. Clin Cancer Res. 26:2972. PubMed
90. Wei Z, *et al.* 2021. Nat Commun. 0.805555556. PubMed
91. Chang YS, *et al.* 2020. Int J Mol Sci. 21:00. PubMed
92. Guo S, *et al.* 2021. Frontiers in Immunology. 11:614667. PubMed
93. Rosenkranz SC, *et al.* 2021. eLife. 10:00. PubMed
94. Da Mesquita S, *et al.* 2021. Science Advances. 7(21):. PubMed
95. Si J, *et al.* 2020. Cancer Cell. 38(4):551-566.e11. PubMed
96. Shannon JP, *et al.* 2021. Immunity. 54(2):276-290.e5. PubMed
97. Ma X, *et al.* 2021. Cell Metabolism. 33(5):1001-1012.e5. PubMed
98. Mitchell JE, *et al.* 2021. Cell Reports. 35(2):108966. PubMed
99. Altshuler A, *et al.* 2021. Cell Stem Cell. 28(7):1248-1261.e8. PubMed
100. Volberding PJ, *et al.* 2021. Cell Reports. 35(8):109160. PubMed

**RRID**	AB_312690 (BioLegend Cat. No. 100405)<br>AB_312691 (BioLegend Cat. No. 100406)

Antigen Details

**Structure**	Ig superfamily, 55 kD
**Distribution**	Majority of thymocytes, T cell subset
**Function**	TCR co-receptor, T cell activation
**Ligand/Receptor**	MHC class II molecule
**Cell Type**	Dendritic cells, T cells, Thymocytes, Tregs
**Biology Area**	Immunology
**Molecular Family**	CD Molecules
**Antigen References**	1. Barclay A, *et al.* 1997. The Leukocyte Antigen FactsBook Academic Press.<br>2. Bierer BE, *et al.* 1989. *Annu. Rev. Immunol.* 7:579.<br>3. Janeway CA. 1992. *Annu. Rev. Immunol.* 10:645.
**Gene ID**	12504

Related Protocols

Cell Surface Flow Cytometry Staining Protocol

Other Formats

APC anti-mouse CD4, Biotin anti-mouse CD4, FITC anti-mouse CD4, PE anti-mouse CD4, PE/Cyanine5 anti-mouse CD4, Purified anti-mouse CD4, PE/Cyanine7 anti-mouse CD4, APC/Cyanine7 anti-mouse CD4, Alexa Fluor® 647 anti-mouse CD4, Alexa Fluor® 488 anti-mouse CD4, Pacific Blue™ anti-mouse CD4, Alexa Fluor® 700 anti-mouse CD4, PerCP anti-mouse CD4, PerCP/Cyanine5.5 anti-mouse CD4, Brilliant Violet 421™ anti-mouse CD4, Ultra-LEAF™ Purified anti-mouse CD4, Alexa Fluor® 594 anti-mouse CD4, Brilliant Violet 711™ anti-mouse CD4, Brilliant Violet 510™ anti-mouse CD4, Brilliant Violet 605™ anti-mouse CD4, Brilliant Violet 785™ anti-mouse CD4, PE/Dazzle™ 594 anti-mouse CD4, APC/Fire™ 750 anti-mouse CD4, GoInVivo™ Purified anti-mouse CD4, Brilliant Violet 750™ anti-mouse CD4, Brilliant Violet 650™ anti-mouse CD4, Spark Blue™ 550 anti-mouse CD4, Spark NIR™ 685 anti-mouse CD4, KIRAVIA Blue 520™ anti-mouse CD4, PE/Fire™ 640 anti-mouse CD4, APC/Fire™ 810 anti-mouse CD4, PE/Fire™ 700 anti-mouse CD4, Spark Violet™ 538 anti-mouse CD4, Spark YG™ 593 anti-mouse CD4, Spark Blue™ 574 anti-mouse CD4 Antibody, Spark UV™ 387 anti-mouse CD4

Product Data

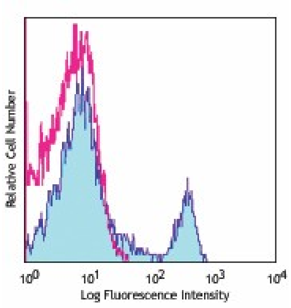

C57BL/6 mouse splenocytes were stained with CD4 (clone GK1.5) FITC (filled histogram) or rat IgG2b, κ FITC isotype control (open histogram).

For research use only. Not for diagnostic use. Not for resale. BioLegend will not be held responsible for patent infringement or other violations that may occur with the use of our products.

*These products may be covered by one or more Limited Use Label Licenses (see the BioLegend Catalog or our website, www.biolegend.com/ordering#license). BioLegend products may not be transferred to third parties, resold, modified for resale, or used to manufacture commercial products, reverse engineer functionally similar materials, or to provide a service to third parties without written approval of BioLegend. By use of these products you accept the terms and conditions of all applicable Limited Use Label Licenses. Unless otherwise indicated, these products are for research use only and are not intended for human or animal diagnostic, therapeutic or commercial use.

BioLegend Inc., 8999 BioLegend Way, San Diego, CA 92121 www.biolegend.com
Toll-Free Phone: 1-877-Bio-Legend (246-5343) Phone: (858) 768-5800 Fax: (877) 455-9587

BioLegend® Enabling Legendary Discovery™

Version: 2 Revision Date: 01/21/2014

APC anti-mouse CD8a Antibody

Catalog# / Size 100711 / 25 µg
100712 / 100 µg

Clone 53-6.7

Regulatory Status RUO

Other Names T8, Lyt2, Ly-2

Isotype Rat IgG2a, κ

Description CD8, also known as Lyt-2, Ly-2, or T8, consists of disulfide-linked α and β chains that form the α(CD8a)/β(CD8b) heterodimer and α/α homodimer. CD8a is a 34 kD protein that belongs to the immunoglobulin family. The CD8 α/β heterodimer is expressed on the surface of most thymocytes and a subset of mature TCR α/β T cells. CD8 expression on mature T cells is non-overlapping with CD4. The CD8 α/α homodimer is expressed on a subset of γ/δ TCR-bearing T cells, NK cells, intestinal intraepithelial lymphocytes, and lymphoid dendritic cells. CD8 is an antigen co-receptor on T cells that interacts with MHC class I on antigen-presenting cells or epithelial cells. CD8 promotes T cell activation through its association with the TCR complex and protein tyrosine kinase lck.

Product Details

Verified Reactivity Mouse

Antibody Type Monoclonal

Host Species Rat

Immunogen Mouse thymus or spleen

Formulation Phosphate-buffered solution, pH 7.2, containing 0.09% sodium azide.

Preparation The antibody was purified by affinity chromatography, and conjugated with APC under optimal conditions.

Concentration 0.2 mg/ml

Storage & Handling The CD8a antibody solution should be stored undiluted between 2°C and 8°C, and protected from prolonged exposure to light. **Do not freeze.**

Application FC - Quality tested

Recommended Usage Each lot of this antibody is quality control tested by immunofluorescent staining with flow cytometric analysis. For flow cytometric staining, the suggested use of this reagent is ≤ 0.25 µg per 10^6 cells in 100 µl volume. It is recommended that the reagent be titrated for optimal performance for each application.

Excitation Laser Red Laser (633 nm)

Application Notes Clone 53-6.7 antibody competes with clone 5H10-1 antibody for binding to thymocytes[3]. The 53-6.7 antibody has been reported to block antigen presentation via MHC class I and inhibit T cell responses to IL-2. This antibody has also been used for depletion of $CD8a^+$ cells. Additional reported applications (for the relevant formats) include: immunoprecipitation[1,3], *in vivo* and *in vitro* cell depletion[2,10,15], inhibition of CD8 T cell proliferation[3], blocking of cytotoxicity[3,4], immunohistochemical staining[5,6] of acetone-fixed frozen sections and zinc-fixed paraffin-embedded sections, and spatial biology (IBEX)[29,30]. Clone 53-6.7 is not recommended for immunohistochemistry of formalin-fixed paraffin sections. The Ultra-LEAF™ purified antibody (Endotoxin < 0.01 EU/µg, Azide-Free, 0.2 µm filtered) is recommended for functional assays or in vivo studies (Cat No. 100746).

Application References

1. Ledbetter JA, *et al.* 1979. *Immunol. Rev.* 47:63. (IHC, IP)
2. Hathcock KS. 1991. *Current Protocols in Immunology.* 3.4.1. (Deplete)
3. Takahashi K, *et al.* 1992. *P. Natl. Acad. Sci. USA* 89:5557. (Block, IP)
4. Ledbetter JA, *et al.* 1981. *J. Exp. Med.* 153:1503. (Block)

5. Hata H, *et al.* 2004. *J. Clin. Invest.* 114:582. (IHC)
6. Fan WY, *et al.* 2001. *Exp. Biol. Med.* 226:1045. (IHC)
7. Shih FF, *et al.* 2006. *J. Immunol.* 176:3438. (FC)
8. Kamimura D, *et al.* 2006. *J. Immunol.* 177:306.
9. Bouwer HGA, *et al.* 2006. *P. Natl. Acad. Sci. USA* 103:5102. (FC, Deplete)
10. Kao C, *et al.* 2005. *Int. Immunol.* 17:1607. PubMed
11. Ko SY, *et al.* 2005. *J. Immunol.* 175:3309. (FC) PubMed
12. Rasmussen JW, *et al.* 2006. *Infect. Immun.* 74:6590. PubMed
13. Lee CH, *et al.* 2009. *Clin. Cancer Res.* PubMed
14. Geiben-Lynn R, *et al.* 2008. *Blood* 112:4585. (Deplete) PubMed
15. Kingeter LM, *et al.* 2008. *J. Immunol.* 181:6244. PubMed
16. Guo Y, *et al.* 2008. *Blood* 112:480. PubMed
17. Andrews DM, *et al.* 2008. *J. Virol.* 82:4931. PubMed
18. Britschqui MR, *et al.* 2008. *J. Immunol.* 181:7681. PubMed
19. Kenna TJ, *et al.* 2008. *Blood* 111:2091. PubMed
20. Jordan JM, *et al.* 2008. *Infect. Immun.* 76:3717. PubMed
21. Todd DJ, *et al.* 2009. *J. Exp. Med.* 206:2151. PubMed
22. Bankoti J, *et al.* 2010. *Toxicol. Sci.* 115:422. (FC) PubMed
23. Medyouf H, *et al.* 2010. *Blood* 115:1175. PubMed
24. Riedl P, *et al.* 2009. *J. Immunol.* 183:370. PubMed
25. Apte SH, *et al.* 2010. *J. Immunol.* 185:998. PubMed
26. Bankoti J, *et al.* 2010. *Toxicol. Sci.* 115:422. (FC) PubMed
27. del Rio ML, *et al.* 2011. *Transpl. Int.* 24:501. (FC) PubMed
28. Cui L, *et al.* 2015. *J Control Release.* 206:220. PubMed
29. Radtke AJ, *et al.* 2020. *Proc Natl Acad Sci U S A.* 117:33455-65. (SB) PubMed
30. Radtke AJ, *et al.* 2022. *Nat Protoc.* 17:378-401. (SB) PubMed

Product Citations

1. Ishidome T *et al.* 2017. EBioMedicine. 22:89-99 . PubMed
2. Espinosa-Cueto P, *et al.* 2017. PLoS One.. 10.1371/journal.pone.0182126. PubMed
3. Glasner A,*et al.* 2017. Sci Rep.. 10.1038/s41598-017-12998-w. PubMed
4. Reismann D, *et al.* 2017. Nat Commun.. 10.1038/s41467-017-01538-9. PubMed
5. Chatterjee S *et al.* 2017. Cell metabolism. 27(1):85-100 . PubMed
6. Oyarce C, *et al.* 2018. Front Immunol. 8:1794. PubMed
7. Solanki A, *et al.* 2018. Development. 145. PubMed
8. Divan A, *et al.* 2018. PLoS One. 13:e0196893. PubMed
9. Espinosa JR, *et al.* 2018. Front Immunol. 9:1371. PubMed
10. Nam GH, *et al.* 2018. Nat Commun. 9:2165. PubMed
11. Tippimanchai DD, *et al.* 2018. Oncoimmunology. 7:e1438105. PubMed
12. Lee JS *et al.* 2018. Cell. 174(6):1559-1570 . PubMed
13. Yuzhu Hou *et al.* 2018. Immunity. 49(3):490-503 . PubMed
14. Gaya M *et al.* 2018. Cell. 172(3):517-533 . PubMed
15. Nakamura K, *et al.* 2018. Cancer Cell. 33:634. PubMed
16. Johnson JL *et al.* 2018. Immunity. 48(2):243-257 . PubMed
17. Logan K Smith *et al.* 2018. Immunity. 48(2):299-312 . PubMed
18. Cong J *et al.* 2018. Cell metabolism. 28(2):243-255 . PubMed
19. Chen S *et al.* 2018. Cell reports. 25(7):1729-1740 . PubMed
20. Wang D, *et al.* 2018. Immunity. 48:659. PubMed
21. Ren W, *et al.* 2018. Mucosal Immunol. 12:531. PubMed
22. Konishi Y, *et al.* 2018. iScience. 10:98. PubMed
23. Chen Z, *et al.* 2019. J Exp Med. 216:152. PubMed
24. Chakrabarti J, *et al.* 2018. Oncotarget. 9:37439. PubMed
25. Hayatsu N *et al.* 2017. Immunity. 47(2):268-283 . PubMed
26. Tran L, *et al.* 2017. Cancer Immunol Res. 5:1141. PubMed
27. Koliaraki V *et al.* 2019. Cell reports. 26(3):536-545 . PubMed
28. Funk KE, *et al.* 2019. J Neuroinflammation. 16:22. PubMed
29. Liu J, *et al.* 2019. J Exp Med. 216:656. PubMed
30. Li Y, *et al.* 2019. Oncol Rep. 41:2679. PubMed
31. Miska J *et al.* 2019. Cell reports. 27(1):226-237 . PubMed
32. Misumi I *et al.* 2019. Cell Rep. 27(2):514-524 . PubMed
33. Misumi I *et al.* 2019. Cell Rep. 27(5):1387-1396 . PubMed
34. Hu J, *et al.* 2019. Mol Ther Nucleic Acids. 16:650. PubMed
35. Luo Y *et al.* 2019. Cell reports. 26(7):1869-1879 . PubMed
36. Spangenberg E, *et al.* 2019. Nat Commun. 10:3758. PubMed
37. Schuster M, *et al.* 2017. J Immunol. 199:920. PubMed
38. Zhang F, *et al.* 2019. Nat Commun. 10:3974. PubMed
39. Budida R, *et al.* 2017. Eur J Immunol. 47:1819. PubMed
40. Lin YH, *et al.* 2018. Cancer Immunol Res. 0.25. PubMed
41. Schuster M, *et al.* 2019. Front Immunol. 10:1583. PubMed
42. Uchimura T *et al.* 2018. Immunity. 49(6):1049-1061 . PubMed
43. Paris J *et al.* 2019. Cell Stem Cell. 25(1):137-148 . PubMed
44. Song C, *et al.* 2019. Nat Commun. 10:3745. PubMed
45. Takeda Y, *et al.* 2018. Innate Immun. 24:323. PubMed
46. Li X, *et al.* 2017. Front Immunol. 8:1186. PubMed
47. Liu WL, *et al.* 2019. Nat Commun. 10:3199. PubMed
48. Li W, *et al.* 2019. Nat Commun. 10:3349. PubMed
49. Liu D *et al.* 2019. Immunity. 51(1):64-76 . PubMed
50. Zhang F, *et al.* 2019. Immunity. 50:738. PubMed
51. Li X *et al.* 2019. Immunity. 50(3):576-590 . PubMed

52. Riffelmacher T *et al.* 2017. Immunity. 47(3):466-480 . PubMed
53. Matsuo K, *et al.* 2018. J Immunol. 200:800. PubMed
54. Caronni N, *et al.* 2018. Cancer Res. 78:1685. PubMed
55. Lee JH, *et al.* 2018. Transgenic Res. 27:241. PubMed
56. Al–Zaher AA, *et al.* 2018. Mol Ther Oncolytics. 0.376388889. PubMed
57. Zhang J, *et al.* 2018. Oncoimmunology. 7:e1461301. PubMed
58. O'Malley G, *et al.* 2018. Cancer Immunol Res. 1.240277778. PubMed
59. Leal AS, *et al.* 2019. Sci Rep. 5.286111111. PubMed
60. Lin W, *et al.* 2019. EBioMedicine. 49:133. PubMed
61. Gupta SS, *et al.* 2019. Cell Rep. 29:1862. PubMed
62. Takaku S, *et al.* 2020. Integr Cancer Ther. 19:1534735419900798. PubMed
63. Morelli AE, *et al.* 2020. Cell Rep. 30:3448. PubMed
64. Ulaganathan VK, *et al.* 2020. Sci Rep. 10:8453. PubMed
65. Yoshida H, *et al.* 2019. Cell. 176:897. PubMed
66. Han P, *et al.* 2020. Sci Adv. 6:eaaz1580. PubMed
67. Soldevilla MM, *et al.* 2019. Mol Ther. 27:1878. PubMed
68. Chao Y, *et al.* 2020. Sci Adv. 6:eaaz4204. PubMed
69. Jiang W, *et al.* 2017. Sci Rep. 7:6501. PubMed
70. Kataru RP, *et al.* 2019. Cancer Immunol Res. 7:1345. PubMed
71. Xu X, *et al.* 2020. Chin Med. 15:33. PubMed
72. Riley JS, *et al.* 2020. Blood Adv. 4:1102. PubMed
73. Demircioglu F, *et al.* 2020. Nat Commun. 11:1290. PubMed
74. Maluski M, *et al.* 2019. J Clin Invest. 129:5108. PubMed
75. Schadt L, *et al.* 2020. Cell Reports. 29(5):1236-1248.e7.. PubMed
76. Bai C, *et al.* 2020. Mol Ther Oncolytics. 17:9. PubMed
77. Alikhanyan K, *et al.* 2020. Immun Inflamm Dis. 8:181. PubMed
78. Wang J, *et al.* 2020. J Hematol Oncol. 0.610416667. PubMed
79. Brummer G, *et al.* 2020. Oncogene. 39:2275. PubMed
80. Zheng Z, *et al.* 2020. Cell Death Dis. 0.684722222. PubMed
81. Horiguchi H, *et al.* 2019. Genes Dev. 33:1641. PubMed
82. Zhang NN, *et al.* 2020. Cell. 182(5):1271-1283. PubMed
83. Lu Y, *et al.* 2020. Cell. 180(6):1081-1097. PubMed
84. Blumenthal D, *et al.* 2020. eLife. 9:e55995.. PubMed
85. Chakraborty P, *et al.* 2020. Cell Reports. 28(7):1879-1893.e7.. PubMed
86. Zhang C, *et al.* 2020. Cell Metabolism. 31(1):148-161.e5.. PubMed
87. Mohamed E, *et al.* 2020. Immunity. 52(4):668-682.e7.. PubMed
88. Xu H, *et al.* 2020. Immunity. 51(4):696-708.e9.. PubMed
89. Mirando AC, *et al.* 2020. Oncoimmunology. 9:1760685. PubMed
90. Medyouf H, *et al.* 2010. Blood. 115:1175. PubMed
91. Sun S, *et al.* 2013. J Immunol. 190:6209. PubMed
92. Guo H, *et al.* 2014. J Leukoc Biol. 96:419. PubMed
93. Tassi I, *et al.* 2014. J Immunol. 193:4303. PubMed
94. Gibbins J, *et al.* 2014. Blood. 124:2953. PubMed
95. Shade K, *et al.* 2015. J Exp Med. 212:457. PubMed
96. Sasaki Y, *et al.* 2015. PLoS One. 10: 0131176. PubMed
97. Teo T, *et al.* 2015. J Virol. 89: 7955-7969. PubMed
98. Yin Q, *et al.* 2015. PLoS One. 10: 0137808. PubMed
99. Ku C, *et al.* 2015. Genes Dev. 29: 1930-1941. PubMed
100. Chen S, *et al.* 2015. Cancer Res . 7: 519-531. PubMed
101. Poczobutt J, *et al.* 2016. J Immunol. 196: 891 - 901. PubMed
102. Cook KD, *et al.* 2015. Immunity. 43:703-714. PubMed
103. Pelly V, *et al.* 2016. Mucosal Immunol. 10.1038/mi.2016.4. PubMed
104. Ma Z, *et al.* 2016. Clin Cancer Res. 22: 2969 - 2980. PubMed
105. Iwasaki Y, *et al.* 2016. PLoS One. 11: 0158282. PubMed
106. Vieyra-Garcia P, *et al.* 2016. Clin Cancer Res. 22: 3328 - 3339. PubMed
107. Latasa C, *et al.* 2016. PLoS One. 11: 0161216. PubMed
108. Matsumura K, *et al.* 2016. J Immunol. 197: 3233 - 3244. PubMed
109. Chen Q, *et al.* 2016. Nat Commun. 7:13193. PubMed
110. Vidy A, *et al.* 2016. PLoS One. 11:e0165361. PubMed
111. Felker A, Croy B 2016. J Leukoc Biol. 100: 645 - 655. PubMed
112. Wagner V, *et al.* 2016. Sci Transl Med. 8: 366ra164. PubMed
113. Eriksson E, *et al.* 2016. Gene Ther. 10.1038/gt.2016.80. PubMed
114. Swanson P, *et al.* 2016. PLoS Pathog. 12:e1006022. PubMed
115. Kramer K, *et al.* 2017. Mol Ther. 25(1):62-70. PubMed
116. Wu L, *et al.* 2020. Cell Rep. 33:108327. PubMed
117. Zheng DW, *et al.* 2020. Nat Commun. 3.865972222. PubMed
118. Holokai L, *et al.* 2020. Cancers (Basel). 12:00. PubMed
119. Choi JG, *et al.* 2020. Front Immunol. 11:598556. PubMed
120. Galeano Nio JL, *et al.* 2020. eLife. 9:00. PubMed
121. Gong N, *et al.* 2020. Nat Nanotechnol. 1.35625. PubMed
122. Leach SM, *et al.* 2020. Cell Rep. 33:108337. PubMed
123. Yin X, *et al.* 2020. Cell Rep. 33:108278. PubMed
124. Wanner-Seleznik GM, *et al.* 2020. Immunohorizons. 0.644444444. PubMed
125. Hoefflin R, *et al.* 2020. Nat Commun. 3.313194444. PubMed
126. Yi X, *et al.* 2020. Sci Adv. 6:eaba3546. PubMed
127. Ukidve A, *et al.* 2020. Proc Natl Acad Sci U S A. 117:17727. PubMed
128. Wang H, *et al.* 2020. Nat Mater. 1.655555556. PubMed
129. He C, *et al.* 2020. Clin Transl Med. 10:e39. PubMed
130. Bayik D, *et al.* 2020. Cancer Discov. 1.256944444. PubMed
131. Elahi S, *et al.* 2020. Stem Cell Res. 43:101710. PubMed

132. Aegerter H, *et al.* 2020. Nat Immunol. 0.975694444. PubMed
133. Chang YS, *et al.* 2020. Int J Mol Sci. 21:00. PubMed
134. Zheng H, *et al.* 2021. Frontiers in Cell and Developmental Biology. 9:641527. PubMed
135. Tartey S, *et al.* 2021. Cancer Research. 81(9):2358-2372. PubMed
136. Nahrendorf W, *et al.* 2021. eLife. 10:00. PubMed
137. Lebratti T, *et al.* 2021. eLife. 10:00. PubMed
138. Oguri Y, *et al.* 2020. Cell. 182(3):563-577.e20. PubMed
139. Alexandre YO, *et al.* 2020. Cell Reports. 33(13):108567. PubMed
140. Lu C, *et al.* 2020. Cancer Cell. 39(1):96-108.e6. PubMed
141. Shannon JP, *et al.* 2021. Immunity. 54(2):276-290.e5. PubMed
142. Han C, *et al.* 2021. Cell Reports. 34(6):108706. PubMed
143. Chen R, *et al.* 2021. Cell Reports. 34(7):108751. PubMed
144. Zeng W, *et al.* 2021. STAR Protocols. 2(1):100361. PubMed
145. Delacher M, *et al.* 2021. Immunity. 54(4):702-720.e17. PubMed
146. Dallari S, *et al.* 2021. Cell Host Microbe. 29(6):1014-1029.e8. PubMed
147. Liu Y, *et al.* 2021. Cell Metabolism. 33(6):1221-1233.e11. PubMed
148. Uzhachenko RV, *et al.* 2021. Cell Reports. 35(1):108944. PubMed
149. Mitchell JE, *et al.* 2021. Cell Reports. 35(2):108966. PubMed
150. Paiva RA, *et al.* 2021. Cell Reports. 35(2):108967. PubMed
151. Vaena S, *et al.* 2021. Cell Reports. 35(5):109076. PubMed
152. Christian LS, *et al.* 2021. Cell Reports. 35(6):109118. PubMed
153. Nanou A, *et al.* 2021. Cell Reports. 35(8):109168. PubMed
154. Dong L, *et al.* 2021. Cancer Cell. . PubMed

RRID AB_312750 (BioLegend Cat. No. 100711)
AB_312751 (BioLegend Cat. No. 100712)

Antigen Details

Structure Ig superfamily, CD8α chain, 34 kD

Distribution Most thymocytes, T cell subset, some NK cells, lymphoid dendritic cells

Function Co-receptor for TCR

Ligand/Receptor MHC class I molecule

Antigen References

1. Barclay A, *et al.* 1997. The Leukocyte Antigen FactsBook Academic Press.
2. Zamoyska R. 1994. *Immunity* 1:243.
3. Ellmeier W, *et al.* 1999. *Annu. Rev. Immunol.* 17:523.

Gene ID 12525

Related Protocols

Cell Surface Flow Cytometry Staining Protocol

Other Formats

APC anti-mouse CD8a, Biotin anti-mouse CD8a, FITC anti-mouse CD8a, PE anti-mouse CD8a, PE/Cyanine5 anti-mouse CD8a, Purified anti-mouse CD8a, PE/Cyanine7 anti-mouse CD8a, APC/Cyanine7 anti-mouse CD8a, Alexa Fluor® 488 anti-mouse CD8a, Alexa Fluor® 647 anti-mouse CD8a, Pacific Blue™ anti-mouse CD8a, Alexa Fluor® 700 anti-mouse CD8a, PerCP/Cyanine5.5 anti-mouse CD8a, PerCP anti-mouse CD8a, Brilliant Violet 421™ anti-mouse CD8a, Brilliant Violet 570™ anti-mouse CD8a, Brilliant Violet 650™ anti-mouse CD8a, Brilliant Violet 605™ anti-mouse CD8a, Ultra-LEAF™ Purified anti-mouse CD8a, Brilliant Violet 711™ anti-mouse CD8a, Brilliant Violet 785™ anti-mouse CD8a, Brilliant Violet 510™ anti-mouse CD8a, Purified anti-mouse CD8a (Maxpar® Ready), Alexa Fluor® 594 anti-mouse CD8a, PE/Dazzle™ 594 anti-mouse CD8a, APC/Fire™ 750 anti-mouse CD8a, GoInVivo™ Purified anti-mouse CD8a, TotalSeq™-A0002 anti-mouse CD8a, Spark Blue™ 550 anti-mouse CD8a, Spark NIR™ 685 anti-mouse CD8a, TotalSeq™-C0002 anti-mouse CD8a, TotalSeq™-B0002 anti-mouse CD8a, Spark YG™ 570 anti-mouse CD8a, PE/Fire™ 640 anti-mouse CD8a, PE/Fire™ 700 anti-mouse CD8a, Spark Blue™ 574 anti-mouse CD8a Antibody, Spark Violet™ 423 anti-mouse CD8a Antibody, Spark UV™ 387 anti-mouse CD8a

Product Data

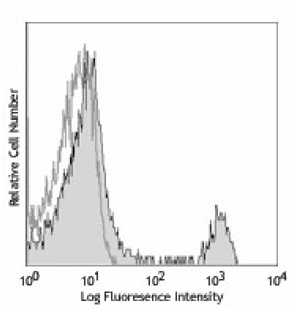

C57BL/6 mouse splenocytes were stained with CD8 (clone 53-6.7) APC (filled histogram) or rat IgG2a, κ APC isotype control (open histogram).

For research use only. Not for diagnostic use. Not for resale. BioLegend will not be held responsible for patent infringement or other violations that may occur with the use of our products.

*These products may be covered by one or more Limited Use Label Licenses (see the BioLegend Catalog or our website, www.biolegend.com/ordering#license). BioLegend products may not be transferred to third parties, resold, modified for resale, or used to manufacture commercial products, reverse engineer functionally similar materials, or to provide a service to third parties without written approval of BioLegend. By use of these products you accept the terms and conditions of all applicable Limited Use Label Licenses. Unless otherwise indicated, these products are for research use only and are not intended for human or animal diagnostic, therapeutic or commercial use.

BioLegend Inc., 8999 BioLegend Way, San Diego, CA 92121 www.biolegend.com
Toll-Free Phone: 1-877-Bio-Legend (246-5343) Phone: (858) 768-5800 Fax: (877) 455-9587

BioLegend® Enabling Legendary Discovery™

PE anti-mouse CD8a Antibody

**Catalog# / Size**	100707 / 50 µg<br>100708 / 200 µg
**Clone**	53-6.7
**Regulatory Status**	RUO
**Other Names**	T8, Lyt2, Ly-2
**Isotype**	Rat IgG2a, κ
**Description**	CD8, also known as Lyt-2, Ly-2, or T8, consists of disulfide-linked α and β chains that form the α(CD8a)/β(CD8b) heterodimer and α/α homodimer. CD8a is a 34 kD protein that belongs to the immunoglobulin family. The CD8 α/β heterodimer is expressed on the surface of most thymocytes and a subset of mature TCR α/β T cells. CD8 expression on mature T cells is non-overlapping with CD4. The CD8 α/α homodimer is expressed on a subset of γ/δ TCR-bearing T cells, NK cells, intestinal intraepithelial lymphocytes, and lymphoid dendritic cells. CD8 is an antigen co-receptor on T cells that interacts with MHC class I on antigen-presenting cells or epithelial cells. CD8 promotes T cell activation through its association with the TCR complex and protein tyrosine kinase lck.

Product Details

**Verified Reactivity**	Mouse
**Antibody Type**	Monoclonal
**Host Species**	Rat
**Immunogen**	Mouse thymus or spleen
**Formulation**	Phosphate-buffered solution, pH 7.2, containing 0.09% sodium azide.
**Preparation**	The antibody was purified by affinity chromatography, and conjugated with PE under optimal conditions.
**Concentration**	0.2 mg/ml
**Storage & Handling**	The CD8a antibody solution should be stored undiluted between 2°C and 8°C, and protected from prolonged exposure to light. **Do not freeze.**
**Application**	FC - Quality tested
**Recommended Usage**	Each lot of this antibody is quality control tested by immunofluorescent staining with flow cytometric analysis. For flow cytometric staining, the suggested use of this reagent is ≤ 0.25 µg per 10^6 cells in 100 µl volume. It is recommended that the reagent be titrated for optimal performance for each application.
**Excitation Laser**	Blue Laser (488 nm)<br>Green Laser (532 nm)/Yellow-Green Laser (561 nm)
**Application Notes**	Clone 53-6.7 antibody competes with clone 5H10-1 antibody for binding to thymocytes[3]. The 53-6.7 antibody has been reported to block antigen presentation via MHC class I and inhibit T cell responses to IL-2. This antibody has also been used for depletion of $CD8a^+$ cells. Additional reported applications (for the relevant formats) include: immunoprecipitation[1,3], *in vivo* and *in vitro* cell depletion[2,10,15], inhibition of CD8 T cell proliferation[3], blocking of cytotoxicity[3,4], immunohistochemical staining[5,6] of acetone-fixed frozen sections and zinc-fixed paraffin-embedded sections, and spatial biology (IBEX)[29,30]. Clone 53-6.7 is not recommended for immunohistochemistry of formalin-fixed paraffin sections. The Ultra-LEAF™ purified antibody (Endotoxin < 0.01 EU/µg, Azide-Free, 0.2 µm filtered) is recommended for functional assays or in vivo studies (Cat No. 100746).

Application References

1. Ledbetter JA, *et al.* 1979. *Immunol. Rev.* 47:63. (IHC, IP)
2. Hathcock KS. 1991. *Current Protocols in Immunology.* 3.4.1. (Deplete)
3. Takahashi K, *et al.* 1992. *P. Natl. Acad. Sci. USA* 89:5557. (Block, IP)

4. Ledbetter JA, *et al.* 1981. *J. Exp. Med.* 153:1503. (Block)
5. Hata H, *et al.* 2004. *J. Clin. Invest.* 114:582. (IHC)
6. Fan WY, *et al.* 2001. *Exp. Biol. Med.* 226:1045. (IHC)
7. Shih FF, *et al.* 2006. *J. Immunol.* 176:3438. (FC)
8. Kamimura D, *et al.* 2006. *J. Immunol.* 177:306.
9. Bouwer HGA, *et al.* 2006. *P. Natl. Acad. Sci. USA* 103:5102. (FC, Deplete)
10. Kao C, *et al.* 2005. *Int. Immunol.* 17:1607. PubMed
11. Ko SY, *et al.* 2005. *J. Immunol.* 175:3309. (FC) PubMed
12. Rasmussen JW, *et al.* 2006. *Infect. Immun.* 74:6590. PubMed
13. Lee CH, *et al.* 2009. *Clin. Cancer Res.* PubMed
14. Geiben-Lynn R, *et al.* 2008. *Blood* 112:4585. (Deplete) PubMed
15. Kingeter LM, *et al.* 2008. *J. Immunol.* 181:6244. PubMed
16. Guo Y, *et al.* 2008. *Blood* 112:480. PubMed
17. Andrews DM, *et al.* 2008. *J. Virol.* 82:4931. PubMed
18. Britschqui MR, *et al.* 2008. *J. Immunol.* 181:7681. PubMed
19. Kenna TJ, *et al.* 2008. *Blood* 111:2091. PubMed
20. Jordan JM, *et al.* 2008. *Infect. Immun.* 76:3717. PubMed
21. Todd DJ, *et al.* 2009. *J. Exp. Med.* 206:2151. PubMed
22. Bankoti J, *et al.* 2010. *Toxicol. Sci.* 115:422. (FC) PubMed
23. Medyouf H, *et al.* 2010. *Blood* 115:1175. PubMed
24. Riedl P, *et al.* 2009. *J. Immunol.* 183:370. PubMed
25. Apte SH, *et al.* 2010. *J. Immunol.* 185:998. PubMed
26. Bankoti J, *et al.* 2010. *Toxicol. Sci.* 115:422. (FC) PubMed
27. del Rio ML, *et al.* 2011. *Transpl. Int.* 24:501. (FC) PubMed
28. Cui L, *et al.* 2015. *J Control Release.* 206:220. PubMed
29. Radtke AJ, *et al.* 2020. *Proc Natl Acad Sci U S A.* 117:33455-65. (SB) PubMed
30. Radtke AJ, *et al.* 2022. *Nat Protoc.* 17:378-401. (SB) PubMed

Product Citations

1. Xu C, *et al.* 2017. Biochemical and Biophysical Research Communications. 10.1016/j.bbrc.2017.05.071. PubMed
2. Manrique-Rincón A, *et al.* 2017. Front Immunol. 10.3389/fimmu.2017.01150. PubMed
3. Lee YT, *et al.* 2018. PLoS One. 13:e0190868. PubMed
4. Zhang J, *et al.* 2018. Nature. 553:91. PubMed
5. Jia H, *et al.* 2018. Int J Oncol. 53:949. PubMed
6. Kagoya Y, *et al.* 2018. Nat Commun. 9:1915. PubMed
7. Echevarría-Vargas IM, *et al.* 2018. EMBO Mol Med. 10:e8446. PubMed
8. Habib S, *et al.* 2018. Infect Immun. 86:e00019. PubMed
9. Zhou T, *et al.* 2018. Mol Med Rep. 17:4883. PubMed
10. Yuzhu Hou *et al.* 2018. Immunity. 49(3):490-503 . PubMed
11. Liu Y, *et al.* 2018. Cancer Cell. 33:480. PubMed
12. Iwamoto H *et al.* 2018. Cell metabolism. 28(1):104-117 . PubMed
13. Xia Y, *et al.* 2018. Cell. 175:1059. PubMed
14. Lu Y, *et al.* 2018. Cancer Cell. 33:1048. PubMed
15. Dietmar Herndler-Brandstetter *et al.* 2018. Immunity. 48(4):716-729 . PubMed
16. Zhang W, *et al.* 2019. Oncol Lett. 17:815. PubMed
17. Perry JSA, *et al.* 2018. Immunity. 48:923. PubMed
18. Kong XF, *et al.* 2018. Nat Immunol. 19:973. PubMed
19. Subramanian K, *et al.* 2019. Nat Microbiol. 4:62. PubMed
20. Li J, *et al.* 2019. JCI Insight. 5. PubMed
21. Lee CG, *et al.* 2019. JCI Insight. 4:5. PubMed
22. Misumi I *et al.* 2019. Cell Rep. 27(2):514-524 . PubMed
23. Eisemann T, *et al.* 2019. Front Oncol. 9:187. PubMed
24. Misumi I *et al.* 2019. Cell Rep. 27(5):1387-1396 . PubMed
25. Cai Y, *et al.* 2019. Cell Rep. 27:3034. PubMed
26. Naing A *et al.* 2019. Cell reports. 26(5):1242-1257 . PubMed
27. Uchimura T *et al.* 2018. Immunity. 49(6):1049-1061 . PubMed
28. Paris J *et al.* 2019. Cell Stem Cell. 25(1):137-148 . PubMed
29. Niemann J, *et al.* 2019. Nat Commun. 10:3236. PubMed
30. Clemente–Casares X, *et al.* 2017. Immunity. 47:974. PubMed
31. Radulovic V, *et al.* 2020. Cell Reports. 27(10):2826-2836.e5.. PubMed
32. Du Q, *et al.* 2019. Front Microbiol. 10:2050. PubMed
33. Chen X, *et al.* 2019. Med Sci Monit. 25:5389. PubMed
34. van Montfoort N, *et al.* 2018. Cell. 175:1744. PubMed
35. Becher J, *et al.* 2018. Dev Cell. 47:592. PubMed
36. Jin X, *et al.* 2019. Mol Cell. 73:22:00. PubMed
37. Yuan X, *et al.* 2017. Elife. 6:e29540. PubMed
38. Oliveira AC *et al.* 2017. eLife. 6 pii: e30883. PubMed
39. Morabito KM, *et al.* 2017. Mucosal Immunol. 0.795138889. PubMed
40. Guo C, *et al.* 2017. Methods Mol Biol. 1709:345. PubMed
41. Frazier T, *et al.* 2018. Adipocyte. 0.41875. PubMed
42. van Erp EA, *et al.* 2019. Viruses. 0.811111111. PubMed
43. Yamashita M, *et al.* 2019. Cell Stem Cell. 25:357. PubMed
44. Wong YC, *et al.* 2019. J Virol. 93:e01154-19. PubMed
45. Huang X, *et al.* 2019. Cancer Immunol Res. 1.388888889. PubMed
46. Burr ML, *et al.* 2019. Cancer Cell. 36:385. PubMed
47. Meyer LK, *et al.* 2019. J Clin Invest. 130:863. PubMed
48. Yoshida H, *et al.* 2019. Cell. 176:897. PubMed
49. Davidson S, *et al.* 2020. Cell Reports. 31(7):107628. PubMed

50. Tsuchiya N, *et al.* 2020. Cell Reports. 29(1):162-175.e9.. PubMed
51. Demircioglu F, *et al.* 2020. Nat Commun. 11:1290. PubMed
52. Hsu SPC, *et al.* 2020. J Neurooncol. 146:417. PubMed
53. Zhao B, *et al.* 2020. Nat Commun. 11:908. PubMed
54. Chaurasiya S, *et al.* 2020. Oncoimmunology. 9:1729300. PubMed
55. Rana A, *et al.* 2020. Front Immunol. 11:536. PubMed
56. Frodermann V, *et al.* 2019. Nat Med. 25:1761. PubMed
57. Peng L, *et al.* 2020. Transl Lung Cancer Res. 0.892361111. PubMed
58. Parhi L, *et al.* 2020. Nat Commun. 2.721527778. PubMed
59. Thomas AM, *et al.* 2020. Journal of Cellular Physiology. 235(6):5120-5129.. PubMed
60. Kamata T, *et al.* 2020. Cell Reports. 31(12):107802. PubMed
61. Loh Z, *et al.* 2020. PLOS Pathogens. 16(7):e1008651. PubMed
62. Marchingo JM, *et al.* 2020. eLife. 9:e53725.. PubMed
63. Liang J, *et al.* 2020. Sci Adv. 6:eabc3646. PubMed
64. He Y, *et al.* 2020. Sci Adv. 6:eaba7589. PubMed
65. Paul S, *et al.* 2018. Appl Microbiol Biotechnol. 102:8895. PubMed
66. Hu Q, *et al.* 2018. Nat Biomed Eng. 0.660416667. PubMed
67. Rodriguez-Sosa M, *et al.* 2004. Infect Immun. 72:4552. PubMed
68. Lo W, Allen D 2012. Nat Immunol. 13:880. PubMed
69. Zhao X, *et al.* 2013. Int Immunopharmacol. 17:793. PubMed
70. Wong L, *et al.* 2013. J Biol Chem. 288:35170. PubMed
71. Chen X, *et al.* 2009. Hum Immunol. 70:79. PubMed
72. N'guessan P, *et al.* 2011. Antioxid Redox Signal. 15:1693. PubMed
73. LeBlanc P, *et al.* 2014. J Biol Chem. 289:7777. PubMed
74. Zhu E, *et al.* 2014. J Immunol. 192:5599. PubMed
75. Burrack K, *et al.* 2015. J Immunol. 194:678. PubMed
76. Lu X, *et al.* 2015. J Immunol. 194:2011. PubMed
77. Shade K, *et al.* 2015. J Exp Med. 212:457. PubMed
78. Sharma S, *et al.* 2015. J Immunol. 194:5529. PubMed
79. Ibrahim M, *et al.* 2016. J Evid Based Complementary Altern Med. 21: 171 - 176. PubMed
80. Yang E, *et al.* 2015. Acta Biochim Biophys Sin. 47: 588-596. PubMed
81. Kmieciak M, *et al.* 2011. J Vis Exp. 47: 2381. PubMed
82. Wong J, *et al.* 2015. Elife. 3: 07839. PubMed
83. Sido J, *et al.* 2015. J Leukoc Biol. 98: 435-447. PubMed
84. Flesch I, *et al.* 2015. J Immunol. 195: 2263-2272. PubMed
85. Wu Y, *et al.* 2015. J Immunol. 195: 2612-2623. PubMed
86. Burrack K, *et al.* 2015. PLoS Pathog. 11: e1005191. PubMed
87. Zhang Y, *et al.* 2015. PLoS Pathog. 11: e1005167. PubMed
88. Spinner C, *et al.* 2015. Sci Rep. 5:16269. PubMed
89. Schuldt N, *et al.* 2015. PLoS One. 10: 0145762. PubMed
90. Pai M, *et al.* 2016. JPEN J Parenter Enteral Nutr. 40: 383 - 391. PubMed
91. Chou T, *et al.* 2016. Nat Commun. 7:11904. PubMed
92. Wang Y, *et al.* 2016. Sci Rep. 6:31881. PubMed
93. Li B, Schmidt N 2016. PLoS One. 11: 0162427. PubMed
94. Felker A, Croy B 2016. J Leukoc Biol. 100: 645 - 655. PubMed
95. Angela M, *et al.* 2016. Nat Commun. 7:13683. PubMed
96. Aguilera T, *et al.* 2016. Nat Commun. 7:13898. PubMed
97. Hu HJ, *et al.* 2020. Cell Death Dis. 1.168055556. PubMed
98. Qi T, *et al.* 2020. Cell Rep. 33:108452. PubMed
99. Kang YA, *et al.* 2020. J Exp Med. 217:00:00. PubMed
100. Montel-Hagen A, *et al.* 2020. Cell Rep. 33:108320. PubMed
101. Cai Z, *et al.* 2020. Cell Rep. 31:107816. PubMed
102. Li J, *et al.* 2020. FASEB J. 34:3091. PubMed
103. Aguilera TA, *et al.* 2020. Clin Cancer Res. 26:2972. PubMed
104. Wei Z, *et al.* 2021. Nat Commun. 0.805555556. PubMed
105. Kovacs SB, *et al.* 2021. STAR Protoc. 2:100244. PubMed
106. Melo-Silva CR, *et al.* 2021. PLOS Pathogens. 17(5):e1009593. PubMed
107. Tran S, *et al.* 2020. Immunity. 53(3):627-640.e5. PubMed
108. Xu C, *et al.* 2020. Immunity. 53(2):417-428.e4. PubMed
109. Si J, *et al.* 2020. Cancer Cell. 38(4):551-566.e11. PubMed
110. Plumlee CR, *et al.* 2020. Cell Host Microbe. 29(1):68-82.e5. PubMed
111. Heyde A, *et al.* 2021. Cell. 184(5):1348-1361.e22. PubMed
112. Han C, *et al.* 2021. Cell Reports. 34(6):108706. PubMed
113. Lv K, *et al.* 2021. Cell Stem Cell. 28(7):1275-1290.e9. PubMed
114. Levine LS, *et al.* 2021. Immunity. 54(4):829-844.e5. PubMed
115. Mitchell JE, *et al.* 2021. Cell Reports. 35(2):108966. PubMed
116. Yu K, *et al.* 2021. Cell Reports. 35(12):109273. PubMed

RRID AB_312746 (BioLegend Cat. No. 100707)
AB_312747 (BioLegend Cat. No. 100708)

Antigen Details

Structure Ig superfamily, CD8α chain, 34 kD

Distribution Most thymocytes, T cell subset, some NK cells, lymphoid dendritic cells

Function Co-receptor for TCR

Ligand/Receptor MHC class I molecule

Antigen References
1. Barclay A, *et al.* 1997. The Leukocyte Antigen FactsBook Academic Press.
2. Zamoyska R. 1994. *Immunity* 1:243.
3. Ellmeier W, *et al.* 1999. *Annu. Rev. Immunol.* 17:523.

Gene ID 12525

Related Protocols

Cell Surface Flow Cytometry Staining Protocol

Other Formats

APC anti-mouse CD8a, Biotin anti-mouse CD8a, FITC anti-mouse CD8a, PE anti-mouse CD8a, PE/Cyanine5 anti-mouse CD8a, Purified anti-mouse CD8a, PE/Cyanine7 anti-mouse CD8a, APC/Cyanine7 anti-mouse CD8a, Alexa Fluor® 488 anti-mouse CD8a, Alexa Fluor® 647 anti-mouse CD8a, Pacific Blue™ anti-mouse CD8a, Alexa Fluor® 700 anti-mouse CD8a, PerCP/Cyanine5.5 anti-mouse CD8a, PerCP anti-mouse CD8a, Brilliant Violet 421™ anti-mouse CD8a, Brilliant Violet 570™ anti-mouse CD8a, Brilliant Violet 650™ anti-mouse CD8a, Brilliant Violet 605™ anti-mouse CD8a, Ultra-LEAF™ Purified anti-mouse CD8a, Brilliant Violet 711™ anti-mouse CD8a, Brilliant Violet 785™ anti-mouse CD8a, Brilliant Violet 510™ anti-mouse CD8a, Purified anti-mouse CD8a (Maxpar® Ready), Alexa Fluor® 594 anti-mouse CD8a, PE/Dazzle™ 594 anti-mouse CD8a, APC/Fire™ 750 anti-mouse CD8a, GoInVivo™ Purified anti-mouse CD8a, TotalSeq™-A0002 anti-mouse CD8a, Spark Blue™ 550 anti-mouse CD8a, Spark NIR™ 685 anti-mouse CD8a, TotalSeq™-C0002 anti-mouse CD8a, TotalSeq™-B0002 anti-mouse CD8a, Spark YG™ 570 anti-mouse CD8a, PE/Fire™ 640 anti-mouse CD8a, PE/Fire™ 700 anti-mouse CD8a, Spark Blue™ 574 anti-mouse CD8a Antibody, Spark Violet™ 423 anti-mouse CD8a Antibody, Spark UV™ 387 anti-mouse CD8a

Product Data

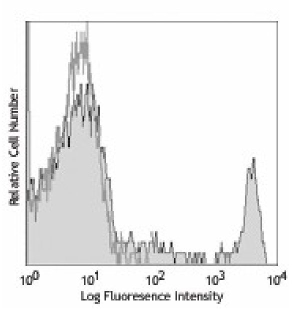

C57BL/6 mouse splenocytes were stained with CD8 (clone 53-6.7) PE (filled histogram) or rat IgG2a, κ PE isotype control (open histogram).

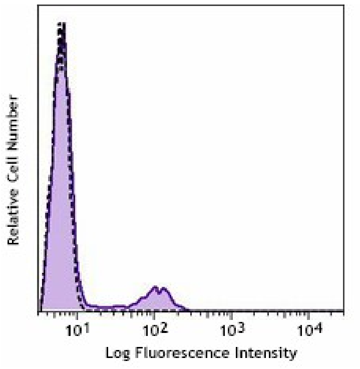

C57BL/6 mouse splenocytes were stained with CD8a (clone 53-6.7) PE (solid line) or rat IgG2a, κ PE isotype control (dashed line).

For research use only. Not for diagnostic use. Not for resale. BioLegend will not be held responsible for patent infringement or other violations that may occur with the use of our products.

*These products may be covered by one or more Limited Use Label Licenses (see the BioLegend Catalog or our website, www.biolegend.com/ordering#license). BioLegend products may not be transferred to third parties, resold, modified for resale, or used to manufacture commercial products, reverse engineer functionally similar materials, or to provide a service to third parties without written approval of BioLegend. By use of these products you accept the terms and conditions of all applicable Limited Use Label Licenses. Unless otherwise indicated, these products are for research use only and are not intended for human or animal diagnostic,

therapeutic or commercial use.

BioLegend Inc., 8999 BioLegend Way, San Diego, CA 92121 www.biolegend.com
Toll-Free Phone: 1-877-Bio-Legend (246-5343) Phone: (858) 768-5800 Fax: (877) 455-9587

Technical Data Sheet

PE Rat Anti-Mouse CD44

Product Information

**Material Number:**	553134
**Alternate Name:**	Pgp-1; Ly-24; H-CAM; HERMES; ECMR-III; Hyaluronate Receptor
**Size:**	0.1 mg
**Concentration:**	0.2 mg/ml
**Clone:**	IM7
**Immunogen:**	Dexamethasone-induced, SJL mouse spontaneous myeloid leukemia M1 cells myeloid leukemia M1
**Isotype:**	Rat IgG2b, κ
**Reactivity:**	QC Testing: Mouse
**RRID:**	AB_394649
**Storage Buffer:**	Aqueous buffered solution containing ≤0.09% sodium azide.

Description

The IM7 antibody specifically recognizes an epitope on both alloantigens and all isoforms of the CD44 glycoprotein (Pgp-1, Ly-24). The standard form of CD44, lacking variable exons and referred to as CD44H or CD44s, is widely expressed on hematopoietic and non-hematopoietic cells. CD44 isoforms encoded by variable exons are expressed on epithelial cells, but only at low levels on most leukocytes. Mice with the Ly-24.1 alloantigen (e.g., BALB/c, CBA/J, DBA/1, DBA/2) have relatively large subsets of CD44H+ T lymphocytes, while Ly-24.2 strains (e.g., A, AKR, CBA/N, C3H/He, C57BL, C57BR, C57L, C58, NZB, SJL, SWR, 129) have fewer CD44H+ T cells. CD44 is a cell adhesion receptor, and its principal ligand, hyaluronate, is a common component of extracellular matrices. Differential glycosylation of CD44 influences its binding to hyaluronate. Additional ligands include the cell surface form of CD74 and the cytokine osteopontin (Eta-1). Bone marrow- and thymus-derived progenitor cells capable of repopulating the thymus express CD44. In the periphery, the level of CD44 expression increases upon activation of B lymphocytes, CD4+ T cells, and CD8+ T cells; memory cells can be recognized by their CD44[hi] phenotype. The IM7 mAb inhibits established collagen-induced arthritis in DBA/1 mice. Moreover, it prevents CNS inflammation and clinical symptoms of experimental autoimmune encephalomyelitis. In contrast, the same antibody exacerbates experimental autoimmune thyroiditis in CBA/J mice. The IM7 mAb recognizes a different epitope from that recognized by mAb KM114, and the antibody pair can be used in ELISA to detect soluble CD44. It has been observed that IM7 antibody crossreacts with human, dog, cat, horse, cow, and pig leukocytes. Anti-human CD44, clone G44-26, and IM7 antibody compete for binding to human peripheral blood lymphocytes.

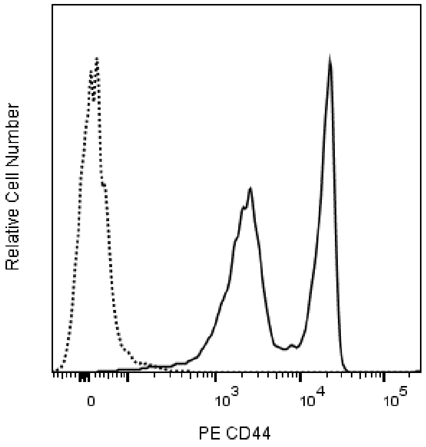

Flow cytometric analysis of CD44 expression on bone-marrow cells. *C57BL/6 mouse bone-marrow cells were stained with either PE Rat IgG2b, κ Isotype Control (Cat. No. 553989; dashed line histogram) or with the PE Rat Anti-Mouse CD44 antibody (Cat. No. 561860/553134; solid line histogram). The fluorescence histograms were derived from events with the forward and side light-scatter characteristics of viable bone marrow cells. Flow cytometry and data analysis were performed using a BD LSRFortessa™ X-20 Cell Analyzer System and FlowJo™ software. Data shown on this Technical Data Sheet are not lot specific.*

Preparation and Storage

Store undiluted at 4°C and protected from prolonged exposure to light. Do not freeze.

The monoclonal antibody was purified from tissue culture supernatant or ascites by affinity chromatography.

The antibody was conjugated to the dye under optimum conditions and unconjugated antibody and free dye were removed.

BD Biosciences

bdbiosciences.com

United States	Canada	Europe	Japan	Asia Pacific	Latin America/Caribbean
877.232.8995	866.979.9408	32.2.400.98.95	0120.8555.90	65.6861.0633	55.11.5185.9995

For country contact information, visit **bdbiosciences.com/contact**

Conditions: The information disclosed herein is not to be construed as a recommendation to use the above product in violation of any patents. BD Biosciences will not be held responsible for patent infringement or other violations that may occur with the use of our products. Purchase does not include or carry any right to resell or transfer this product either as a stand-alone product or as a component of another product. Any use of this product other than the permitted use without the express written authorization of Becton, Dickinson and Company is stictly prohibited.

For Research Use Only. Not for use in diagnostic or therapeutic procedures. Not for resale.

© 2017 BD. BD, the BD Logo and all other trademarks are property of Becton, Dickinson and Company.

Application Notes

Application

Flow cytometry	Routinely Tested

Recommended Assay Procedure:

BD® CompBeads can be used as surrogates to assess fluorescence spillover (compensation). When fluorochrome conjugated antibodies are bound to BD® CompBeads, they have spectral properties very similar to cells. However, for some fluorochromes there can be small differences in spectral emissions compared to cells, resulting in spillover values that differ when compared to biological controls. It is strongly recommended that when using a reagent for the first time, users compare the spillover on cell and BD® CompBeads to ensure that BD® CompBeads are appropriate for your specific cellular application.

Suggested Companion Products

Catalog Number	Name	Size	Clone
554656	Stain Buffer (FBS)	500 mL	(none)
554657	Stain Buffer (BSA)	500 mL	(none)
553989	PE Rat IgG2b, κ Isotype Control	0.1 mg	A95-1

Product Notices

1. Since applications vary, each investigator should titrate the reagent to obtain optimal results.
2. An isotype control should be used at the same concentration as the antibody of interest.
3. For fluorochrome spectra and suitable instrument settings, please refer to our Multicolor Flow Cytometry web page at www.bdbiosciences.com/colors.
4. Caution: Sodium azide yields highly toxic hydrazoic acid under acidic conditions. Dilute azide compounds in running water before discarding to avoid accumulation of potentially explosive deposits in plumbing.
5. Please refer to www.bdbiosciences.com/us/s/resources for technical protocols.
6. Please refer to http://regdocs.bd.com to access safety data sheets (SDS).

References

Bendelac A. Mouse NK1+ T cells. *Curr Opin Immunol.* 1995; 7(3):367-374. (Biology)

Brocke S, Piercy C, Steinman L, Weissman IL, Veromaa T. Antibodies to CD44 and integrin alpha4, but not L-selectin, prevent central nervous system inflammation and experimental encephalomyelitis by blocking secondary leukocyte recruitment. *Proc Natl Acad Sci U S A.* 1999; 96(12):6896-6901. (Clone-specific)

Budd RC, Cerottini JC, Horvath C, et al. Distinction of virgin and memory T lymphocytes. Stable acquisition of the Pgp-1 glycoprotein concomitant with antigenic stimulation. *J Immunol.* 1987; 138(10):3120-3129. (Biology)

Godfrey DI, Kennedy J, Suda T, Zlotnik A. A developmental pathway involving four phenotypically and functionally distinct subsets of CD 3-CD4-CD8- triple-negative adult mouse thymocytes defined by CD44 and CD25 expression. *J Immunol.* 1993; 150(10):4244-4252. (Biology)

Hathcock KS, Hirano H, Murakami S, Hodes RJ. CD44 expression on activated B cells. Differential capacity for CD44-dependent binding to hyaluronic acid. *J Immunol.* 1993; 151(12):6712-6722. (Biology)

Hyman R, Lesley J, Schulte R, Trotter J. Progenitor cells in the thymus: most thymus-homing progenitor cells in the adult mouse thymus bear Pgp-1 glycoprotein but not interleukin-2 receptor on their cell surface. *Cell Immunol.* 1986; 101(2):320-327. (Biology)

Ikawa T, Kawamoto H, Fujimoto S, Katsura Y. Commitment of common T/natural killer (NK) progenitors to unipotent T and NK progenitors in the murine fetal thymus revealed by a single progenitor assay. *J Exp Med.* 1999; 190(11):1617-1625. (Biology)

Katoh S, McCarthy JB, Kincade PW. Characterization of soluble CD44 in the circulation of mice. Levels are affected by immune activity and tumor growth. *J Immunol.* 1994; 153(8):3440-3449. (Clone-specific)

Katoh S, Zheng Z, Oritani K, Shimozato T, Kincade PW. Glycosylation of CD44 negatively regulates its recognition of hyaluronan. *J Exp Med.* 1995; 182(2):419-429. (Clone-specific)

Lesley J, Hyman R, Kincade PW. CD44 and its interaction with extracellular matrix. *Adv Immunol.* 1993; 54:271-335. (Biology)

Lesley J, Trowbridge IS. Genetic characterization of a polymorphic murine cell-surface glycoprotein. *Immunogenetics.* 1982; 15(3):313-320. (Immunogen)

Lynch F, Ceredig R. Mouse strain variation in Ly-24 (Pgp-1) expression by peripheral T cells and thymocytes: implications for T cell differentiation. *Eur J Immunol.* 1989; 19(2):223-229. (Biology)

MacDonald HR, Budd RC, Cerottini JC. Pgp-1 (Ly 24) as a marker of murine memory T lymphocytes. *Curr Top Microbiol Immunol.* 1990; 159:97-109. (Biology)

Matsumoto G, Nghiem MP, Nozaki N, Schmits R, Penninger JM. Cooperation between CD44 and LFA-1/CD11a adhesion receptors in lymphokine-activated killer cell cytotoxicity. *J Immunol.* 1998; 160(12):5781-5789. (Biology)

Naor D, Sionov RV, Ish-Shalom D. CD44: structure, function, and association with the malignant process. *Adv Cancer Res.* 1997; 71:241-319. (Biology)

Naujokas MF, Morin M, Anderson MS, Peterson M, Miller J. The chondroitin sulfate form of invariant chain can enhance stimulation of T cell responses through interaction with CD44. *Cell.* 1993; 74(2):257-268. (Biology)

Nedvetzki S, Walmsley M, Alpert E, Williams RO, Feldmann M, Naor D. CD44 involvement in experimental collagen-induced arthritis (CIA). *J Autoimmun.* 1999; 13(1):39-47. (Clone-specific)

Parish NM, Brennan FR, Cooke A. Anti-CD44 treatment does not prevent the extravasation of autopathogenic T cells to the thyroid in experimental autoimmune thyroiditis. *Immunology.* 1999; 97(3):533-539. (Clone-specific)

Spangrude GJ, Klein J, Heimfeld S, Aihara Y, Weissman IL. Two monoclonal antibodies identify thymic-repopulating cells in mouse bone marrow. *J Immunol.* 1989; 142(2):425-430. (Biology)

Trowbridge IS, Lesley J, Schulte R, Hyman R, Trotter J. Biochemical characterization and cellular distribution of a polymorphic, murine cell-surface glycoprotein expressed on lymphoid tissues. *Immunogenetics.* 1982; 15:299-312. (Immunogen)

Vremec D, Zorbas M, Scollay R, et al. The surface phenotype of dendritic cells purified from mouse thymus and spleen: investigation of the CD8 expression by a subpopulation of dendritic cells. *J Exp Med.* 1992; 176(1):47-58. (Clone-specific)

Weber GF, Ashkar S, Glimcher MJ, Cantor H. Receptor-ligand interaction between CD44 and osteopontin (Eta-1). *Science.* 1996; 271(5248):509-512. (Biology)

FITC anti-mouse CD62L Antibody

**Catalog# / Size**	104405 / 50 µg<br>104406 / 500 µg
**Clone**	MEL-14
**Regulatory Status**	RUO
**Other Names**	L-selectin, LECAM-1, Ly-22, LAM-1, MEL-14
**Isotype**	Rat IgG2a, κ
**Description**	CD62L is a 74-95 kD glycoprotein also known as L-selectin, LECAM-1, Ly-22, LAM-1, and MEL-14. It is a member of the selectin family and is expressed on the majority of B and naïve T cells, a subset of memory T cells, monocytes, granulocytes, most thymocytes, and a subset of NK cells. CD62L is important in lymphocyte homing to high endothelial venules (HEV) in peripheral lymph nodes and leukocyte "rolling" on activated endothelium. CD62L also contributes to neutrophil emigration at inflammatory sites. CD62L is rapidly shed from lymphocytes and neutrophils upon cellular activation and the expression levels of CD62L (in conjunction with other markers) have been used to distinguish naïve, effector, and memory T cells. CD62L has been reported to interact with CD34, GlyCAM-1, and MAdCAM-1.

Product Details

**Verified Reactivity**	Mouse
**Antibody Type**	Monoclonal
**Host Species**	Rat
**Immunogen**	C3H/eb mouse B lymphoma 38C-13
**Formulation**	Phosphate-buffered solution, pH 7.2, containing 0.09% sodium azide.
**Preparation**	The antibody was purified by affinity chromatography, and conjugated with FITC under optimal conditions.
**Concentration**	0.5 mg/ml
**Storage & Handling**	The antibody solution should be stored undiluted between 2°C and 8°C, and protected from prolonged exposure to light. **Do not freeze.**
**Application**	FC - Quality tested
**Recommended Usage**	Each lot of this antibody is quality control tested by immunofluorescent staining with flow cytometric analysis. For flow cytometric staining, the suggested use of this reagent is ≤ 0.25 µg per 10^6 cells in 100 µl volume. It is recommended that the reagent be titrated for optimal performance for each application.
**Excitation Laser**	Blue Laser (488 nm)
**Application Notes**	Additional reported applications (for the relevant formats) include: immunoprecipitation[1-3], complement-dependent cytotoxicity[4], *in vivo* and *in vitro* blocking of adhesion[1-3,5], and immunohistochemical staining of acetone-fixed frozen sections and zinc-fixed paraffin-embedded sections[6]. The Ultra-LEAF™ purified antibody (Endotoxin < 0.01 EU/µg, Azide-Free, 0.2 µm filtered) is recommended for functional assays (Cat. Nos. 104457-104462).

Application References

(PubMed link indicates BioLegend citation)

1. Gallatin WM, *et al.* 1983. *Nature* 304:30. (IP, Block)
2. Siegelman MH, *et al.* 1990. *Cell* 61:611. (IP, Block)
3. Lewinsohn DM, *et al.* 1987. *J. Immunol.* 138:4313. (IP, Block)
4. Iwabuchi K, *et al.* 1991. *Immunobiology* 182:161. (CMCD)
5. Pizcueta P, *et al.* 1994. *Am. J. Pathol.* 145:461.
6. Reichert RA, *et al.* 1986. *J. Immunol.* 136:3535. (IHC, FC)
7. Olver S, *et al.* 2006. *Cancer Res.* 66:571.
8. Fukushima A, *et al.* 2006. *Invest. Ophthalmol. Vis. Sci.* 47:657. PubMed
9. Benson MJ, *et al.* 2007. *J. Exp. Med.* doi:10.1084/jem.20070719. (FC) PubMed

10. Chappaz S, *et al.* 2007. *Blood* doi:10.1182/blood-2007-02-074245. (FC) PubMed
11. Lee JW, *et al.* 2006. *Nature Immunol.* 8:181.
12. Shigeta A, et al. 2008. *Blood* 112:4915 (FC) PubMed
13. de Vries VC, *et al.* 2009. *Am. J. Transplant.* 9:2270 PubMed

Product Citations

1. Espinosa JR, *et al.* 2018. Front Immunol. 9:1371. PubMed
2. Winter C, *et al.* 2018. Cell Metab. 28:175. PubMed
3. McCreedy DA *et al.* 2018. eNeuro. 5(4) pii: ENEURO. PubMed
4. Chen S, *et al.* 2018. Nat Commun. 9:5298. PubMed
5. Wu J *et al.* 2017. Immunity. 47(6):1114-1128 . PubMed
6. Kubo M, *et al.* 2018. Oncol Rep. 39:417. PubMed
7. Cuburu N, *et al.* 2019. J Immunol. 202:1250. PubMed
8. Nowill AE, *et al.* 2019. J Immunol. 203:1298. PubMed
9. Sema Kurtulus *et al.* 2018. Immunity. 50(1):181-194 . PubMed
10. Ying Zhang *et al.* 2017. Cancer cell. 32(3):377-391 . PubMed
11. Kishore M *et al.* 2017. Immunity. 47(5):875-889 . PubMed
12. Chen Z, *et al.* 2017. Mucosal Immunol. 10:58. PubMed
13. Wan X, *et al.* 2018. Nature. 560:107. PubMed
14. Mohammed RN, *et al.* 2019. Sci Rep. 4.185416667. PubMed
15. Cutler CE, *et al.* 2019. Glycobiology. 29:776. PubMed
16. Burrack AL, *et al.* 2019. Cell Rep. 28:2140. PubMed
17. Kashiwakura Y, *et al.* 2020. Clinical & Experimental Immunology. 202(1):119-135. PubMed
18. Hu Q, *et al.* 2018. Nat Biomed Eng. 0.660416667. PubMed
19. Prüfer S, *et al.* 2014. Immunobiology. 219:87. PubMed
20. Furuya Y, *et al.* 2014. J Virol. 88:9166. PubMed
21. Charlton J, *et al.* 2015. PLoS One. 10:119200. PubMed
22. Bonaccorsi-Riani E, *et al.* 2015. PLoS One. 10: 0136106. PubMed
23. Flaherty S, Reynolds J 2015. J Vis Exp. 98: 52739. PubMed
24. Kondo M, *et al.* 2016. J Immunol. 196: 563 - 572. PubMed
25. Cook KD, *et al.* 2015. Immunity. 43:703-714. PubMed
26. Wang Y, *et al.* 2020. Cell Death Dis. 1.169444444. PubMed
27. Zhuang Z, *et al.* 2021. J Exp Med. 218:00:00. PubMed
28. Chakraborty M, *et al.* 2021. Cell Reports. 34(2):108609. PubMed

RRID

AB_313092 (BioLegend Cat. No. 104405)
AB_313093 (BioLegend Cat. No. 104406)

Antigen Details

Structure Selectin, 95 kD (neutrophils) or 74 kD (lymphocytes)

Distribution Subsets of B and T cells, monocytes, granulocytes, subset of NK cells

Function Lymphocyte homing to HEV, rolling on activated endothelium

Ligand/Receptor CD34, GlyCAM-1, MAdCAM-1

Cell Type B cells, Granulocytes, Monocytes, Neutrophils, NK cells, T cells, Tregs

Biology Area Cell Adhesion, Cell Biology, Costimulatory Molecules, Immunology, Innate Immunity

Molecular Family Adhesion Molecules, CD Molecules

Antigen References

1. Barclay AN, *et al.* 1997. The Leukocyte Antigen FactsBook Academic Press.
2. Kishimoto TK, *et al.* 1990. *P. Natl. Acad. Sci. USA* 87:2244.
3. Tedder TF, *et al.* 1995. *J. Exp. Med.* 181:2259.

Gene ID 20343

Related Protocols

Cell Surface Flow Cytometry Staining Protocol

Other Formats

APC anti-mouse CD62L, Biotin anti-mouse CD62L, FITC anti-mouse CD62L, PE anti-mouse CD62L, PE/Cyanine5 anti-mouse CD62L, Purified anti-mouse CD62L, PE/Cyanine7 anti-mouse CD62L, Alexa Fluor® 488 anti-mouse CD62L, Alexa Fluor® 647 anti-

mouse CD62L, Pacific Blue™ anti-mouse CD62L, Alexa Fluor® 700 anti-mouse CD62L, APC/Cyanine7 anti-mouse CD62L, PerCP/Cyanine5.5 anti-mouse CD62L, PerCP anti-mouse CD62L, Brilliant Violet 421™ anti-mouse CD62L, Brilliant Violet 570™ anti-mouse CD62L, Brilliant Violet 605™ anti-mouse CD62L, Brilliant Violet 510™ anti-mouse CD62L, Purified anti-mouse CD62L (Maxpar® Ready), Brilliant Violet 711™ anti-mouse CD62L, Brilliant Violet 785™ anti-mouse CD62L, PE/Dazzle™ 594 anti-mouse CD62L, APC/Fire™ 750 anti-mouse CD62L, TotalSeq™-A0112 anti-mouse CD62L, Brilliant Violet 650™ anti-mouse CD62L, TotalSeq™-C0112 anti-mouse CD62L, Ultra-LEAF™ Purified anti-mouse CD62L, KIRAVIA Blue 520™ anti-mouse CD62L, TotalSeq™-B0112 anti-mouse CD62L

Product Data

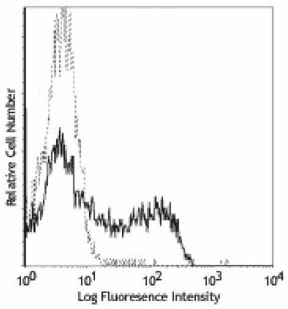

C57BL/6 bone marrow cells stained with MEL-14 FITC

For research use only. Not for diagnostic use. Not for resale. BioLegend will not be held responsible for patent infringement or other violations that may occur with the use of our products.

*These products may be covered by one or more Limited Use Label Licenses (see the BioLegend Catalog or our website, www.biolegend.com/ordering#license). BioLegend products may not be transferred to third parties, resold, modified for resale, or used to manufacture commercial products, reverse engineer functionally similar materials, or to provide a service to third parties without written approval of BioLegend. By use of these products you accept the terms and conditions of all applicable Limited Use Label Licenses. Unless otherwise indicated, these products are for research use only and are not intended for human or animal diagnostic, therapeutic or commercial use.

8999 BioLegend Way, San Diego, CA 92121 www.biolegend.com
Toll-Free Phone: 1-877-Bio-Legend (246-5343) Phone: (858) 768-5800 Fax: (877) 455-9587

Goat anti-Mouse IgG (H+L) Cross-Adsorbed Secondary Antibody, Alexa Fluor™ 647

Product Details	
Size	1 mg
Species Reactivity	Mouse
Host/Isotype	Goat / IgG
Class	Polyclonal
Type	Secondary Antibody
Conjugate	Alexa Fluor™ 647
Immunogen	Gamma Immunoglobins Heavy and Light chains
Form	Liquid
Concentration	2 mg/mL
Purification	purified
Storage buffer	PBS, pH 7.5
Contains	5mM sodium azide
Storage conditions	4° C, store in dark
RRID	AB_2535804

Applications	Tested Dilution	Publications
Immunohistochemistry (IHC)	Assay-dependent	-
Immunocytochemistry (ICC/IF)	2 µg/mL	-
Flow Cytometry (Flow)	1-10 µg/mL	-

Product Specific Information

To minimize cross-reactivity, these goat anti-mouse IgG (H+L) whole secondary antibodies have been affinity purified and cross-adsorbed against human IgG and human serum prior to conjugation. Cross-adsorption or pre-adsorption is a purification step to increase specificity of the antibody resulting in higher sensitivity and less background staining. The secondary antibody solution is passed through a column matrix containing immobilized serum proteins from potentially cross-reactive species. Only the nonspecific-binding secondary antibodies are captured in the column, and the highly specific secondaries flow through. The benefits of this extra step are apparent in multiplexing/multicolor-staining experiments (e.g., flow cytometry) where there is potential cross-reactivity with other primary antibodies or in tissue/cell fluorescent staining experiments where there are may be the presence of endogenous immunoglobulins.

Alexa Fluor dyes are among the most trusted fluorescent dyes available today. Invitrogen™ Alexa Fluor 647 dye is a near-infrared-fluorescent dye with excitation ideally suited to the 647 nm laser line. For stable signal generation in imaging and flow cytometry, Alexa Fluor 647 dye is pH-insensitive over a wide molar range. Probes with high fluorescence quantum yield and high photostability allow detection of low-abundance biological structures with great sensitivity. Alexa Fluor 647 dye molecules can be attached to proteins at high molar ratios without significant self-quenching, enabling brighter conjugates and more sensitive detection. The degree of labeling for each conjugate is typically 2-8 fluorophore molecules per IgG molecule; the exact degree of labeling is indicated on the certificate of analysis for each product lot.

Using conjugate solutions: Centrifuge the protein conjugate solution briefly in a microcentrifuge before use; add only the

supernatant to the experiment. This step will help eliminate any protein aggregates that may have formed during storage, thereby reducing nonspecific background staining. Because staining protocols vary with application, the appropriate dilution of antibody should be determined empirically. For the fluorophore-labeled antibodies a final concentration of 1-10 µg/mL should be satisfactory for most immunohistochemistry and flow cytometry applications.

Product will be shipped at Room Temperature.

Product Images For Goat anti-Mouse IgG (H+L) Cross-Adsorbed Secondary Antibody, Alexa Fluor™ 647

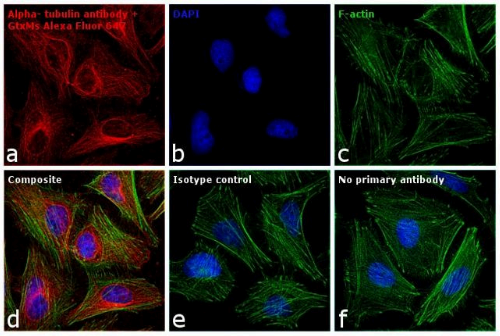

Mouse IgG (H+L) Cross-Adsorbed Secondary Antibody (A-21235) in ICC/IF
Immunofluorescence analysis of Goat anti-Mouse IgG (H+L) Cross-Adsorbed Secondary Antibody, Alexa Fluor® 647 conjugate was performed using HeLa cells stained with alpha Tubulin (236-10501) Mouse Monoclonal Antibody (Product # A11126). The cells were fixed with 4% paraformaldehyde for 10 minutes, permeabilized with 0.1% Triton™ X-100 for 10 minutes, blocked with 1% BSA for 1 hour and labeled with 2 µg/mL primary antibody for 3 hours at room temperature. Goat anti-Mouse IgG (H+L) Cross-Adsorbed Secondary Antibody, Alexa Fluor® 647 (Product # A-21235) was used at a concentration of 2 µg/mL in phosphate buffered saline containing 0.2% BSA for 45 minutes at room temperature, for detection of alpha Tubulin in the cytoplasm (Panel a: red). Nuclei (Panel b: blue) were stained with DAPI in SlowFade® Gold Antifade Mountant (Product # S36938). F-actin was stained with Alexa Fluor® 488 Phalloidin (Product # A12379), 1:300) (Panel c: green). Panel d represents the composite image. No nonspecific staining was observed with the secondary antibody alone (panel f), or with an isotype control (panel e). The images were captured at 60X magnification.

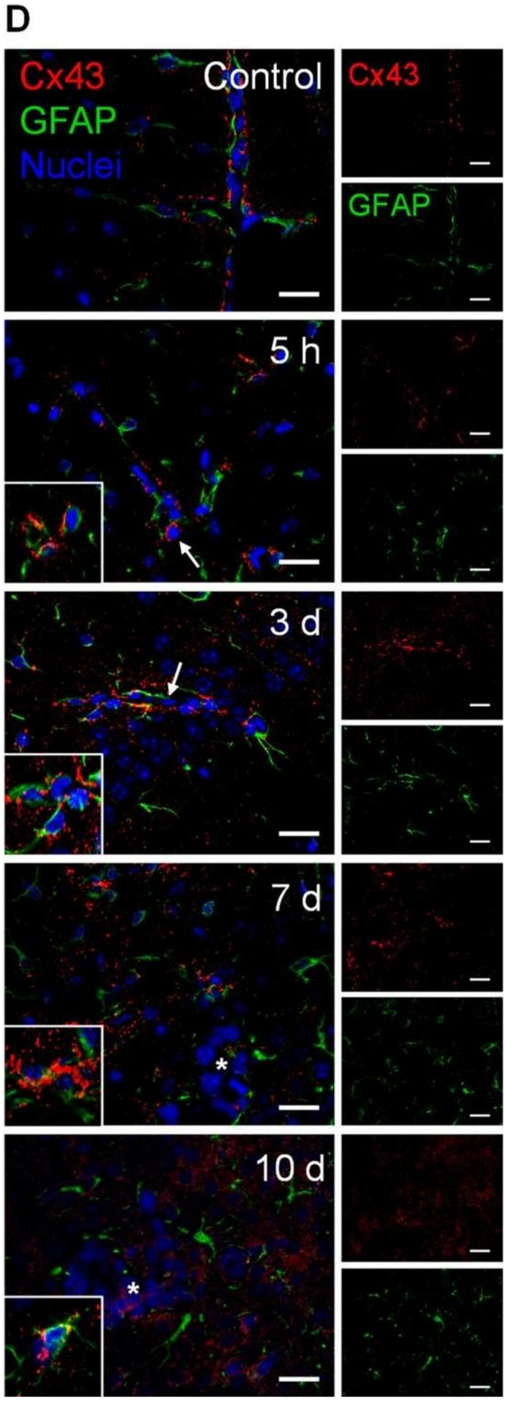

Mouse IgG (H+L) Cross-Adsorbed Secondary Antibody (A-21235) in ICC/IF

Cell crosstalk in breast cancer (BC) brain metastases occurs via gap junctions. The 4T1 cells or vehicle (controls) were inoculated in the carotid arteries of female Balb/c mice and hippocampal sections were analyzed after 5 h (h), 3, 7 and 10 days (d). (A) Double labelling of connexin 43 (Cx43, red) and the epithelial marker tomato lectin (green) showed that Cx43 is expressed in contact areas between endothelial cells and BC cells (arrows), in the vicinity of vimentin (yellow)- and cytokeratin (purple)-positive BC cells, as well as between adjacent BC cells (asterisks and magnified squares). Nuclei (blue) were counterstained with Hoechst 33342. Scale bar: 20 µm. Semi-quantitative analysis of Cx43 immunoreactivity per vessel (B) and per metastasis (C) revealed a peak at 7 d. (D) Double labelling of Cx43 (red) and the astrocyte marker glial fibrillary acidic protein (GFAP, green) showed the expression of this gap junction protein in contact areas among astrocytes (magnified squares) and between astrocytes and BC cells (arrows) but more distant from metastatic lesions (asterisk). Nuclei (blue) were counterstained with Hoechst 33342. Scale bar: 20 µm. (E) Semi-quantitative analysis of Cx43–GFAP colocalization revealed a peak at 7 d. Statistical differences are denoted as * $p < 0.05$ vs. control and # $p < 0.05$, ## $p < 0.01$ between indicated groups. Data are mean ± SEM, n = 3. Image collected and cropped by CiteAb from the following publication (), licensed under a CC BY license.

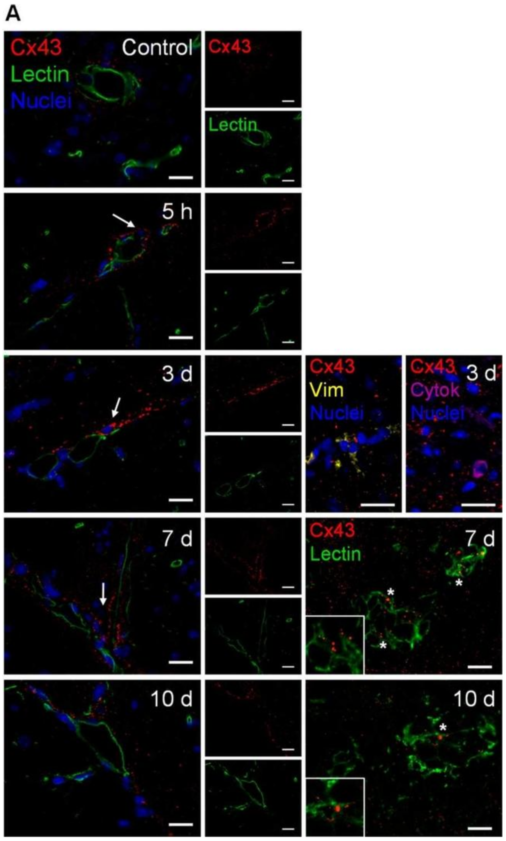

Mouse IgG (H+L) Cross-Adsorbed Secondary Antibody (A-21235) in ICC/IF
Cell crosstalk in breast cancer (BC) brain metastases occurs via gap junctions. The 4T1 cells or vehicle (controls) were inoculated in the carotid arteries of female Balb/c mice and hippocampal sections were analyzed after 5 h (h), 3, 7 and 10 days (d). (A) Double labelling of connexin 43 (Cx43, red) and the epithelial marker tomato lectin (green) showed that Cx43 is expressed in contact areas between endothelial cells and BC cells (arrows), in the vicinity of vimentin (yellow)- and cytokeratin (purple)-positive BC cells, as well as between adjacent BC cells (asterisks and magnified squares). Nuclei (blue) were counterstained with Hoechst 33342. Scale bar: 20 µm. Semi-quantitative analysis of Cx43 immunoreactivity per vessel (B) and per metastasis (C) revealed a peak at 7 d. (D) Double labelling of Cx43 (red) and the astrocyte marker glial fibrillary acidic protein (GFAP, green) showed the expression of this gap junction protein in contact areas among astrocytes (magnified squares) and between astrocytes and BC cells (arrows) but more distant from metastatic lesions (asterisk). Nuclei (blue) were counterstained with Hoechst 33342. Scale bar: 20 µm. (E) Semi-quantitative analysis of Cx43–GFAP colocalization revealed a peak at 7 d. Statistical differences are denoted as * $p < 0.05$ vs. control and # $p < 0.05$, ## $p < 0.01$ between indicated groups. Data are mean ± SEM, n = 3. Image collected and cropped by CiteAb from the following publication (), licensed under a CC BY license.

View more figures on thermofisher.com

1032 References

Assessing the response of human primary macrophages to defined fibrous architectures fabricated by melt electrowriting. Bioact Mater (2023)

Human immunomodulatory ligand B7-1 mediates synaptic remodeling via the p75 neurotrophin receptor. J Clin Invest (2022)

Presence of chondroitin sulphate and requirement for heparan sulphate biosynthesis in the developing zebrafish inner ear. Front Cell Dev Biol (2022)

devCellPy is a machine learning-enabled pipeline for automated annotation of complex multilayered single-cell transcriptomic data. Nat Commun (2022)

LINE-1 activation in the cerebellum drives ataxia. Neuron (2022)

For Research Use Only. Not for use in diagnostic procedures. Not for resale without express authorization. Products are warranted to operate or perform substantially in conformance with published Product specifications in effect at the time of sale, as set forth in the Production documentation, specifications and/or accompanying package inserts ("Documentation"). No claim of suitability for use in applications regulated by FDA is made. The warranty provided herein is valid only when used by properly trained individuals. Unless otherwise stated in the Documentation, this warranty is limited to one year from date of shipment when the Product is subjected to normal, proper and intended usage. This warranty does not extend to anyone other than the Buyer. Any model or sample furnished to Buyer is merely illustrative of the general type and quality of goods and does not represent that any Product will conform to such model or sample. NO OTHER WARRANTIES, EXPRESS OR IMPLED, ARE GRANTED INCLUDING WITHOUT LIMITATION, IMPLIED WARRANTIES OF MERCHANTABILITY, FITNESS FOR ANY PARTICULAR PURPOSE, OR NON INFRINGEMENT. BUYER'S EXCLUSIVE REMEDY FOR NON-CONFORMING PRODUCTS DURING THE WARRANTY PERIOD IS LIMITED TO REPAIR, REPLACEMENT OF OR REFUND FOR THE NON-CONFORMING PRODUCT(S) AT SELLER'S SOLE OPTION. THERE IS NO OBLIGATION TO REPAIR, REPLACE OR REFUND FOR PRODUCTS AS THE RESULT OF (I) ACCIDENT, DISASTER OR EVENT OF FORCE MAJEURE, (II) MISUSE, FAULT OR NEGLIGENCE OF OR BY BUYER, (III) USE OF THE PRODUCTS IN A MANNER FOR WHICH THEY WERE NOT DESIGNED, OR (IV) IMPROPER STORAGE AND HANDLING OF THE PRODUCTS. Unless otherwise expressly stated on the Product or in the documentation accompanying the Product, the Product is intended for research only and is not to be used for any other purpose, including without limitation, unauthorized commercial uses, in vitro diagnostic uses, ex vivo or in vivo therapeutic uses, or any type of consumption by or application to human or animals.

SANTA CRUZ BIOTECHNOLOGY, INC.

SATB1 (C-6): sc-376096

BACKGROUND

The homeoproteins CCAAT displacement protein (CDP) and special AT-rich sequence binding protein 1 (SATB1) are transcriptional repressors of many cellular genes, and they participate in cell development and cell type differentiation. SATB1 is expressed primarily in thymocytes, and, like CDP, it also contains a distinct homeobox DNA-binding domain that is essential for DNA binding. SATB1 and CDP interact through these homeodomains and synergistically function as mediators of gene expression. SATB1 contains an additional domain that has a higher affinity for DNA and specifically facilitates the direct association between SATB1 and the nuclear matrix attachment regions (MARs) of DNA. MARs are specific DNA sequences that bind to the nuclear matrix and form the base of chromosomal loops that organize the chromosomes and regulate DNA transcription and replication within the nucleus. The association of SATB1 with the core unwinding element within the base-unpairing region of MARs requires both the MAR and homeobox binding domains of SATB1.

REFERENCES

1. Dickinson, L.A., et al. 1997. An atypical homeodomain in SATB1 promotes specific recognition of the key structural element in a matrix attachment region. J. Biol. Chem. 272: 11463-11470.
2. Banan, M., et al. 1997. Interaction of the nuclear matrix-associated region (MAR)-binding proteins, SATB1 and CDP/Cux, with a MAR element (L2a) in an upstream regulatory region of the mouse CD8a gene. J. Biol. Chem. 272: 18440-18452.

CHROMOSOMAL LOCATION

Genetic locus: SATB1 (human) mapping to 3p24.3; Satb1 (mouse) mapping to 17 C.

SOURCE

SATB1 (C-6) is a mouse monoclonal antibody raised against amino acids 241-310 mapping within an internal region of SATB1 of human origin.

PRODUCT

Each vial contains 200 µg IgG_1 kappa light chain in 1.0 ml of PBS with < 0.1% sodium azide and 0.1% gelatin. Also available as TransCruz reagent for Gel Supershift and ChIP applications, sc-376096 X, 200 µg/0.1 ml.

SATB1 (C-6) is available conjugated to agarose (sc-376096 AC), 500 µg/0.25 ml agarose in 1 ml, for IP; to HRP (sc-376096 HRP), 200 µg/ml, for WB, IHC(P) and ELISA; to either phycoerythrin (sc-376096 PE), fluorescein (sc-376096 FITC), Alexa Fluor® 488 (sc-376096 AF488), Alexa Fluor® 546 (sc-376096 AF546), Alexa Fluor® 594 (sc-376096 AF594) or Alexa Fluor® 647 (sc-376096 AF647), 200 µg/ml, for WB (RGB), IF, IHC(P) and FCM; and to either Alexa Fluor® 680 (sc-376096 AF680) or Alexa Fluor® 790 (sc-376096 AF790), 200 µg/ml, for Near-Infrared (NIR) WB, IF and FCM.

Alexa Fluor® is a trademark of Molecular Probes, Inc., Oregon, USA

STORAGE

Store at 4° C, **DO NOT FREEZE**. Stable for one year from the date of shipment. Non-hazardous. No MSDS required.

APPLICATIONS

SATB1 (C-6) is recommended for detection of SATB1 of mouse, rat and human origin by Western Blotting (starting dilution 1:100, dilution range 1:100-1:1000), immunoprecipitation [1-2 µg per 100-500 µg of total protein (1 ml of cell lysate)], immunofluorescence (starting dilution 1:50, dilution range 1:50-1:500), immunohistochemistry (including paraffin-embedded sections) (starting dilution 1:50, dilution range 1:50-1:500) and solid phase ELISA (starting dilution 1:30, dilution range 1:30-1:3000).

Suitable for use as control antibody for SATB1 siRNA (h): sc-36460, SATB1 siRNA (m): sc-36461, SATB1 shRNA Plasmid (h): sc-36460-SH, SATB1 shRNA Plasmid (m): sc-36461-SH, SATB1 shRNA (h) Lentiviral Particles: sc-36460-V and SATB1 shRNA (m) Lentiviral Particles: sc-36461-V.

SATB1 (C-6) X TransCruz antibody is recommended for Gel Supershift and ChIP applications.

Molecular Weight (predicted) of SATB1 isoforms: 86/89 kDa.

Molecular Weight (observed) of SATB1: 115 kDa.

Positive Controls: CTLL-2 cell lysate: sc-2242, MOLT-4 cell lysate: sc-2233 or Jurkat whole cell lysate: sc-2204.

DATA

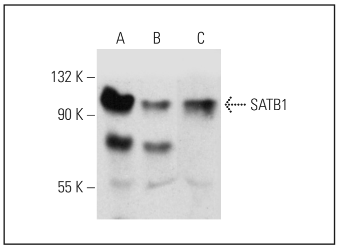

SATB1 (C-6): sc-376096. Western blot analysis of SATB1 expression in Jurkat (**A**), MOLT-4 (**B**) and CTLL-2 (**C**) whole cell lysates.

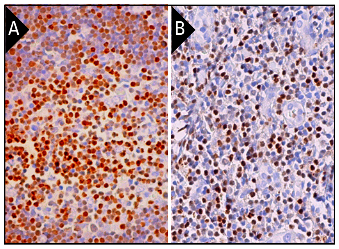

SATB1 (C-6): sc-376096. Immunoperoxidase staining of formalin fixed, paraffin-embedded human fetal thymus tissue showing nuclear staining of cortical cells and medullary cells (**A**). Immunoperoxidase staining of formalin fixed, paraffin-embedded human lymph node tissue showing nuclear staining of subset of cells in non-germinal center (**B**).

SELECT PRODUCT CITATIONS

1. Kumamaru, H., et al. 2019. Regenerating corticospinal axons innervate phenotypically appropriate neurons within neural stem cell grafts. Cell Rep. 26: 2329-2339.e4.
2. Verma, D.K., et al. 2021. α-synuclein preformed fibrils induce cellular senescence in Parkinson's disease models. Cells 10: 1694.
3. Hernández-Vivanco, A., et al. 2022. Sex-specific regulation of inhibition and network activity by local aromatase in the mouse hippocampus. Nat. Commun. 13: 3913.

RESEARCH USE

For research use only, not for use in diagnostic procedures.

abcam

Product datasheet

Anti-CTCF antibody ab70303

★★★★☆ 12 Abreviews 63 References 4 Images

Overview

**Product name**	Anti-CTCF antibody
**Description**	Rabbit polyclonal to CTCF
**Host species**	Rabbit
**Tested applications**	**Suitable for:** IHC-Fr, WB, IP, IHC-P
**Species reactivity**	**Reacts with:** Mouse, Human<br>**Predicted to work with:** Rat, Cow, Pig, Chimpanzee, Rhesus monkey, Gorilla, Orangutan, Elephant
**Immunogen**	Synthetic peptide corresponding to Human CTCF aa 650-750.<br>Database link: P49711
**Positive control**	IP: Jurkat whole cell lysate. IHC-P: Human lung cancer tissue, mouse renal cancer tissue. WB: Jurkat, HEK293T and HeLa whole cell lysate.
**General notes**	The Life Science industry has been in the grips of a reproducibility crisis for a number of years. Abcam is leading the way in addressing this with our range of recombinant monoclonal antibodies and knockout edited cell lines for gold-standard validation. Please check that this product meets your needs before purchasing.<br>If you have any questions, special requirements or concerns, please send us an inquiry and/or contact our Support team ahead of purchase. Recommended alternatives for this product can be found below, along with publications, customer reviews and Q&As

Properties

**Form**	Liquid
**Storage instructions**	Shipped at 4°C. Store at +4°C short term (1-2 weeks). Upon delivery aliquot. Store at -20°C. Avoid freeze / thaw cycle.
**Storage buffer**	pH: 6.8<br>Preservative: 0.09% Sodium azide<br>Constituents: 1.815% Tris, 1.764% Sodium citrate, 0.021% PBS
**Purity**	Immunogen affinity purified
**Purification notes**	ab70303 was affinity purified using an epitope specific to CTCF immobilized on solid support.
**Clonality**	Polyclonal

Isotype IgG

Applications

The Abpromise guarantee Our Abpromise guarantee covers the use of ab70303 in the following tested applications.

The application notes include recommended starting dilutions; optimal dilutions/concentrations should be determined by the end user.

Application	Abreviews	Notes
IHC-Fr	★★★★★ (1)	Use at an assay dependent concentration.
WB	★★★★☆ (5)	1/2500 - 1/10000.<br>Detects a band of approximately 100 kDa (predicted molecular weight: 83 kDa).
IP		Use at 2-5 µg/mg of lysate.
IHC-P	★☆☆☆☆ (1)	1/100 - 1/500. Perform heat mediated antigen retrieval with Tris/EDTA buffer pH 9.0 before commencing with IHC staining protocol.

Target

Function Chromatin binding factor that binds to DNA sequence specific sites. Involved in transcriptional regulation by binding to chromatin insulators and preventing interaction between promoter and nearby enhancers and silencers. Acts as transcriptional repressor binding to promoters of vertebrate MYC gene and BAG1 gene. Also binds to the PLK and PIM1 promoters. Acts as a transcriptional activator of APP. Regulates APOA1/C3/A4/A5 gene cluster and controls MHC class II gene expression. Plays an essential role in oocyte and preimplantation embryo development by activating or repressing transcription. Seems to act as tumor suppressor. Plays a critical role in the epigenetic regulation. Participates to the allele-specific gene expression at the imprinted IGF2/H19 gene locus. On the maternal allele, binding within the H19 imprinting control region (ICR) mediates maternally inherited higher-order chromatin conformation to restrict enhancer access to IGF2. Plays a critical role in gene silencing over considerable distances in the genome. Preferentially interacts with unmethylated DNA, preventing spreading of CpG methylation and maintaining methylation-free zones. Inversely, binding to target sites is prevented by CpG methylation. Plays a important role in chromatin remodeling. Can dimerize when it is bound to different DNA sequences, mediating long-range chromatin looping. Mediates interchromosomal association between IGF2/H19 and WSB1/NF1 and may direct distant DNA segments to a common transcription factory. Causes local loss of histone acetylation and gain of histone methylation in the beta-globin locus, without affecting transcription. When bound to chromatin, it provides an anchor point for nucleosomes positioning. Seems to be essential for homologous X-chromosome pairing. May participate with Tsix in establishing a regulatable epigenetic switch for X chromosome inactivation. May play a role in preventing the propagation of stable methylation at the escape genes from X- inactivation. Involved in sister chromatid cohesion. Associates with both centromeres and chromosomal arms during metaphase and required for cohesin localization to CTCF sites. Regulates asynchronous replication of IGF2/H19.

Tissue specificity Ubiquitous. Absent in primary spermatocytes.

Sequence similarities Belongs to the CTCF zinc-finger protein family.

	Contains 11 C2H2-type zinc fingers.
**Domain**	The 11 zinc fingers are highly conserved among vertebrates, exhibiting almost identical amino acid sequences. Different subsets or combination of individual zinc fingers gives the ability to CTCF to recognize multiple DNA target sites.
**Post-translational modifications**	Sumoylated on Lys-74 and Lys-689; sumoylation of CTCF contributes to the repressive function of CTCF on the MYC P2 promoter.
**Cellular localization**	Nucleus > nucleoplasm. Chromosome. Chromosome > centromere. May translocate to the nucleolus upon cell differentiation. Associates with both centromeres and chromosomal arms during metaphase. Associates with the H19 ICR in mitotic chromosomes. May be preferentially excluded from heterochromatin during interphase.

Images

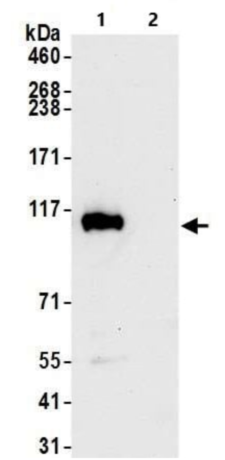

Immunoprecipitation - Anti-CTCF antibody (ab70303)

CTCF was immunoprecipitated from 1 mg Jurkat whole cell lysate with ab70303 at 6 µg per reaction. Western blot was performed on the immunoprecipitate using ab70303 at 0.02 µg/mL.

Lane 1: ab70303 IP in Jurkat whole cell lysate.

Lane 2: Contol IgG in Jurkat whole cell lysate.

Detection: Chemiluminescence.

Exposure time: 10 seconds.

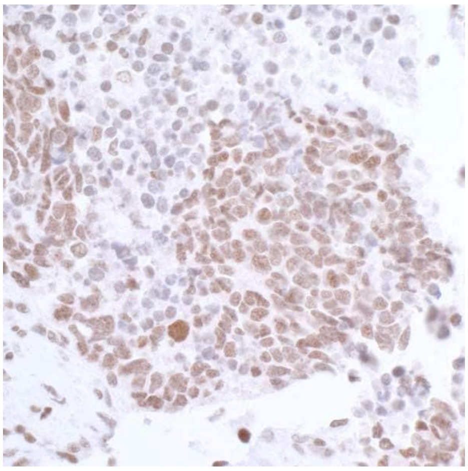

Immunohistochemistry (Formalin/PFA-fixed paraffin-embedded sections) - Anti-CTCF antibody (ab70303)

Immunohistochemical analysis of formalin-fixed, paraffin-embedded human small cell lung cancer tissue, labeling CTCF with ab70303 at a 1/1000 dilution. HRP-conjugated goat anti-rabbit IgG was used as a secondary antibody.

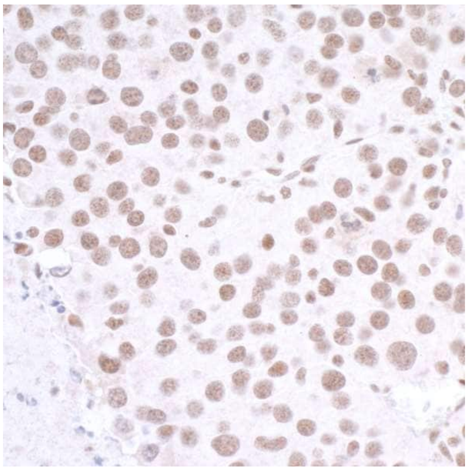

Immunohistochemistry (Formalin/PFA-fixed paraffin-embedded sections) - Anti-CTCF antibody (ab70303)

Immunohistochemical analysis of formalin-fixed, paraffin-embedded mouse renal cancer tissue, labeling CTCF with ab70303 at a 1/1000 dilution. HRP-conjugated goat anti-rabbit IgG was used as a secondary antibody.

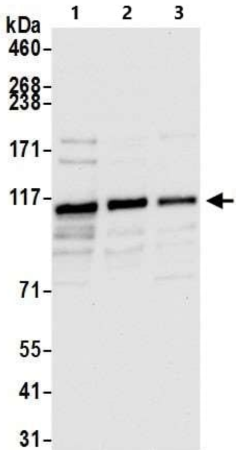

Western blot - Anti-CTCF antibody (ab70303)

All lanes : Anti-CTCF antibody (ab70303) at 0.02 µg/ml

Lane 1 : Jurkat whole cell lysate

Lane 2 : HEK293T whole cell lysate

Lane 3 : HeLa whole cell lysate

Lysates/proteins at 50 µg per lane.

Exposure time: 10 seconds

Detection: Chemiluminescence.

Lysates prepared using NETN lysis buffer.

Please note: All products are "FOR RESEARCH USE ONLY. NOT FOR USE IN DIAGNOSTIC PROCEDURES"

Our Abpromise to you: Quality guaranteed and expert technical support

- Replacement or refund for products not performing as stated on the datasheet
- Valid for 12 months from date of delivery
- Response to your inquiry within 24 hours

- We provide support in Chinese, English, French, German, Japanese and Spanish
- Extensive multi-media technical resources to help you
- We investigate all quality concerns to ensure our products perform to the highest standards

If the product does not perform as described on this datasheet, we will offer a refund or replacement. For full details of the Abpromise, please visit https://www.abcam.com/abpromise or contact our technical team.

Terms and conditions

- Guarantee only valid for products bought direct from Abcam or one of our authorized distributors

abcam

Product datasheet

Anti-Histone H3 (acetyl K27) antibody - ChIP Grade ab4729

★★★★★ 81 Abreviews 1539 References 10 Images

Overview

**Product name**	Anti-Histone H3 (acetyl K27) antibody - ChIP Grade
**Description**	Rabbit polyclonal to Histone H3 (acetyl K27) - ChIP Grade
**Host species**	Rabbit
**Tested applications**	**Suitable for:** ICC/IF, WB, IHC-P, ChIP, PepArr
**Species reactivity**	**Reacts with:** Mouse, Rat, Cow, Human, Recombinant fragment<br>**Predicted to work with:** Chicken, Xenopus laevis, Arabidopsis thaliana, Drosophila melanogaster, Monkey, Zebrafish, Plasmodium falciparum, Rice, Cyanidioschyzon merolae
**Immunogen**	Synthetic peptide corresponding to Human Histone H3 aa 1-100 (acetyl K27) conjugated to keyhole limpet haemocyanin.<br>(Peptide available as ab24404)
**Positive control**	WB : HeLa (human cervix adenocarcinoma epithelial cell) cell lysate - Sodium butyrate-treated, HeLa (human cervix adenocarcinoma epithelial cell) nuclear lysate (triton enriched), NIH/3T3 (mouse embryonic fibroblast cell line) nuclear lysate (triton enriched) and PC-12 (rat adrenal gland pheochromocytoma cell) nuclear lysate (triton enriched).
**General notes**	Learn about ChIP assay kits, other ChIP antibodies, protocols and more in the ChIP assay guide.<br><br>The Life Science industry has been in the grips of a reproducibility crisis for a number of years. Abcam is leading the way in addressing this with our range of recombinant monoclonal antibodies and knockout edited cell lines for gold-standard validation. Please check that this product meets your needs before purchasing.<br><br>If you have any questions, special requirements or concerns, please send us an inquiry and/or contact our Support team ahead of purchase. Recommended alternatives for this product can be found below, along with publications, customer reviews and Q&As

Properties

**Form**	Liquid
**Storage instructions**	Shipped at 4°C. Store at +4°C short term (1-2 weeks). Upon delivery aliquot. Store at -20°C or -80°C. Avoid freeze / thaw cycle.
**Storage buffer**	pH: 7.40

Preservative: 0.02% Sodium azide
Constituent: PBS

Batches of this product that have a concentration < 1mg/ml may have BSA added as a stabilising agent. If you would like information about the formulation of a specific lot, please contact our scientific support team who will be happy to help.

Purity Immunogen affinity purified

Clonality Polyclonal

Isotype IgG

Applications

The Abpromise guarantee Our Abpromise guarantee covers the use of ab4729 in the following tested applications.

The application notes include recommended starting dilutions; optimal dilutions/concentrations should be determined by the end user.

Application	Abreviews	Notes
ICC/IF	★★★★★ (21)	Use a concentration of 0.5 µg/ml.<br>Can be used with paraformaldehyde- or methanol- fixed cells.
WB	★★★★★ (20)	Use a concentration of 1 µg/ml. Detects a band of approximately 17 kDa (predicted molecular weight: 15 kDa).<br>We recommend Goat Anti-Rabbit IgG H&L (HRP) (ab97051) secondary antibody.
IHC-P	★★★★★ (4)	Use a concentration of 1 µg/ml. Perform heat mediated antigen retrieval before commencing with IHC staining protocol.
ChIP	★★★★★ (27)	Use 2 µg for 25 µg of chromatin.<br>We recommend GAPDH positive control ChIP primer pair ab267832 as a positive control.
PepArr		Use a concentration of 0.2 - 0.02 µg/ml.

Target

Function Core component of nucleosome. Nucleosomes wrap and compact DNA into chromatin, limiting DNA accessibility to the cellular machineries which require DNA as a template. Histones thereby play a central role in transcription regulation, DNA repair, DNA replication and chromosomal stability. DNA accessibility is regulated via a complex set of post-translational modifications of histones, also called histone code, and nucleosome remodeling.

Sequence similarities Belongs to the histone H3 family.

Developmental stage Expressed during S phase, then expression strongly decreases as cell division slows down during the process of differentiation.

Post-translational modifications Acetylation is generally linked to gene activation. Acetylation on Lys-10 (H3K9ac) impairs methylation at Arg-9 (H3R8me2s). Acetylation on Lys-19 (H3K18ac) and Lys-24 (H3K24ac) favors methylation at Arg-18 (H3R17me).
Citrullination at Arg-9 (H3R8ci) and/or Arg-18 (H3R17ci) by PADI4 impairs methylation and

represses transcription.
Asymmetric dimethylation at Arg-18 (H3R17me2a) by CARM1 is linked to gene activation. Symmetric dimethylation at Arg-9 (H3R8me2s) by PRMT5 is linked to gene repression. Asymmetric dimethylation at Arg-3 (H3R2me2a) by PRMT6 is linked to gene repression and is mutually exclusive with H3 Lys-5 methylation (H3K4me2 and H3K4me3). H3R2me2a is present at the 3' of genes regardless of their transcription state and is enriched on inactive promoters, while it is absent on active promoters.
Methylation at Lys-5 (H3K4me), Lys-37 (H3K36me) and Lys-80 (H3K79me) are linked to gene activation. Methylation at Lys-5 (H3K4me) facilitates subsequent acetylation of H3 and H4. Methylation at Lys-80 (H3K79me) is associated with DNA double-strand break (DSB) responses and is a specific target for TP53BP1. Methylation at Lys-10 (H3K9me) and Lys-28 (H3K27me) are linked to gene repression. Methylation at Lys-10 (H3K9me) is a specific target for HP1 proteins (CBX1, CBX3 and CBX5) and prevents subsequent phosphorylation at Ser-11 (H3S10ph) and acetylation of H3 and H4. Methylation at Lys-5 (H3K4me) and Lys-80 (H3K79me) require preliminary monoubiquitination of H2B at 'Lys-120'. Methylation at Lys-10 (H3K9me) and Lys-28 (H3K27me) are enriched in inactive X chromosome chromatin.
Phosphorylated at Thr-4 (H3T3ph) by GSG2/haspin during prophase and dephosphorylated during anaphase. Phosphorylation at Ser-11 (H3S10ph) by AURKB is crucial for chromosome condensation and cell-cycle progression during mitosis and meiosis. In addition phosphorylation at Ser-11 (H3S10ph) by RPS6KA4 and RPS6KA5 is important during interphase because it enables the transcription of genes following external stimulation, like mitogens, stress, growth factors or UV irradiation and result in the activation of genes, such as c-fos and c-jun. Phosphorylation at Ser-11 (H3S10ph), which is linked to gene activation, prevents methylation at Lys-10 (H3K9me) but facilitates acetylation of H3 and H4. Phosphorylation at Ser-11 (H3S10ph) by AURKB mediates the dissociation of HP1 proteins (CBX1, CBX3 and CBX5) from heterochromatin. Phosphorylation at Ser-11 (H3S10ph) is also an essential regulatory mechanism for neoplastic cell transformation. Phosphorylated at Ser-29 (H3S28ph) by MLTK isoform 1, RPS6KA5 or AURKB during mitosis or upon ultraviolet B irradiation. Phosphorylation at Thr-7 (H3T6ph) by PRKCBB is a specific tag for epigenetic transcriptional activation that prevents demethylation of Lys-5 (H3K4me) by LSD1/KDM1A. At centromeres, specifically phosphorylated at Thr-12 (H3T11ph) from prophase to early anaphase, by DAPK3 and PKN1. Phosphorylation at Thr-12 (H3T11ph) by PKN1 is a specific tag for epigenetic transcriptional activation that promotes demethylation of Lys-10 (H3K9me) by KDM4C/JMJD2C. Phosphorylation at Tyr-42 (H3Y41ph) by JAK2 promotes exclusion of CBX5 (HP1 alpha) from chromatin.
Monoubiquitinated by RAG1 in lymphoid cells, monoubiquitination is required for V(D)J recombination (By similarity). Ubiquitinated by the CUL4-DDB-RBX1 complex in response to ultraviolet irradiation. This may weaken the interaction between histones and DNA and facilitate DNA accessibility to repair proteins.

Cellular localization Nucleus. Chromosome.

Images

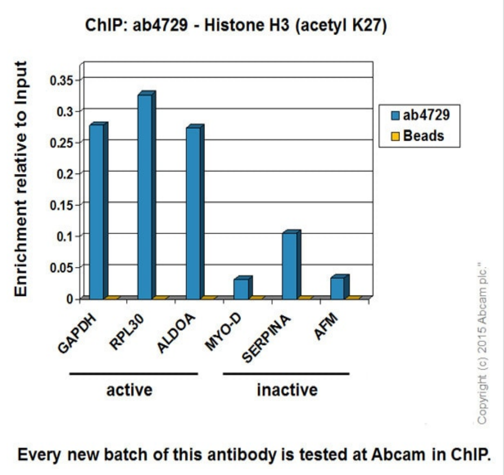

ChIP - Anti-Histone H3 (acetyl K27) antibody - ChIP Grade (ab4729)

Chromatin was prepared from HeLa (Human epithelial cell line from cervix adenocarcinoma) cells according to the Abcam X-ChIP protocol. Cells were fixed with formaldehyde for 10 minutes. The ChIP was performed with 25 µg of chromatin, 2 µg of ab4729 (blue), and 20 µl of Protein A/G sepharose beads.

No antibody was added to the beads control (yellow).

The immunoprecipitated DNA was quantified by real time PCR (Taqman approach). Primers and probes are located in the first kb of the transcribed region.

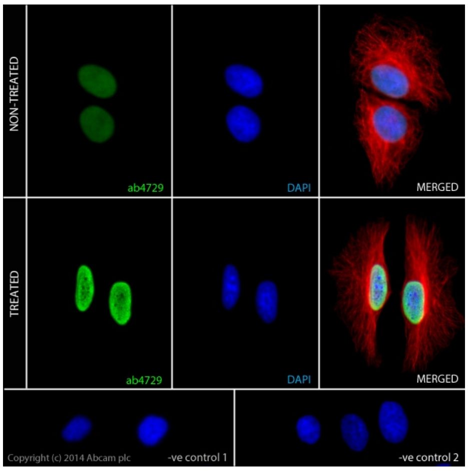

Immunocytochemistry/ Immunofluorescence - Anti-Histone H3 (acetyl K27) antibody - ChIP Grade (ab4729)

ab4729 staining Histone H3 (acetyl K27) in HeLa (Human epithelial cell line from cervix adenocarcinoma) cells.

The cells were incubated with 10 mM sodium butyrate (ab120948) for 6 hours (Treated) or solvent-only for control purposes (Non-treated). Cells were fixed with 100% methanol (5 minutes) and then blocked in 1% BSA/10% normal goat serum/0.3M glycine in 0.1%PBS-Tween for 1 hour. The cells were then incubated with ab4729 at 0.5 µg/ml and ab7291 at 1 µg/ml overnight at +4°C, followed by a further incubation at room temperature for 1 hour with a anti-rabbit AlexaFluor®488 secondary antibody (ab150077) at 2 µg/ml (shown in green) and a goat anti-mouse AlexaFluor®594 (ab150120) at 2 µg/ml (shown in pseudo colour red). Nuclear DNA was labeled in blue with DAPI.

Negative controls: 1– Rabbit primary and anti-mouse secondary antibody; 2 – Mouse primary antibody and anti-rabbit secondary antibody. Controls 1 and 2 indicate that there is no unspecific reaction between primary and secondary antibodies used.

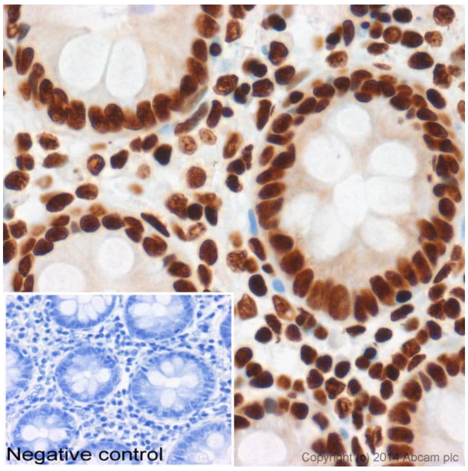

Immunohistochemistry (Formalin/PFA-fixed paraffin-embedded sections) - Anti-Histone H3 (acetyl K27) antibody - ChIP Grade (ab4729)

IHC image of ab4729 staining Histone H3 (acetyl K27) in human colon formalin-fixed paraffin-embedded tissue sections*, performed on a Leica Bond.

The section was pre-treated using heat mediated antigen retrieval with sodium citrate buffer pH 6 for 20 minutes. The section was then incubated with ab4729, 5 µg/ml, for 15 minutes at room temperature and detected using an HRP conjugated compact polymer system. DAB was used as the chromogen. The section was then counterstained with haematoxylin and mounted with DPX.

No primary antibody was used in the negative control (shown on the inset).

For other IHC staining systems (automated and non-automated) customers should optimize variable parameters such as antigen retrieval conditions, primary antibody concentration and antibody incubation times.

*Tissue obtained from the Human Research Tissue Bank, supported by the NIHR Cambridge Biomedical Research Centre

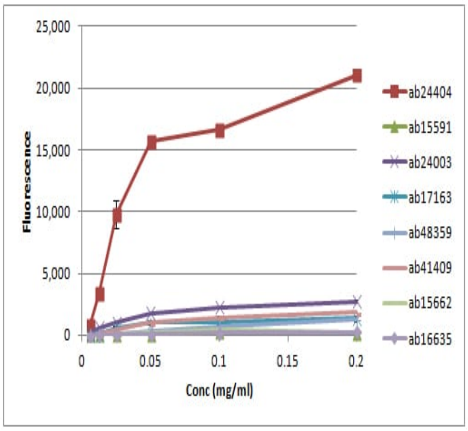

Peptide Array - Anti-Histone H3 (acetyl K27) antibody - ChIP Grade (ab4729)

All batches of ab4729 are tested in Peptide Array against peptides to different Histone H3 modifications. Six dilutions of each peptide are printed on to the Peptide Array in triplicate and results are averaged before being plotted on to a graph. Results show strong binding to Histone H3 - acetyl K27 peptide (ab24404), indicating that this antibody specifically recognises the Histone H3 - acetyl K27 modification.

ab24404 - Histone H3 - acetyl K27

ab15591 - Histone H3 - acetyl K14

ab24003 - Histone H3 - acetyl K18

ab17163 - Histone H3 unmodified

ab48359 - Histone H3 - acetyl K23

ab41409 - Histone H3 - acetyl K36

ab15662 - Histone H4 - acetyl K12

ab16635 - Histone H3 acetyl K9

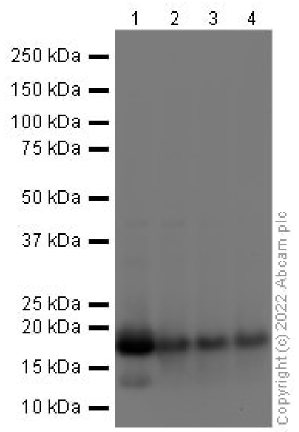

Western blot - Anti-Histone H3 (acetyl K27) antibody - ChIP Grade (ab4729)

All lanes : Anti-Histone H3 (acetyl K27) antibody - ChIP Grade (ab4729) at 1 µg/ml

Lane 1 : HeLa (human cervix adenocarcinoma epithelial cell) cell lysate - Sodium butyrate-treated
Lane 2 : HeLa (human cervix adenocarcinoma epithelial cell) nuclear lysate (triton enriched)
Lane 3 : NIH/3T3 (mouse embryonic fibroblast cell line) nuclear lysate (triton enriched)
Lane 4 : PC-12 (rat adrenal gland pheochromocytoma cell) nuclear lysate (triton enriched)

Lysates/proteins at 10 µg per lane.

Secondary
All lanes : Goat polyclonal to Rabbit IgG - H&L - Pre-Adsorbed (HRP) at 1/50000 dilution

Predicted band size: 15 kDa
Observed band size: 17 kDa

Exposure time: 30 seconds

Blocking buffer : 2% BSA block

Gel type : MES

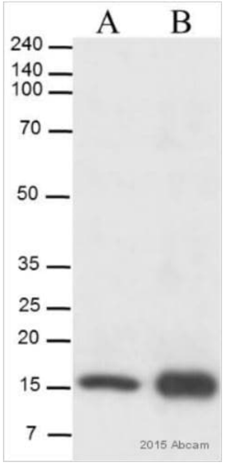

Western blot - Anti-Histone H3 (acetyl K27) antibody - ChIP Grade (ab4729)

All lanes : Anti-Histone H3 (acetyl K27) antibody - ChIP Grade (ab4729) at 1/2500 dilution

Lane 1 : Untreated Mouse MEF cell lysate
Lane 2 : 0.4 µM Trichostatin A treatment for 18 hr Mouse MEF cell lysate

Lysates/proteins at 9 µg per lane.

Secondary
All lanes : Donkey Anti-Rabbit IgG H&L (HRP) (ab6802) at 1/20000 dilution

Predicted band size: 15 kDa

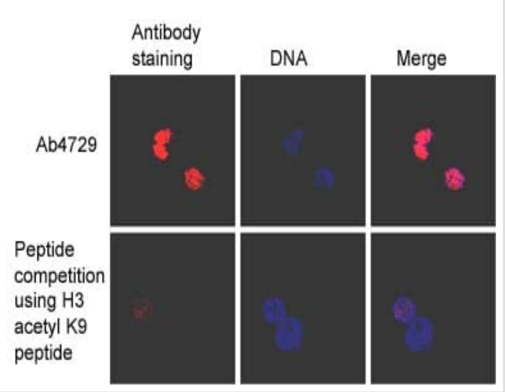

Immunocytochemistry/ Immunofluorescence - Anti-Histone H3 (acetyl K27) antibody - ChIP Grade (ab4729)

This image is courtesy of Petra Hajkova - Gurdon Institute, Cambridge University

Primary antibody: ab4729 (H3 acetyl K27)

Dilution: 1/100

ab4729 strongly stained histones of mouse ES cells. However, fluroescence was greatly diminished following pre-blocking using a H3 acetyl K9 peptide. This suggests the antibody cross-reacts with the K9 and K27 residues.

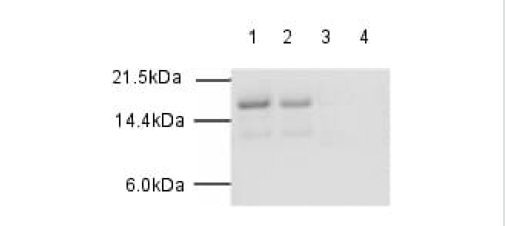

Western blot - Anti-Histone H3 (acetyl K27) antibody - ChIP Grade (ab4729)

Lanes 1 & 3 : Anti-Histone H3 (acetyl K27) antibody - ChIP Grade (ab4729) at 0.2 µg/ml

Lanes 2 & 4 : Anti-Histone H3 (acetyl K27) antibody - ChIP Grade (ab4729) at 0.1 µg/ml

Lanes 1-2 : Calf thymus histone lysate

Lanes 3-4 : Calf thymus histone lysate with Human Histone H3 (acetyl K27) peptide (ab24404) at 2 µg

Lysates/proteins at 1 µg per lane.

Secondary

All lanes : Goat anti-rabbit (HRP) at 1/2000 dilution

Predicted band size: 15 kDa

Observed band size: 17 kDa

ab4729 specifically recognises acetyl K27 histone H3 in catlf thymus histone lysate, which is specifically blocked using the immunizing peptide ab24404.

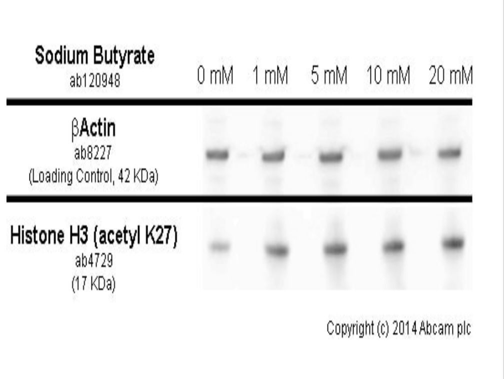

Western blot - Anti-Histone H3 (acetyl K27) antibody - ChIP Grade (ab4729)

HeLa (Human epithelial cell line from cervix adenocarcinoma) cells were incubated at 37°C for 6 hours with vehicle control (0 µM) and different concentrations of sodium butyrate (ab120948). Increased expression of histone H3 (acetyl K27)(ab4729) in HeLa cells correlates with an increase in sodium butyrate concentration, as described in literature.

Whole cell lysates were prepared with RIPA buffer (containing protease inhibitors and sodium orthovanadate), 2.5 µg of each were loaded on the gel and the WB was run under reducing conditions. After transfer the membrane was blocked for an hour using 5% BSA before being incubated with ab4927 at 1 µg/ml and ab8227 at 1 µg/ml overnight at 4°C. Antibody binding was detected using an anti-rabbit antibody conjugated to HRP (ab97051) at 1/10,000 dilution and visualised using ECL development solution.

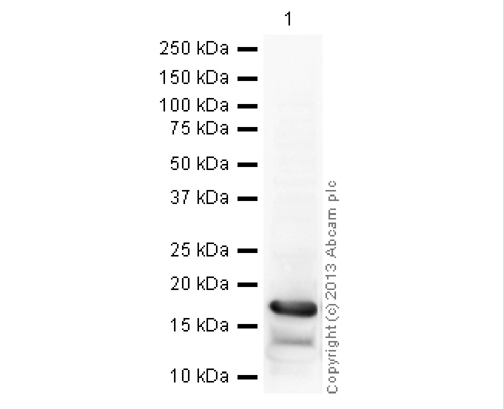

Western blot - Anti-Histone H3 (acetyl K27) antibody - ChIP Grade (ab4729)

Anti-Histone H3 (acetyl K27) antibody - ChIP Grade (ab4729) at 1 µg/ml + HeLa (Human epithelial cell line from cervix adenocarcinoma) histone preparation, nuclear Lysate - Butyrate treated at 2.5 µg

Secondary

Goat Anti-Rabbit IgG H&L (HRP) (ab97051) at 1/10000 dilution

Developed using the ECL technique.

Performed under reducing conditions.

Predicted band size: 15 kDa

Additional bands at: 17 kDa. We are unsure as to the identity of these extra bands.

Exposure time: 10 seconds

Please note: All products are "FOR RESEARCH USE ONLY. NOT FOR USE IN DIAGNOSTIC PROCEDURES"

Our Abpromise to you: Quality guaranteed and expert technical support

- Replacement or refund for products not performing as stated on the datasheet
- Valid for 12 months from date of delivery
- Response to your inquiry within 24 hours

- We provide support in Chinese, English, French, German, Japanese and Spanish
- Extensive multi-media technical resources to help you
- We investigate all quality concerns to ensure our products perform to the highest standards

If the product does not perform as described on this datasheet, we will offer a refund or replacement. For full details of the Abpromise, please visit https://www.abcam.com/abpromise or contact our technical team.

Terms and conditions

- Guarantee only valid for products bought direct from Abcam or one of our authorized distributors

Rec 4-20-06
Lot # 14790300?
Babis

COVANCE
THE DEVELOPMENT SERVICES COMPANY

RNA Polymerase II CTD4H8 Monoclonal Antibody, Alexa Fluor® Labeled
Catalog Number: A488-128L
Available Size: 0.1 mL

Description: Alexa Fluor® Labeled Monoclonal Antibody against RNA Polymerase II

Clone: CTD4H8

Form: Alexa Fluor® Labeled Antibody

Host: Mouse

IsoType: IgG1

Species Reactivity: Human, Yeast (S. cerevisiae)

Specificity: The antibody CTD4H8 recognizes the C-terminal repeat of the largest subunit of RNA polymerase II from HeLa and S. cerevisiae cells. This antibody recognizes both the phosphorylated and unphosphorylated forms of the RNA polymerase II.This immunogen used was a peptide containing 10 repeats of the synthetic peptide YSPTSPS using chemically synthesized phospho-ser5.

This antibody is covalently coupled to Alexa Fluor® 488 to generate a one-step staining reagent; no secondary antibody detection is required. Alexa Fluor® 488 is an excellent alternative to fluorescein (FITC) dyes.

Uses: This antibody is effective in immunoblotting, immunprecipitation and ELISA. This antibody can be used in chromatin immunoprecipitation assays.

Suggested Working Dilution: The optimal working dilution should be determined for each specific assay condition. *This antibody is sold for laboratory research use only, not for human or in-vivo use. Covance antibodies may not be resold or modified for resale without prior written approval.*

- Western blot: 1:500
- Immunofluorescence: 1:1,000

Notes: Alexa Fluor® is a trademark of Molecular Probes, Inc.

Storage: Store at -20°C. Upon initial thawing, apportion into working aliquots and store at -20°C. Avoid repeated freeze-thaw cycles to prevent denaturing the antibody.

This product is provided under an agreement with Molecular Probes, Inc. and the manufacture, use, sale or import of this product is subject to one or more of U.S. Patent Nos. 5,696,157 6,130,101, 5,132,432, 6,716,979, pending patent applications and corresponding foreign equivalents, owned by Invitrogen Corp. The purchase of this product conveys to the buyer the non-transferable right to use the purchased amount of the product and components of the product in research or research services conducted by the buyer (whether the buyer is an academic or for-profit entity), but excluding use in combination with microarrays or High Content Screening. The buyer cannot sell or otherwise transfer (a) this product (b) its components or (c) materials made using this product or its components to a third party or otherwise use this product or its components or materials made using this product or its components for Commercial Purposes, except to provide research services. Commercial Purposes means any activity by a party for consideration and may include, but is not limited to: (1) use of the product or its components in manufacturing; (2) use of the product or its components to provide a service, information, or data; (3) use of the product or its components for therapeutic, diagnostic or prophylactic purposes; or (4) resale of the product or its components, whether or not such product or its components are resold for use in research.

COVANCE
THE DEVELOPMENT SERVICES COMPANY

References: Kristjuhan A, Walker J, Suka N, Grunstein M, Roberts D, Cairns BR, Svejstrup JQ. Transcriptional inhibition of genes with severe histone h3 hypoacetylation in the coding region. *Mol. Cell. 10 (4)*:925-933, 2002.

Related Products:

- RNA Polymerase II CTD4H8 Monoclonal Antibody
  Catalog Number MMS-128P

SANTA CRUZ BIOTECHNOLOGY, INC.

Bcl-6 (D-8): sc-7388

The Power to Question

BACKGROUND

Bcl-6, a transcriptional repressor, binds Stat recognition-like DNA elements and influences germinal center development and Th1/Th2 differentiation. Bcl-6 negatively regulates NFκB expression, thereby inhibiting NFκB-mediated cellular functions. HDAC- and silent information regulator (SIR)-2-dependent acetylation of Bcl-6 causes downregulation of activity by inhibiting the ability of Bcl-6 to recruit complexes containing histone deacetylases (HDACs). Bcl-6 is frequently deregulated in non-Hodgkin's B cell lymphomas. The human Bcl-6 gene has been shown to encode a protein of 706 amino acids.

CHROMOSOMAL LOCATION

Genetic locus: BCL6 (human) mapping to 3q27.3; Bcl6 (mouse) mapping to 16 B1.

SOURCE

Bcl-6 (D-8) is a mouse monoclonal antibody raised against amino acids 3-484 mapping at the N-terminus of Bcl-6 of human origin.

PRODUCT

Each vial contains 200 µg IgG_3 kappa light chain in 1.0 ml of PBS with < 0.1% sodium azide and 0.1% gelatin. Also available as TransCruz reagent for Gel Supershift and ChIP applications, sc-7388 X, 200 µg/0.1 ml.

Bcl-6 (D-8) is available conjugated to agarose (sc-7388 AC), 500 µg/0.25 ml agarose in 1 ml, for IP; to HRP (sc-7388 HRP), 200 µg/ml, for WB, IHC(P) and ELISA; and to either phycoerythrin (sc-7388 PE), fluorescein (sc-7388 FITC) or Alexa Fluor® 488 (sc-7388 AF488) or Alexa Fluor® 647 (sc-7388 AF647), 200 µg/ml, for WB (RGB), IF, IHC(P) and FCM.

Alexa Fluor® is a trademark of Molecular Probes, Inc., Oregon, USA

APPLICATIONS

Bcl-6 (D-8) is recommended for detection of Bcl-6 of mouse, rat and human origin by Western Blotting (starting dilution 1:200, dilution range 1:100-1:1000), immunoprecipitation [1-2 µg per 100-500 µg of total protein (1 ml of cell lysate)], immunofluorescence (starting dilution 1:50, dilution range 1:50-1:500) and immunohistochemistry (including paraffin-embedded sections) (starting dilution 1:50, dilution range 1:50-1:500).

Suitable for use as control antibody for Bcl-6 siRNA (h): sc-29791, Bcl-6 siRNA (m): sc-29792, Bcl-6 shRNA Plasmid (h): sc-29791-SH, Bcl-6 shRNA Plasmid (m): sc-29792-SH, Bcl-6 shRNA (h) Lentiviral Particles: sc-29791-V and Bcl-6 shRNA (m) Lentiviral Particles: sc-29792-V.

Bcl-6 (D-8) X TransCruz antibody is recommended for Gel Supershift and ChIP applications.

Molecular Weight of Bcl-6: 95 kDa.

Positive Controls: BJAB whole cell lysate: sc-2207, Raji whole cell lysate: sc-364236 or Ramos cell lysate: sc-2216.

STORAGE

Store at 4° C, **DO NOT FREEZE**. Stable for one year from the date of shipment. Non-hazardous. No MSDS required.

RESEARCH USE

For research use only, not for use in diagnostic procedures.

DATA

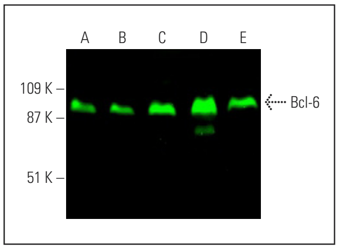

Bcl-6 (D-8): sc-7388. Near-infrared western blot analysis of Bcl-6 expression in Ramos (**A**), U-698-M (**B**), Raji (**C**), BJAB (**D**) and NAMALWA (**E**) whole cell lysates. Blocked with UltraCruz® Blocking Reagent: sc-516214. Detection reagent used: m-IgGκ BP-CFL 680: sc-516180.

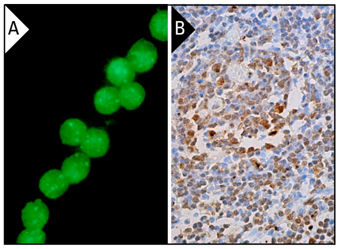

Bcl-6 (D-8): sc-7388. Immunofluorescence staining of methanol-fixed BJAB cells showing nuclear localization (**A**). Bcl-6 (D-8) HRP:sc-7388 HRP. Direct immunoperoxidase staining of formalin fixed, paraffin-embedded human lymph node tissue showing nuclear staining of cells in germinal center and cells in non-germinal center. Blocked with 0.25X UltraCruz® Blocking Reagent: sc-516214 (**B**).

SELECT PRODUCT CITATIONS

1. Dong, C., et al. 1998. Defective T cell differentiation in the absence of Jnk1. Science 282: 2092-2095.
2. Toda, H., et al. 2013. Clinicopathologic analysis of localized nasal/paranasal diffuse large B-cell lymphoma. PLoS ONE 8: e57677.
3. Schmitt, N., et al. 2014. The cytokine TGF-β co-opts signaling via Stat3-Stat4 to promote the differentiation of human TFH cells. Nat. Immunol. 15: 856-865.
4. Xu, Y., et al. 2015. Loss of IRF8 inhibits the growth of diffuse large B-cell lymphoma. J. Cancer 6: 953-961.
5. Muschol-Steinmetz, C., et al. 2016. B-cell lymphoma 6 promotes proliferation and survival of trophoblastic cells. Cell Cycle 15: 827-839.
6. Noujima-Harada, M., et al. 2017. Frequent downregulation of BTB and CNC homology 2 expression in Epstein-Barr virus-positive diffuse large B-cell lymphoma. Cancer Sci. 108: 1071-1079.
7. Li, Y., et al. 2018. MiR-339-5p inhibits metastasis of non-small cell lung cancer by regulating the epithelial-to-mesenchymal transition. Oncol. Lett. 15: 2508-2514.
8. Sommars, M.A., et al. 2019. Dynamic repression by Bcl-6 controls the genome-wide liver response to fasting and steatosis. Elife 8: e43922.
9. Fabre, M.S., et al. 2020. The oncogene BCL6 is up-regulated in glioblastoma in response to DNA damage, and drives survival after therapy. PLoS ONE 15: e0231470.
10. Bolognesi, M.M., et al. 2021. Antibodies validated for routinely processed tissues stain frozen sections unpredictably. BioTechniques 70: 137-148.

PROTOCOLS

See our web site at www.scbt.com for detailed protocols and support products.

Bcl-6 (D-8): sc-7388

SANTA CRUZ BIOTECHNOLOGY
The Power to Question

BACKGROUND

Bcl-6, a transcriptional repressor, binds Stat recognition-like DNA elements and influences germinal center development and Th1/Th2 differentiation. Bcl-6 negatively regulates NFκB expression, thereby inhibiting NFκB-mediated cellular functions. HDAC- and silent information regulator (SIR)-2-dependent acetylation of Bcl-6 causes downregulation of activity by inhibiting the ability of Bcl-6 to recruit complexes containing histone deacetylases (HDACs). Bcl-6 is frequently deregulated in non-Hodgkin's B cell lymphomas. The human Bcl-6 gene has been shown to encode a protein of 706 amino acids.

CHROMOSOMAL LOCATION

Genetic locus: BCL6 (human) mapping to 3q27.3; Bcl6 (mouse) mapping to 16 B1.

SOURCE

Bcl-6 (D-8) is a mouse monoclonal antibody raised against amino acids 3-484 mapping at the N-terminus of Bcl-6 of human origin.

PRODUCT

Each vial contains 200 µg IgG_3 kappa light chain in 1.0 ml of PBS with < 0.1% sodium azide and 0.1% gelatin. Also available as TransCruz reagent for Gel Supershift and ChIP applications, sc-7388 X, 200 µg/0.1 ml.

Bcl-6 (D-8) is available conjugated to agarose (sc-7388 AC), 500 µg/0.25 ml agarose in 1 ml, for IP; to HRP (sc-7388 HRP), 200 µg/ml, for WB, IHC(P) and ELISA; and to either phycoerythrin (sc-7388 PE), fluorescein (sc-7388 FITC) or Alexa Fluor® 488 (sc-7388 AF488) or Alexa Fluor® 647 (sc-7388 AF647), 200 µg/ml, for WB (RGB), IF, IHC(P) and FCM.

Alexa Fluor® is a trademark of Molecular Probes, Inc., Oregon, USA

APPLICATIONS

Bcl-6 (D-8) is recommended for detection of Bcl-6 of mouse, rat and human origin by Western Blotting (starting dilution 1:200, dilution range 1:100-1:1000), immunoprecipitation [1-2 µg per 100-500 µg of total protein (1 ml of cell lysate)], immunofluorescence (starting dilution 1:50, dilution range 1:50-1:500) and immunohistochemistry (including paraffin-embedded sections) (starting dilution 1:50, dilution range 1:50-1:500).

Suitable for use as control antibody for Bcl-6 siRNA (h): sc-29791, Bcl-6 siRNA (m): sc-29792, Bcl-6 shRNA Plasmid (h): sc-29791-SH, Bcl-6 shRNA Plasmid (m): sc-29792-SH, Bcl-6 shRNA (h) Lentiviral Particles: sc-29791-V and Bcl-6 shRNA (m) Lentiviral Particles: sc-29792-V.

Bcl-6 (D-8) X TransCruz antibody is recommended for Gel Supershift and ChIP applications.

Molecular Weight of Bcl-6: 95 kDa.

Positive Controls: BJAB whole cell lysate: sc-2207, Raji whole cell lysate: sc-364236 or Ramos cell lysate: sc-2216.

STORAGE

Store at 4° C, **DO NOT FREEZE**. Stable for one year from the date of shipment. Non-hazardous. No MSDS required.

RESEARCH USE

For research use only, not for use in diagnostic procedures.

DATA

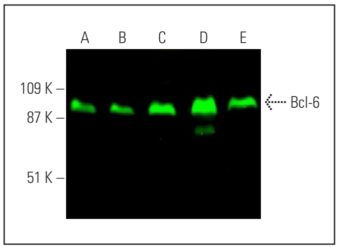

Bcl-6 (D-8): sc-7388. Near-infrared western blot analysis of Bcl-6 expression in Ramos (**A**), U-698-M (**B**), Raji (**C**), BJAB (**D**) and NAMALWA (**E**) whole cell lysates. Blocked with UltraCruz® Blocking Reagent: sc-516214. Detection reagent used: m-IgGκ BP-CFL 680: sc-516180.

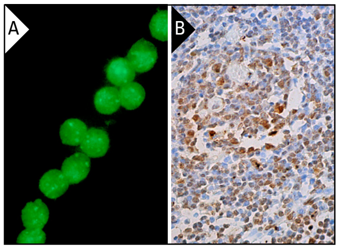

Bcl-6 (D-8): sc-7388. Immunofluorescence staining of methanol-fixed BJAB cells showing nuclear localization (**A**). Bcl-6 (D-8) HRP:sc-7388 HRP. Direct immunoperoxidase staining of formalin fixed, paraffin-embedded human lymph node tissue showing nuclear staining of cells in germinal center and cells in non-germinal center. Blocked with 0.25X UltraCruz® Blocking Reagent: sc-516214 (**B**).

SELECT PRODUCT CITATIONS

1. Dong, C., et al. 1998. Defective T cell differentiation in the absence of Jnk1. Science 282: 2092-2095.
2. Toda, H., et al. 2013. Clinicopathologic analysis of localized nasal/paranasal diffuse large B-cell lymphoma. PLoS ONE 8: e57677.
3. Schmitt, N., et al. 2014. The cytokine TGF-β co-opts signaling via Stat3-Stat4 to promote the differentiation of human TFH cells. Nat. Immunol. 15: 856-865.
4. Xu, Y., et al. 2015. Loss of IRF8 inhibits the growth of diffuse large B-cell lymphoma. J. Cancer 6: 953-961.
5. Muschol-Steinmetz, C., et al. 2016. B-cell lymphoma 6 promotes proliferation and survival of trophoblastic cells. Cell Cycle 15: 827-839.
6. Noujima-Harada, M., et al. 2017. Frequent downregulation of BTB and CNC homology 2 expression in Epstein-Barr virus-positive diffuse large B-cell lymphoma. Cancer Sci. 108: 1071-1079.
7. Li, Y., et al. 2018. MiR-339-5p inhibits metastasis of non-small cell lung cancer by regulating the epithelial-to-mesenchymal transition. Oncol. Lett. 15: 2508-2514.
8. Sommars, M.A., et al. 2019. Dynamic repression by Bcl-6 controls the genome-wide liver response to fasting and steatosis. Elife 8: e43922.
9. Fabre, M.S., et al. 2020. The oncogene BCL6 is up-regulated in glioblastoma in response to DNA damage, and drives survival after therapy. PLoS ONE 15: e0231470.
10. Bolognesi, M.M., et al. 2021. Antibodies validated for routinely processed tissues stain frozen sections unpredictably. BioTechniques 70: 137-148.

PROTOCOLS

See our web site at www.scbt.com for detailed protocols and support products.

For Research Use Only

RAG2 Polyclonal antibody

Catalog Number: 11825-1-AP   1 Publications

proteintech® Antibodies | ELISA kits | Proteins
www.ptglab.com

Basic Information

Catalog Number:
11825-1-AP

Size:
150ul , Concentration: 200 μg/ml by Nanodrop and 193 μg/ml by Bradford method using BSA as the standard;

Source:
Rabbit

Isotype:
IgG

Immunogen Catalog Number:
AG2393

GenBank Accession Number:
BC022397

GeneID (NCBI):
5897

Full Name:
recombination activating gene 2

Calculated MW:
527 aa, 59 kDa

Observed MW:
57-62 kDa

Purification Method:
Antigen affinity purification

Recommended Dilutions:
WB 1:200-1:1000
IP 0.5-4.0 ug for IP and 1:200-1:1000 for WB
IHC 1:20-1:200
IF 1:10-1:100

Applications

Tested Applications:
FC, IF, IHC, IP, WB, ELISA

Cited Applications:
WB

Species Specificity:
human, mouse

Cited Species:
mouse

Note-IHC: suggested antigen retrieval with TE buffer pH 9.0; (*) Alternatively, antigen retrieval may be performed with citrate buffer pH 6.0

Positive Controls:

WB : A375 cells, mouse thymus tissue

IP : A375 cells,

IHC : human lymphoma tissue,

IF : Hela cells,

Background Information

Recombination activating gene 2(RAG2) is core part of the RAG complex(RAG1 and RAG2), which mediates the DNA cleavage phase during V(D)J recombination. The RAG complex also plays a role in pre-B cell allelic exclusion, a process leading to expression of a single immunoglobulin heavy chain allele to enforce clonality and monospecific recognition by the B-cell antigen receptor (BCR) expressed on individual B-lymphocytes. The introduction of DNA breaks by the RAG complex on one immunoglobulin allele induces ATM-dependent repositioning of the other allele to pericentromeric heterochromatin, preventing accessibility to the RAG complex and recombination of the second allele. In the RAG complex, RAG2 is not the catalytic component but is required for all known catalytic activities mediated by RAG1. It probably acts as a sensor of chromatin state that recruits the RAG complex to H3K4me3

Notable Publications

Author	Pubmed ID	Journal	Application
Jannek Hauser	24470503	J Immunol	WB

Storage

Storage:
Store at -20°C. Stable for one year after shipment.
Storage Buffer:
PBS with 0.02% sodium azide and 50% glycerol pH 7.3.
Aliquoting is unnecessary for -20°C storage

For technical support and original validation data for this product please contact:
T: 1 (888) 4PTGLAB (1-888-478-4522) (toll free in USA), or 1(312) 455-8498 (outside USA)
E: proteintech@ptglab.com
W: ptglab.com

This product is exclusively available under Proteintech Group brand and is not available to purchase from any other manufacturer.

Selected Validation Data

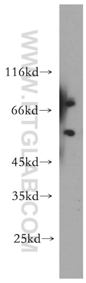

A375 cells were subjected to SDS PAGE followed by western blot with 11825-1-AP (RAG2 antibody) at dilution of 1:300 incubated at room temperature for 1.5 hours.

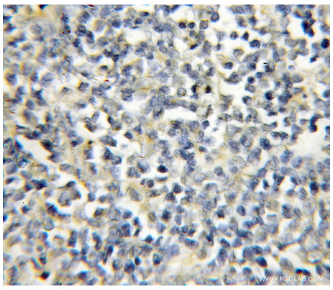

Immunohistochemical analysis of paraffin-embedded human lymphoma using 11825-1-AP (RAG2 antibody) at dilution of 1:50 (under 10x lens).

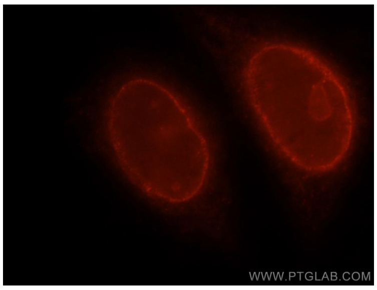

Immunofluorescent analysis of Hela cells, using RAG2 antibody 11825-1-AP at 1:25 dilution and Rhodamine-labeled goat anti-rabbit IgG (red).

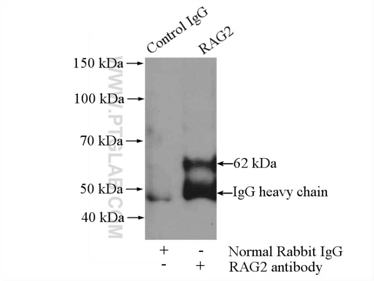

IP Result of anti-RAG2 (IP:11825-1-AP, 4ug; Detection:11825-1-AP 1:300) with A375 cells lysate 3600ug.

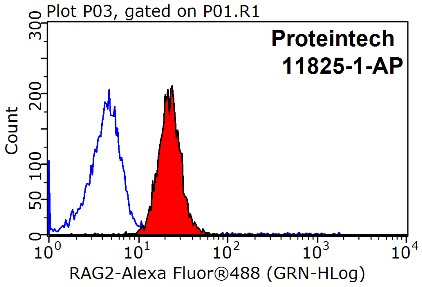

1X10^6 HeLa cells were stained with 0.2ug RAG2 antibody (11825-1-AP, red) and control antibody (blue). Fixed with 90% MeOH blocked with 3% BSA (30 min). Alexa Fluor 488-conjugated AffiniPure Goat Anti-Rabbit IgG(H+L) with dilution 1:1000.
