# Supplementary material for: The 3D enhancer network of the developing T cell genome is shaped by SATB1
Source: Nat Commun. 2022 Nov 14;13:6954. doi: 10.1038/s41467-022-34345-y (PMC9663569; doi:10.1038/s41467-022-34345-y)
Supplement: Supplementary file 13 — Reporting Summary [file 41467_2022_34345_MOESM13_ESM.pdf]

Corresponding author(s): Charalampos Spilianakis

Last updated by author(s): Sep 23, 2022

Reporting Summary

Nature Portfolio wishes to improve the reproducibility of the work that we publish. This form provides structure for consistency and transparency in reporting. For further information on Nature Portfolio policies, see our Editorial Policies and the Editorial Policy Checklist.

Statistics

For all statistical analyses, confirm that the following items are present in the figure legend, table legend, main text, or Methods section.

n/a	Confirmed	
☐	☒	The exact sample size (n) for each experimental group/condition, given as a discrete number and unit of measurement
☐	☒	A statement on whether measurements were taken from distinct samples or whether the same sample was measured repeatedly
☐	☒	The statistical test(s) used AND whether they are one- or two-sided<br>*Only common tests should be described solely by name; describe more complex techniques in the Methods section.*
☐	☒	A description of all covariates tested
☐	☒	A description of any assumptions or corrections, such as tests of normality and adjustment for multiple comparisons
☐	☒	A full description of the statistical parameters including central tendency (e.g. means) or other basic estimates (e.g. regression coefficient) AND variation (e.g. standard deviation) or associated estimates of uncertainty (e.g. confidence intervals)
☐	☒	For null hypothesis testing, the test statistic (e.g. F, t, r) with confidence intervals, effect sizes, degrees of freedom and P value noted<br>*Give P values as exact values whenever suitable.*
☒	☐	For Bayesian analysis, information on the choice of priors and Markov chain Monte Carlo settings
☒	☐	For hierarchical and complex designs, identification of the appropriate level for tests and full reporting of outcomes
☐	☒	Estimates of effect sizes (e.g. Cohen's d, Pearson's r), indicating how they were calculated

Our web collection on statistics for biologists contains articles on many of the points above.

Software and code

Policy information about availability of computer code

Data collection	No software was used.
Data analysis	No special software was developed and/or used in the study. The following software was used: bowtie2 (version 2.3.4.3+2.4.2; Langmead and Salzberg, 2012), HiC-Pro (version 2.11.1; Servant et al., 2015), FitHiChIP (version 6.0; Bhattacharyya et al., 2019), MACS2 (version 2.1.2), Samtools (version 1.9; Li et al., 2009), Juicer Tools (version 1.8.9; Durand et al., 2016), HISAT2 (version 2.1.0; Kim et al., 2019), Bamtools (version 2.4.1; Barnett et al., 2011), StringTie (version 1.3.4d; Pertea et al., 2015), featureCounts (version 1.6.2; Liao et al., 2014), DESeq2 (1.18.1; Love et al., 2014), esATAC pipeline (version 1.4.2; Wei et al., 2018), F-seq (Boyle et al., 2008), Rsubread package (version 1.32.4; Liao et al., 2019), edgeR (version 3.24.3; Robinson et al., 2010), CrossMap (version 0.2.7; Zhao et al., 2014), NucleoATAC (version 0.3.4; Schep et al., 2015), MEME (version 5.1.1; Bailey and Elkan, 1994), hicexplorer (version 3.5.1; Ramírez et al., 2018), TADbit (Serra et al., 2017), MCL Markov clustering (Enright et al., 2002), UCSF Chimera (Pettersen et al., 2004), LorDG (Trieu and Cheng, 2017), skinny-dip test (Maurus and Plant, 2016), Calibur (Li and Ng, 2010), 3DMax (Oluwadare et al., 2018), gProfileR (version 0.7.0; Reimand et al., 2007), bedtools (version 2.27.1; Quinlan and Hall, 2010), Fiji (version 1.53f51; Schindelin et al., 2012), Picard MarkDuplicates (http://broadinstitute.github.io/picard/), deepTools (version 3.1.3; Ramírez et al., 2016), KR balancing method (Knight and Ruiz, 2013), Homer (version 4.10.3; Heinz et al., 2010), GENOVA (version 1.0.0.9000; van der Weide et al., 2021), ICE normalization (Imakaev et al., 2012), TADCompare (version 1.4.0; Cresswell and Dozmorov, 2020), LEGENDplex 8.0 (Biolegend, 740741), FlowJo v10.8.0, FCSalyzer (version 0.9.17; https://sourceforge.net/projects/fcsalyzer), Harmony 4.1, Sigma Plot 12, Relative Distance metric (Favorov et al., 2012), SIPMeta (version 1.3.1; Rowley et al., 2020).

For manuscripts utilizing custom algorithms or software that are central to the research but not yet described in published literature, software must be made available to editors and reviewers. We strongly encourage code deposition in a community repository (e.g. GitHub). See the Nature Portfolio guidelines for submitting code & software for further information.

Data

Policy information about availability of data

All manuscripts must include a data availability statement. This statement should provide the following information, where applicable:

- Accession codes, unique identifiers, or web links for publicly available datasets
- A description of any restrictions on data availability
- For clinical datasets or third party data, please ensure that the statement adheres to our policy

The source data underlying all figures is provided as a Source Data File. Raw and processed data for all genomics experiments are deposited in Gene Expression Omnibus database under accession number GSE173476. We re-analyzed publicly available Hi-C datasets from untreated and CTCF-depleted mESCs (https://doi.org/10.1016/j.cell.2017.05.004) and WT and RAD21-depleted DP murine thymocytes (https://doi.org/10.1101/gr.161620.113). We re-analyzed publicly available RNA-seq of sorted DN1, DN2b, DN3, DN4, ISP, DP, CD4SP, CD8SP immune cell populations (GSE109125; https://doi.org/10.1016/j.cell.2018.12.036). Publicly available ChIP-seq datasets were: SATB1 ChIP-seq (GSM1617950; Hao et al., 2015), (DRR061108; Kitagawa et al., 2017), CTCF ChIP-seq (ENCFF714WDP; Dunham et al., 2012), H3K4me3 ChIP-seq (ENCFF200ISF; Davis et al., 2018), H3K4me1 ChIP-seq (ENCFF085AXD; Davis et al., 2018), H3K27ac ChIP-seq in WT (GSM1504384+5) and Rad21 cKO (GSM1504386+7) DP cells (Ing-Simmons et al., 2015), thymus-specific enhancers (GSE29184; Shen et al., 2012). Additionally, ChIP-Atlas (Oki et al., 2018) was used in the pilot analysis. All the other datasets generated and/or analyzed during the current study are available from the corresponding author upon request.

Field-specific reporting

Please select the one below that is the best fit for your research. If you are not sure, read the appropriate sections before making your selection.

☒ Life sciences ☐ Behavioural & social sciences ☐ Ecological, evolutionary & environmental sciences

For a reference copy of the document with all sections, see nature.com/documents/nr-reporting-summary-flat.pdf

Life sciences study design

All studies must disclose on these points even when the disclosure is negative.

Sample size	No sample size calculation was performed as it does not directly apply to our type of study. For sequencing experiments, at least two independent samples were generated as per ENCODE guidelines (https://doi.org/10.1101/gr.136184.111). Other experiments were repeated sufficient times (3 times at least), such as clear and robust conclusions could be drawn.
Data exclusions	No data were excluded from genomics analyses. In scatter plots depicting the correlation between gene expression changes and changes in H3K27ac looping, the genes with linking score >|40| (overinteracting – underinteracting H3K27ac loops) were not included as influential outliers. The LEGENDplex (13-plex) Mouse Th Cytokine Panel V02 (Biolegend, 740741; Lot B289245) had faulty IL-21 measurements as confirmed by the manufacturer and thus it was excluded from the analysis. Moreover, one WT animal was not included in the LEGENDplex analysis as an influential outlier (>100 times higher concentrations compared to other WT animals). No other data were excluded from this study.
Replication	Due to highly demanding nature of high throughput 3C-based experiments, we performed only two biological replicates for each genotype for each SATB1 HiChIP, CTCF HiChIP, H3K27ac HiChIP and Hi-C. Both replicates were well correlated for each dataset, hence we combined them for the downstream analysis and visualization. Two replicates separately were used for certain applications to infer statistical significance (such as in the 3D modeling). We performed three biological replicates for each genotype for ATAC-seq and RNA-seq and these were used to calculate statistical significance of our observations. For flow cytometry experiments three or more animals were used for each genotype as indicated in the manuscript. Histology sections were also performed for at least three animals to validate the observed phenotype. Moreover, the validity of our claims was ensured by combining multiple research approaches reaching the same conclusion.
Randomization	Randomization was not relevant to this type of study. Given the type of this study, randomization was only performed in order to construct a null statistical model for genomics data processing.
Blinding	Blinding was not performed as the subjects of this study were cells and not individuals. The authors were aware of the condition or genotype of an analyzed subset of cells during the analysis.

Reporting for specific materials, systems and methods

We require information from authors about some types of materials, experimental systems and methods used in many studies. Here, indicate whether each material, system or method listed is relevant to your study. If you are not sure if a list item applies to your research, read the appropriate section before selecting a response.

Materials & experimental systems

n/a	Involved in the study
☐	☒ Antibodies
☒	☐ Eukaryotic cell lines
☒	☐ Palaeontology and archaeology
☐	☒ Animals and other organisms
☒	☐ Human research participants
☒	☐ Clinical data
☒	☐ Dual use research of concern

Methods

n/a	Involved in the study
☒	☐ ChIP-seq
☐	☒ Flow cytometry
☒	☐ MRI-based neuroimaging

Antibodies

Antibodies used	anti-Phosphatidylserine FITC conjugated (Biolegend, 640906, N.A., B170366), anti-CD4 PE conjugated (Pharmingen, 553730, GK1.5, 01186), anti-CD4 FITC conjugated (Biolegend, 100406, GK1.5, B171662), anti-CD4 PerCP conjugated (Biolegend, 100432, GK1.5, B164183), anti-CD8a APC conjugated (Biolegend, 100712, 53-6.7, B156591), anti-CD8a PE conjugated (Biolegend, 100708, 53-6.7, B155935), anti-CD44 PE conjugated (Pharmingen, 553134, IM7, 08306), anti-CD62L FITC conjugated (Biolegend, 104406, MEL-14, B157449), Alexa Fluor 594 Goat anti mouse IgG (H+L) (Invitrogen, A-11005, RRID: AB_2534091, 54432A), Alexa Fluor 594 (Invitrogen, A-11005, N.A., 54432-A), Alexa Fluor 647 (Invitrogen, A-21235, N.A., 51782-A), anti-SATB1 (Santa Cruz Biotechnology, sc-376096, C-6, K2719), anti-CTCF (Abcam, ab70303, N.A., GR320146-6), anti-H3K27ac (Abcam, ab4729, N.A., GR3251519-3, GR3251520-1), anti-RNA Polymerase II (Covance, A488-128L, CTD4H8, 17147909002), anti-BCL6 (Santa Cruz Biotechnology, sc-7388, D-8, G0621), anti-BCL6 (Santa Cruz Biotechnology, sc-550543, D-8, G0621), Anti-RAG2 (Proteintech, 11825-1-AP, N.A., N.A.), Anti-RAG1 (Abcam, ab172637, EPRAGR1, N.A.), β-Actin (ORIGENE, TA811000, OTI1, V0102)
Validation	Custom-made anti-SATB1 antibody was knock-out validated and compared in western blot and immunofluorescence microscopy experiments in this manuscript and also in our accompanying manuscript (https://doi.org/10.1101/2021.08.11.455932). Commercial antibodies were validated by the manufacturers as explained in their respective datasheets. All the validations are provided in Supplementary Data 9.

Animals and other organisms

Policy information about studies involving animals; ARRIVE guidelines recommended for reporting animal research

Laboratory animals	Laboratory mice (Mus Musculus) of C57BL/6 background were used in the study. Animal husbandry conditions included a room temperature of 23°C, humidity of 50%, and a 12-hour light–dark cycle (dark from 19:00 h to 07:00 h). Bedding in cages consisted of sawdust and wood shavings. Animals were housed with one to three cage mates. The wild type animals were compared to Satb1fl/flCd4-Cre+ animals. For majority of experiments, the animals were 4-8 weeks old. For flow cytometry experiments, characterization of the cytokine milieu and intraperitoneal glucose tolerance test, older animals 12-28 weeks old were also used for comparison as indicated in the manuscript. Animals used for genomics experiments were all females.
Wild animals	The study did not involve wild animals.
Field-collected samples	The study did not involve samples collected from the field.
Ethics oversight	All experiments were conducted in accordance with the Laboratory Animal Care and Ethics Committee of IMBB-FORTH. Animal work was approved by the IMBB Institutional Animal Care and Ethics Committee.

Note that full information on the approval of the study protocol must also be provided in the manuscript.

Flow Cytometry

Plots

Confirm that:

☒ The axis labels state the marker and fluorochrome used (e.g. CD4-FITC).

☒ The axis scales are clearly visible. Include numbers along axes only for bottom left plot of group (a 'group' is an analysis of identical markers).

☒ All plots are contour plots with outliers or pseudocolor plots.

☒ A numerical value for number of cells or percentage (with statistics) is provided.

Methodology

Sample preparation	Depending on the experiment, we used either thymocytes or splenocytes. Splenocytes were isolated in the same way as thymocytes, but they were further resuspended in plain water for 3 seconds to lyse erythrocytes, with immediate dilution by HBSS (Gibco, 14180) to a final 1X concentration. One million cell aliquots were distributed into 5 ml polystyrene tubes (BD Falcon 352052). For the experiments probing the percentage of apoptotic cells in tissues, we followed the PI/Annexin protocol (Biolegend, 640914). For staining with antibodies, we washed the cells once with Staining Buffer (1X PBS, 2% FBS,

0.1% NaN_3) and then stained in 100 µl of Staining Buffer with 1 µl of antibodies at 4°C for 30 minutes. The stained cells were washed with excess of Wash Buffer (1X PBS, 0.5% FBS) and then analyzed on FACSCalibur flow cytometer.

For analysis of T cell infiltration in the pancreas, pancreas was isolated from three WT and three Satb1 cKO mice of 120-136 days of age. Pancreas was cut in pieces and digested in 5 ml of 1 mg/ml collagenase (SIGMA, C2674) in PBS solution at 37°C for 30 minutes. Samples were washed twice with 5% FBS in PBS and filtered through a polypropylene mesh. After centrifugation, cell pellets were resuspended in 1 ml of 0.05% Trypsin solution and incubated for 5 minutes at 37°C. Cells were washed twice with ice-cold PBS and eventually filtered through a 40 µm cell strainer and blocked in 5 ml of 5% FBS in PBS for 30 minutes at 4°C. Cells were stained with 1:200 CD4-PE and CD8-APC for 30 minutes at 4°C and then washed twice with 0.5% FBS in 1X PBS. Lastly, cells were resuspended in 2% FBS in 1X PBS and analyzed by flow cytometry.

For characterization of the cytokine milieu, Cytokines were characterized and quantified from serum of 16 female mice (5 WT, 11 Satb1 cKO) of varying age 1-7 months by the LEGENDplex (13-plex) Mouse Th Cytokine Panel V02 (Biolegend, 740741; Lot B289245) according to the manufacturer's instructions.

For the SATB1 intracellular staining, thymocytes were first stained with CD4/CD8 as described earlier. Cells were then fixed for 10 minutes using 2% PFA (Pierce, 28908) in 1× PBS on ice. Fixed cells were washed with 1× PBS and then permeabilized using 0.3% Triton-X in 1× PBS for 5 minutes on ice. After three 5-minute washes in 1× PBS, cells were blocked for 30 minutes at room temperature with Blocking Buffer [0.4% acetylated BSA (Ambion, AM2614), 10% NGS and 0.1% Tween-20 in 4× SSC]. Staining for the primary antibody in question was performed using Detection Buffer (0.1% acetylated BSA and 0.1% Tween-20 in 4× SSC) using an antibody concentration of 0.5 µg per million of cells stained. Primary antibody staining was performed for 1 hour at room temperature. Following this, cells were washed thrice in Wash Buffer (0.1% Tween-20 in 4× SSC). Then, cells were incubated for 45 minutes at RT with a goat anti-mouse antibody conjugated with Alexa Fluor 647 (0.5 µg per million cells) in Detection Buffer. Finally, cells were washed thrice in Wash Buffer and following the final wash, cells were re-suspended in 1× PBS and analyzed on FACS Calibur flow cytometer.

Instrument

FACSCalibur flow cytometer.

Software

FlowJo v10.8.0 for data collection and FCSalyzer (version 0.9.17; https://sourceforge.net/projects/fcsalyzer) for data analysis.

Cell population abundance

No FACS sorting experiments are presented in this manuscript. Only flow cytometry analysis experiments are presented and the gating procedure is described accordingly as well as the percentage of each gated population.

Gating strategy

Experiments probing CD4/CD8/CD62L/CD44 populations were FSC/SSC gated for the expected size of lymphocytes (gate G1; Supplementary Fig. 3b). The CD62L/CD44 populations were additionally gated for CD4+ cells (gate G2; Supplementary Fig. 3b). For the experiments with Annexin V and PI staining, the gating was set to exclude cellular debris (gate G3; Supplementary Fig. 3b).

☒ Tick this box to confirm that a figure exemplifying the gating strategy is provided in the Supplementary Information.
